# CdS-Based Photocatalysts for Antimicrobial Applications: From Quantum Dots to Z-Scheme Heterojunctions—Mechanisms, Challenges, and Future Perspectives

**DOI:** 10.3390/molecules31152626

**Published:** 2026-07-28

**Authors:** Nurlan Almas, Mirat Karibayev, Saparbek Tugelbay, Aliya Assilbekova, Irina Irgibaeva, Nursultan Mussakhanuly, Sergei Piskunov, Galiya Baisalova, Anuar Aldongarov

**Affiliations:** 1 Department of Chemistry, L.N. Gumilyov Eurasian National University, Astana 010000, Kazakhstan; nurlanalmasov@gmail.com (N.A.); asylaliya@yandex.kz (A.A.); irgsm@mail.ru (I.I.); 2 National Laboratory Astana, Nazarbayev University, Astana 010000, Kazakhstan; mirat.karibayev@nu.edu.kz (M.K.); saparbek.tugelbay@nu.edu.kz (S.T.); 3 Institute of New Materials and Energy Technologies, Nazarbayev University, Astana 010000, Kazakhstan; nursultan.mussakhanuly@nu.edu.kz; 4 School of Intellectual Systems, Astana IT University, EXPO C1, Mangilik El 55/11, Astana 010000, Kazakhstan; 5 Institute of Solid State Physics, University of Latvia, LV-1063 Riga, Latvia

**Keywords:** cadmium sulfide, photocatalysis, antibacterial activity, nanocomposites, reactive oxygen species, heterojunction, multidrug resistance, visible-light photocatalyst, photocorrosion, nanostructured materials

## Abstract

The chronic overuse of antibiotics has accelerated the emergence of antibiotic-resistant bacteria, creating a global public health crisis as conventional therapies fail against multidrug-resistant pathogens spreading through water and food chains. Cadmium sulfide (CdS) has been established as an important visible-light-driven photocatalyst for antibacterial applications. This brief review systematically examines the structure–property relationships governing CdS-based antibacterial materials, including crystallographic polymorphs (cubic sphalerite and hexagonal wurtzite), morphological diversity from quantum dots to hierarchical architectures, and synthesis methodologies that critically influence particle size, crystallinity, and surface chemistry. The mechanisms of antibacterial action are elucidated, encompassing photocatalytic reactive oxygen species (ROS) generation, controlled Cd^2+^ ion release, and membrane disruption. A detailed tabulated analysis is presented across three material classes: pristine CdS, binary composites, and ternary Z-scheme heterostructures. Density functional theory (DFT) calculations and molecular docking simulations provide atomic-level insights into charge transfer dynamics and enzyme inhibition mechanisms. Finally, critical challenges, photocorrosion, toxicity, biocompatibility concerns, and scalability limitations are addressed. This review bridges fundamental materials science with antimicrobial applications to guide rational design of next-generation CdS-based antibacterial materials.

## 1. Introduction

### 1.1. Overview of CdS Photocatalysts for Antibacterial Activity

The increasing prevalence of antibiotic-resistant bacteria has emerged as a critical global health crisis, posing a major challenge to public health systems and medical research [1]. Over the past few decades, the overuse and misuse of antibiotics have accelerated the development of multidrug-resistant (MDR) bacterial strains, rendering many conventional treatments ineffective. The World Health Organization (WHO) has recognized antibiotic resistance as one of the foremost global threats to human health. Projections indicate that by 2050, antimicrobial resistance could surpass cancer as a leading cause of mortality worldwide. Fatalities from untreatable infections could increase tenfold unless alternative antimicrobial strategies are urgently developed [2,3,4]. While conventional antibiotics retain some efficacy against susceptible pathogens, none possess complete activity against MDR bacteria. This challenge necessitates the urgent development of alternative antibacterial materials that can effectively combat pathogenic microorganisms while minimizing resistance development.

**Figure 1:**
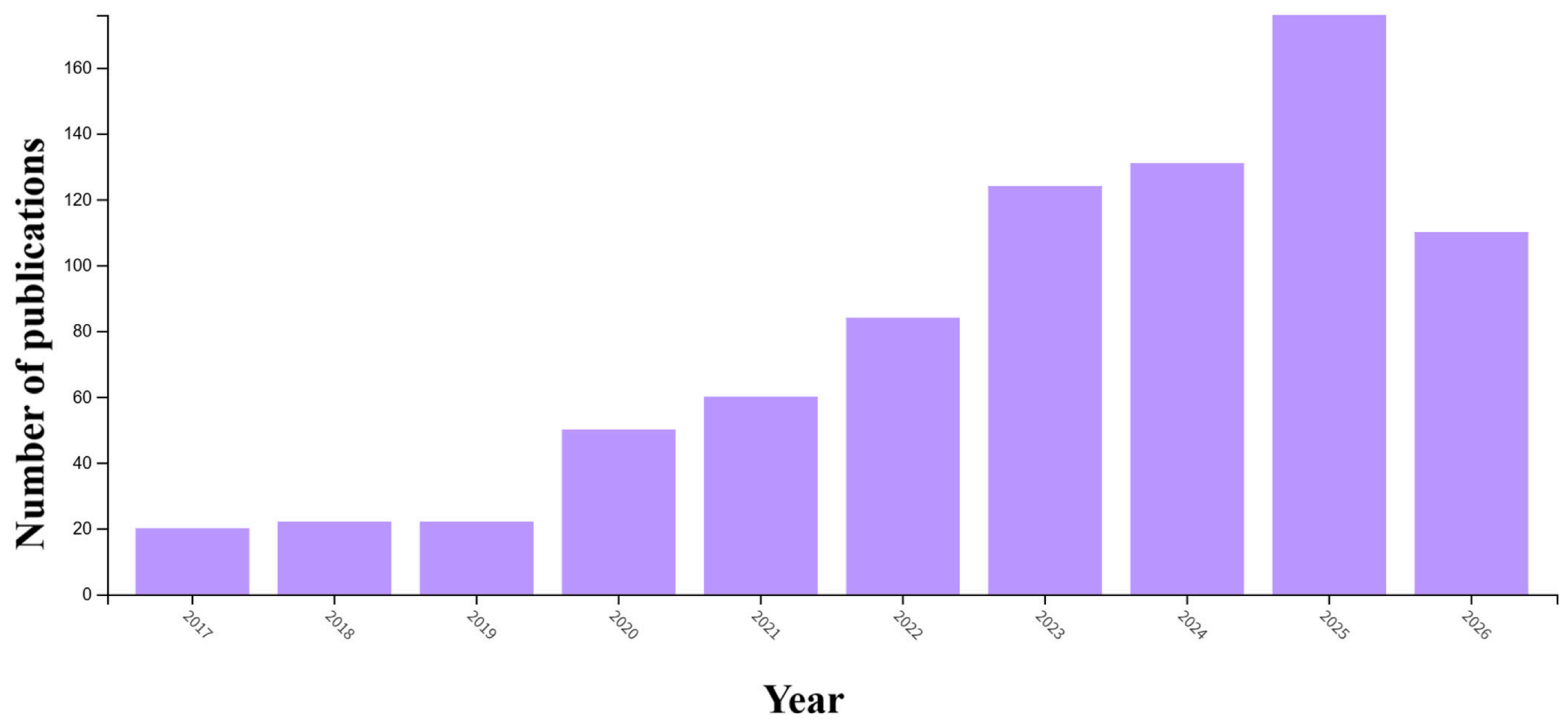
Bibliometric analysis of CdS-based antibacterial research publications from 2017 to 2026. Data retrieved from Web of Science (https://www.webofscience.com/, accessed on 8 July 2026) using the search query “CdS” AND “antibacterial activity”.

In this context, inorganic semiconductor-based photocatalysts have garnered significant attention as a promising platform for next-generation antimicrobial therapy. Among the diverse array of photocatalytic materials, cadmium sulfide (CdS) has been established as an important antimicrobial photocatalyst (Figure 1).

Bibliometric analysis of the Web of Science database using the keywords “CdS” and “antibacterial activity” reveals a notable growth trajectory in this research field (Figure 1). From modest beginnings with 19 publications in 2018, the field experienced exponential growth, reaching a peak of around 170 in 2025. This trend reflects the increasing recognition of CdS-based photocatalysts as visible-light-driven antibacterial agents, driven by advances in heterojunction engineering, morphological control, and understanding of reactive oxygen species (ROS)-mediated mechanisms.

As an II-VI semiconductor with a direct bandgap of approximately 2.42 eV, CdS can absorb visible light, which constitutes the major fraction of the solar spectrum. This property circumvents the need for costly and impractical ultraviolet excitation. Upon illumination with photons of energy equal to or exceeding its bandgap, electrons are promoted from the valence band to the conduction band, leaving behind positively charged holes [5,6,7,8]. These photogenerated charge carriers migrate to the material’s surface, where they participate in redox reactions with ambient water and molecular oxygen. These reactions produce ROS, including hydroxyl radicals (•OH), superoxide anions (O_2_•^−^), and hydrogen peroxide (H_2_O_2_).

These ROS are highly cytotoxic, capable of inducing oxidative stress, disrupting membrane integrity through lipid peroxidation, inactivating essential enzymes, and causing irreparable deoxyribonucleic acid (DNA) damage in microbial cells [8,9,10]. This multitargeted mechanism simultaneously attacks multiple cellular components, making it difficult for bacteria to develop resistance. CdS-based photocatalysis is therefore effective against both planktonic bacteria and structurally robust biofilms. Unlike conventional antibiotics that typically interfere with a single biochemical pathway, the oxidative stress induced by photocatalytic ROS represents a universal mechanism of cell death to which broad-spectrum resistance is unlikely to emerge.

Beyond its intrinsic photocatalytic properties, the antibacterial efficacy of CdS is profoundly influenced by its structural and morphological characteristics. As detailed in subsequent sections, CdS exists in two primary crystallographic polymorphs, cubic sphalerite and hexagonal wurtzite, each exhibiting distinct electronic band structures and surface properties that modulate photocatalytic activity. Furthermore, the ability to engineer CdS across multiple length scales, from zero-dimensional quantum dots exhibiting pronounced quantum confinement effects to three-dimensional hierarchical architectures with exceptionally high surface areas, provides unparalleled opportunities to optimize light absorption, charge separation efficiency, and intimate bacterial interaction [11,12,13]. However, the widespread adoption of pristine CdS is hampered by two significant challenges: pronounced photocorrosion under prolonged illumination, which leads to the oxidation of lattice S^2−^ ions and consequent material degradation, and rapid electron–hole recombination that diminishes quantum efficiency. These limitations have catalyzed intensive research into advanced stabilization strategies, particularly the construction of CdS-based composites with complementary materials.

### 1.2. Scope and Organization of This Review

Existing reviews on semiconductor-based antimicrobial materials have primarily focused on broad classes of photocatalysts without systematically addressing CdS-based systems’ advantages and challenges (Section 10). These include visible-light responsiveness, favorable band edge positions, and the critical issue of photocorrosion. Similarly, reviews on photocatalytic disinfection [9,10] have largely overlooked the specific structure–property relationships governing CdS antibacterial activity. These relationships include the influence of crystallographic polymorphs, morphological diversity, and heterojunction architecture on ROS generation and bacterial inactivation kinetics. Furthermore, no existing review provides a comprehensive tabulated analysis of antibacterial performance across pristine CdS, binary composites, and ternary heterostructures, nor integrates experimental findings with atomistic modeling insights from density functional theory (DFT) calculations and molecular docking simulations. A detailed comparative analysis of previous reviews and the specific contribution of the present manuscript is provided in Section 10.

The scope of this review encompasses a comprehensive examination of CdS-based antibacterial materials, beginning with a foundational understanding of their structural and electronic characteristics. Section 2 covers the crystallographic polymorphs, electronic properties, morphological diversity, photocorrosion, phase instability, and toxicity of CdS, providing the foundational understanding for its photocatalytic applications and the strategies to mitigate its limitations. Section 3 provides an overview of synthesis methodologies, correlating preparation techniques with the resulting material properties and their implications for antibacterial performance. Recognizing that bacterial cell structure profoundly influences susceptibility, Section 4 briefly discusses the key structural and functional differences among Gram-positive, Gram-negative, and fungal pathogens. The core of the review is presented in Section 5, Section 6, Section 7 and Section 8, which systematically evaluate the mechanisms of antibacterial action and provide a comprehensive tabulated analysis of the antibacterial performance of pristine CdS, binary composites, and ternary heterostructures.

It is important to note that while the distinction between light-dependent photocatalytic activity and dark-active ion release mechanisms is conceptually important for evaluating the multifunctionality of CdS-based materials, the available literature does not consistently report whether antibacterial tests were conducted under dark, light, or both conditions. Consequently, a definitive assignment of activity modes across the summarized studies remains challenging. This limitation highlights the need for future studies to adopt standardized testing protocols that include systematic dark and light controls, enabling unambiguous identification of truly dual-mode antibacterial systems.

Finally, Section 9 offers atomistic studies of CdS for antibacterial activity, and Section 10 offers a critical synthesis of the current state of the field, identifying key challenges, unresolved questions, and future research directions.

## 2. The Properties of CdS

### 2.1. Crystallographic Polymorphs of CdS

**Figure 2:**
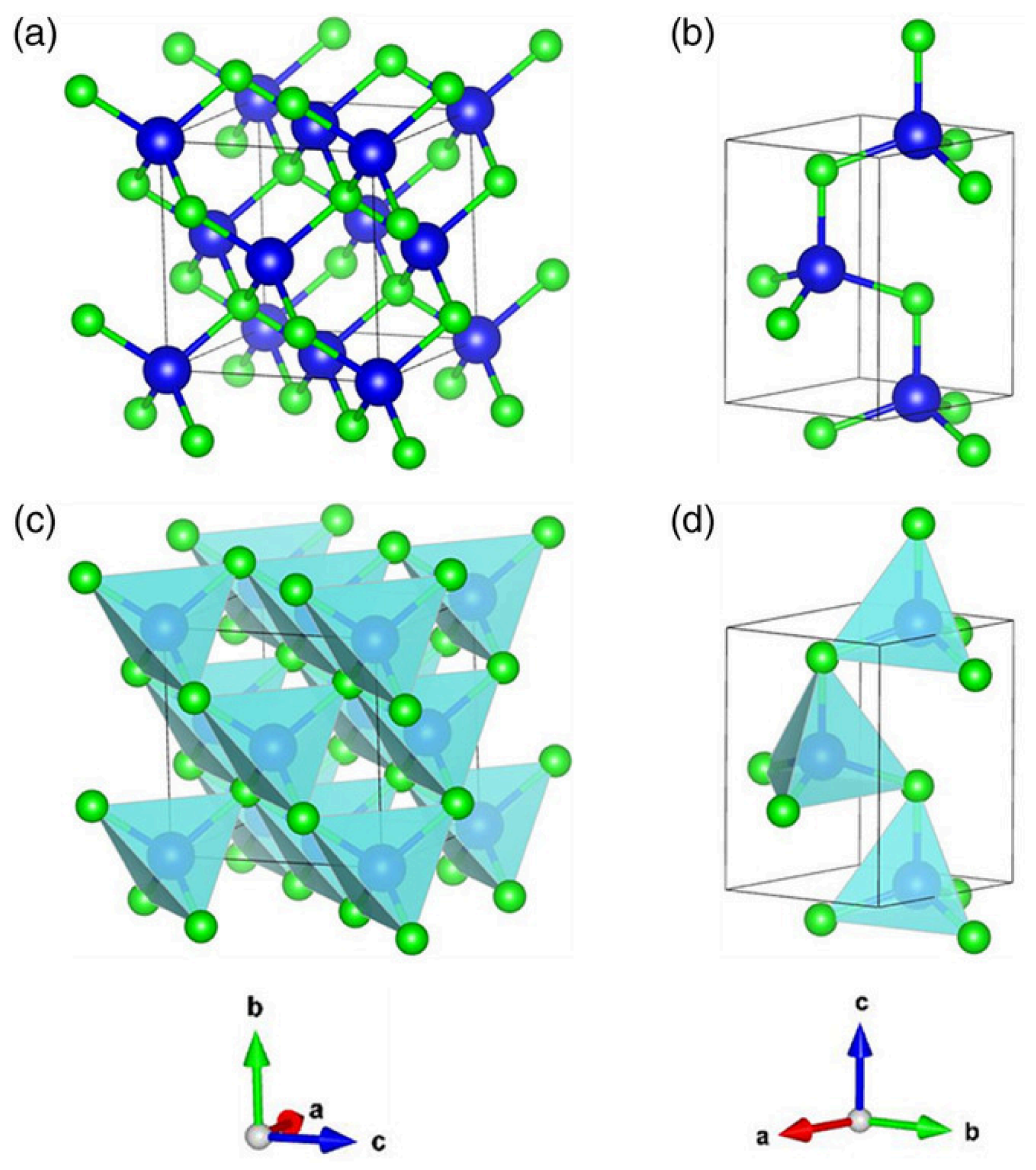
Ball-and-stick representations of the cubic (**a**) and hexagonal (**b**) crystal structures of CdS. Orthotetrahedral Cd-S coordination units in the cubic (**c**) and hexagonal (**d**) CdS unit cells. Blue and green spheres denote Cd and S atoms, respectively. Reprinted with permission from [21] (CC BY license).

CdS is predominantly found in two distinct crystallographic polymorphs: the cubic sphalerite (zincblende) structure, belonging to the space group $F\bar{4}3m$ with a lattice parameter of a ≈ 5.82 Å, and the hexagonal wurtzite structure, characterized by the space group $P6_{3}mc$ and lattice parameters a ≈ 4.136 Å and c ≈ 6.756 Å (Figure 2a–d) [14,15,16,17]. While the wurtzite phase is thermodynamically stable under ambient conditions, the sphalerite phase exists in a metastable state and is most observed in nanoparticle or thin-film morphologies.

In the cubic sphalerite phase, each Cd^2+^ ion is tetrahedrally coordinated to four S^2−^ ions, forming a three-dimensional network of corner-sharing CdS_4_ units with bond lengths of approximately 2.52–2.54 Å. This phase exhibits a direct bandgap of roughly 2.42 eV, where the valence band is primarily derived from S 3p orbitals and the conduction band from Cd 5s orbitals, a configuration that facilitates efficient visible-light absorption. The hexagonal wurtzite phase maintains a similar tetrahedral coordination (CdS_4_) but distinguishes itself through an ABAB close-packing sequence along the crystallographic c-axis. With a c/a ratio of approximately 1.623 and comparable Cd-S bond lengths, this phase also possesses a direct bandgap of about 2.42 eV in the bulk, which can increase to 2.5 eV in nanostructures due to quantum confinement. This electronic structure, arising from the hybridization of Cd (5s/5p) and S (3p) orbitals, is particularly advantageous for promoting charge separation in photocatalytic applications.

Both crystalline phases lack inversion symmetry, a feature that endows them with piezoelectric properties. Furthermore, the polar surfaces prevalent in nanostructured CdS generate internal electric fields that effectively separate photogenerated electron–hole pairs, thereby enhancing their utility in photocatalysis. A thermally driven phase transition from the metastable cubic sphalerite to the stable hexagonal wurtzite is typically observed upon annealing above 300 °C. It is important to note that while no stable tetragonal phase exists for bulk CdS, amorphous or mixed-phase configurations can appear as transient intermediates during synthesis.

### 2.2. Bandgap of CdS

CdS is a direct bandgap II-VI semiconductor with a room-temperature bandgap energy of approximately 2.42 eV for bulk material, a characteristic that underpins its strong visible-light absorption. The electronic transitions in CdS are direct and occur at the Γ-point of the Brillouin zone, where the valence band maximum (VBM) is primarily composed of S 3p orbitals hybridized with Cd 5s and 5p states, while the conduction band minimum (CBM) is predominantly derived from Cd 5s orbitals. This electronic configuration is further shaped by strong p-d hybridization between S 3p and Cd 4d orbitals near the VBM, which contributes to the material’s covalent bonding character and its high absorption coefficient [15,16,17,18,19,20]. The dispersive nature of the Cd 5s-derived CBM facilitates efficient electron transport, whereas the more localized character of the VBM promotes hole stability, a feature particularly advantageous for photocatalytic applications.

The bandgap of CdS is highly sensitive to both structural and dimensional parameters. In nanoparticles, quantum confinement effects induce a notable blueshift, with bandgap energies increasing up to 2.56 eV for extremely small crystallites. Conversely, nonstoichiometric variations, such as Cd-rich system, introduce shallow donor levels associated with interstitial cadmium below the CBM, effectively narrowing the bandgap to approximately 2.21 eV and extending light absorption into the longer wavelength region [21,22,23]. Additionally, a slight structural dependence is observed, with the hexagonal wurtzite phase typically exhibiting a marginally wider bandgap (~2.45 eV) compared to its cubic sphalerite counterpart (~2.40 eV). These tunable electronic attributes, combined with efficient charge separation capabilities, render CdS an exceptional material for applications spanning photovoltaics, photocatalysis, and optoelectronic devices.

### 2.3. Morphology of CdS

The morphological landscape of CdS is remarkably rich, encompassing zero-dimensional (0D), one-dimensional (1D), two-dimensional (2D), and three-dimensional (3D) architectures, each exhibiting unique physicochemical properties that can be strategically harnessed for antibacterial applications.

Zero-dimensional morphologies, including spherical nanoparticles and quantum dots typically ranging from 2 to 50 nm in size, are commonly synthesized via chemical precipitation or colloidal methods. In this regime, quantum confinement effects become pronounced, inducing a significant blueshift in the bandgap, from the bulk value of 2.42 eV up to approximately 3 eV for the smallest quantum dots, thereby enhancing photoluminescence properties suitable for optoelectronic and bioimaging applications [21,22,23,24,25]. These 0D structures generally crystallize in either cubic or hexagonal phases and serve as fundamental building blocks for more complex architecture.

One-dimensional CdS nanostructures, such as nanorods (typically 40–90 nm in diameter and 1–3 μm in length) and nanowires, are frequently fabricated through solvothermal treatment or chemical bath deposition, often adopting the zincblende structure. The primary advantage of 1D morphologies lies in their ability to facilitate directional charge transport along the longitudinal axis, which minimizes electron–hole recombination and enhances photocatalytic efficiency, a critical feature for antibacterial mechanisms relying on ROS generation. Interestingly, transmission electron microscopy studies have revealed that beam-induced melting can transform nanorods into thinner nanowires without altering the underlying crystalline phase, offering potential pathways for morphological engineering [22,23,24,25,26].

Two-dimensional CdS morphologies, encompassing nanosheets, nanoplatelets, and hexagonal disks with lateral dimensions of 50–300 nm and thicknesses of only a few nanometers, emerge from controlled heating-up syntheses that promote anisotropic growth along specific basal planes. This layer-stacking morphology not only enables integration into thin-film devices but also improves charge separation through reduced charge migration distances, making them particularly attractive for photovoltaic and photocatalytic applications where efficient carrier extraction is paramount [23,24,25,26].

Three-dimensional hierarchical structures represent the pinnacle of morphological complexity, featuring flower-like assemblies (250–400 nm), pyramid-like formations (300–400 nm base), sponge-like networks (200–300 nm), and multipod or bipod configurations. These architectures typically form under high Cd:S ratios in millifluidic or precipitation synthesis routes and are characterized by exceptionally high specific surface areas. This enhanced surface area provides abundant active sites for photocatalytic ROS generation and facilitates intimate contact with bacterial membranes, thereby amplifying antibacterial efficacy. The hierarchical organization also promotes multiple light scattering within the structure, maximizing photon absorption and further boosting photocatalytic activity. Collectively, this morphological diversity enables tailored design of CdS-based photocatalysts for multimodal antibacterial therapy, where factors such as surface area, charge separation efficiency, and ROS generation capacity can be optimized through precise morphological control [25,26,27,28,29].

### 2.4. Photocorrosion and Phase Instability of CdS

CdS faces significant challenges limiting its long-term antibacterial applications by photocorrosion/phase instability. The metastable cubic sphalerite phase undergoes irreversible transformation to the thermodynamically stable hexagonal wurtzite phase upon annealing at 300–370 °C, restricting high-temperature processing [30,31,32,33]. More critically, prolonged visible-light illumination triggers photocorrosion, where photogenerated holes oxidize lattice S^2−^ ions to elemental sulfur or soluble sulfates, progressively degrading structural integrity and photocatalytic performance. This anodic degradation is particularly severe in aqueous environments where sluggish oxygen evolution kinetics fail to scavenge photogenerated holes efficiently.

The photocorrosion of CdS proceeds through well-defined anodic pathways that vary depending on reaction conditions. In the absence of oxygen, photogenerated holes oxidize surface sulfide ions to elemental sulfur via the following reaction: CdS + 2h^+^ → Cd^2+^ + S^0^. However, in oxygenated aqueous environments, the corrosion pathway shifts toward sulfate formation: CdS + 4h^+^ + 2H_2_O + O_2_ → Cd^2+^ + SO_4_^2−^ + 4H^+^ [4,30]. Advanced surface spectroscopic studies have confirmed that sulfur species undergo stepwise oxidation from S^2−^ (binding energy ~161.5 eV) to S^0^ (~163.5 eV) and finally to SO_4_^2−^ (~168 eV), with the presence of oxygen dramatically accelerating the corrosion rate through reductive dissolution pathways. Notably, this degradation is not limited to particulate systems; thin-film CdS photoanodes coated with protective TiO_2_ layers (even 7 nm thick) exhibit characteristic pinhole-initiated corrosion following Avrami-type kinetics, where corrosion propagates laterally from defect sites and shows oscillatory photocurrent behavior as corrosion pits nucleate, grow, and coalesce [8,31]. The identification of such mechanistic details is critical for rational design of protection strategies, as it highlights that even ostensibly conformal coatings can fail if pinhole defects are not eliminated [10,32].

To overcome these limitations, four complementary stabilization strategies have been developed including (i) heterojunction engineering, (ii) surface passivation and encapsulation, (iii) elemental doping, and (iv) morphological engineering.

While each of these approaches contributes to mitigating photocorrosion to varying degrees, heterojunction engineering has emerged as the most extensively investigated, with type-II, Z-scheme, and S-scheme architectures offering distinct advantages for charge separation and photocorrosion suppression [30,31].

The strategic coupling of CdS with wide-bandgap semiconductors or conductive supports serves dual functions: it promotes spatial separation of photogenerated charges, reducing the hole concentration available for sulfur oxidation, and provides physical protection through interfacial passivation [4,8,32].

For instance, CoP-modified CdS type I heterojunctions achieved improved photocatalytic stability, retaining 93% of initial activity after five cycles of simulated solar irradiation. Similarly, core–shell architecture provides robust physical barriers against electrolyte contact while simultaneously enabling efficient charge transfer through carefully engineered interfaces [4,8,10,30,31,32].

As illustrated in Table 1, different heterojunction architectures exhibit distinct charge transfer pathways. In a type II heterojunction, photogenerated electrons and holes migrate to different semiconductors, leading to efficient spatial charge separation but simultaneously reducing the redox potential because both charge carriers accumulate in lower-energy bands [30,31,32,33]. In contrast, Z-scheme heterojunctions preserve the highly reducing electrons and highly oxidizing holes by allowing for the recombination of low-energy charge carriers at the interface, thereby maintaining strong redox capability while improving charge separation. The recently developed S-scheme heterojunction further refines this concept through the formation of an internal electric field and band bending at the interface. This built-in electric field selectively promotes the recombination of photogenerated carriers with weak redox ability while retaining electrons and holes with the highest reduction and oxidation potentials, respectively. Consequently, S-scheme heterojunctions combine efficient charge separation with strong redox activity, making them particularly attractive for CdS-based antibacterial photocatalysts, where enhanced ROS generation and reduced photocorrosion are essential for achieving sustained antimicrobial performance.

### 2.5. Toxicity of CdS

Cadmium toxicity represents a significant barrier to the practical implementation and clinical translation of CdS-based antibacterial materials. As a Group 1 carcinogen and highly regulated heavy metal, cadmium poses serious concerns regarding environmental release, bioaccumulation, and human health risks. The primary source of toxicity arises from the gradual release of Cd^2+^ ions during photocatalytic operation, particularly under aqueous conditions where photocorrosion accelerates dissolution. Importantly, the rate and extent of Cd^2+^ leaching are highly dependent on the composite architecture, with different heterojunction designs exhibiting distinctly different ion release profiles, as discussed in detail in Section 10.

These architectural differences fundamentally determine the risk–benefit profile of CdS-based materials and must be systematically evaluated for each proposed application. For wound healing or implant-related applications, sustained low-level Cd^2+^ release could delay tissue regeneration or induce chronic inflammatory responses, while acute high-level release may cause immediate cytotoxicity. The European Union’s Registration, Evaluation, Authorization and Restriction of Chemicals (REACH) regulation and the United States Environmental Protection Agency (U.S. EPA) drinking water standards impose stringent limits on cadmium concentrations (e.g., EPA maximum contaminant level of 5 µg/L) [9,10,11,12,13,14,15]. Furthermore, the long-term environmental fate of CdS, including transformation, degradation, and trophic transfer through food chains, remains poorly understood. Recent studies have demonstrated that CdS can accumulate in aquatic organisms and undergo photochemical transformations that alter their bioavailability and toxicity profiles [11,12,13,14,15,16].

Addressing these nanotoxicological uncertainties requires systematic in vitro and in vivo studies that evaluate not only acute cytotoxicity but also chronic exposure effects, biodistribution, organ-specific accumulation, and clearance mechanisms. The integration of protective coatings, controlled-release strategies, and biodegradable encapsulation materials offers promising avenues to mitigate toxicity while preserving antibacterial efficacy. Until comprehensive toxicological profiling and risk assessment are conducted, clinical applications of CdS-based materials should be approached with appropriate caution, and environmental applications should incorporate containment and recovery strategies to prevent uncontrolled release.

## 3. Synthesis Methods of CdS

**Figure 3:**
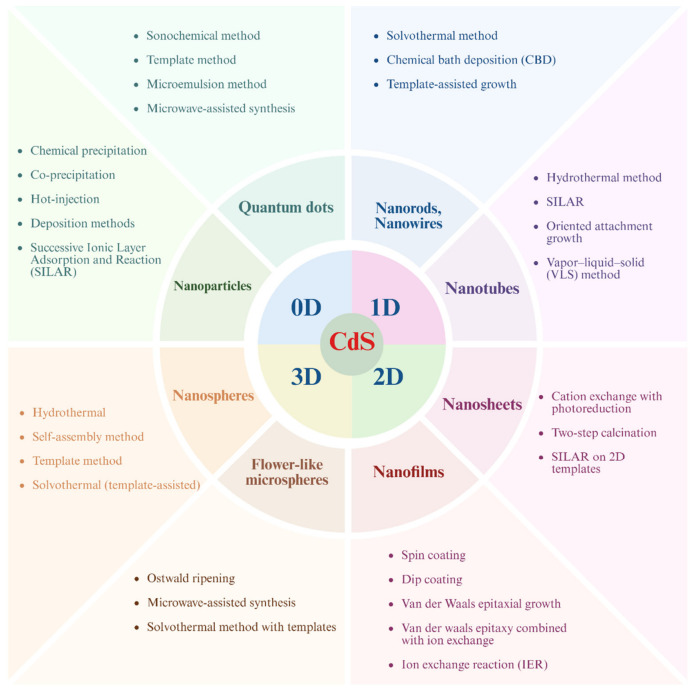
A schematic illustration of the synthesis methods of CdS. Created in BioRender. Tugelbay, S. (2026); https://BioRender.com/w8jr6mg, accessed on 8 July 2026.

The synthesis route plays a decisive role in determining the crystallinity, morphology, particle size, and surface characteristics of CdS photocatalysts, all of which directly influence visible-light absorption, charge separation, ROS generation, and ultimately antibacterial performance (Figure 3) [34,35,36,37]. The most common preparation methods include hydrothermal/solvothermal synthesis, chemical bath deposition (CBD), successive ionic layer adsorption and reaction (SILAR), sonochemical synthesis, and template-assisted approaches.

Hydrothermal and solvothermal methods are widely employed to produce highly crystalline CdS nanostructures with controlled morphologies. By adjusting reaction temperature, solvent, and precursor concentration, these techniques enable the formation of nanorods, nanoparticles, and hierarchical architectures with enhanced charge transport and photocatalytic activity [35,36,37,38]. In contrast, CBD offers a simple and low-cost route for depositing uniform CdS coatings on various substrates, while SILAR enables layer-by-layer growth with precise control over film thickness and composition, making both methods attractive for fabricating CdS thin films and heterojunctions [37,38,39,40].

Alternative methods, including sonochemical and template-assisted synthesis, provide access to complex nanostructures such as nanosheets, hollow spheres, and hierarchical architectures. These structures offer increased surface area and abundant active sites, which can further improve photocatalytic antibacterial efficiency [41,42,43].

In this regard, the synthesis strategy determines the structural features that govern light harvesting, charge-carrier dynamics, and surface reactivity, thereby influencing the antibacterial performance of CdS-based materials. Because comprehensive reviews of CdS synthesis are already available, this review does not provide an exhaustive discussion of preparation techniques. Instead, emphasis is placed on how synthesis-induced structural characteristics affect the antibacterial properties of pristine CdS, binary composites, and advanced heterojunctions discussed in the following sections.

## 4. Differences in Structure and Function Among Gram-Positive Bacteria, Gram-Negative Bacteria, and Fungal Pathogens

**Figure 4:**
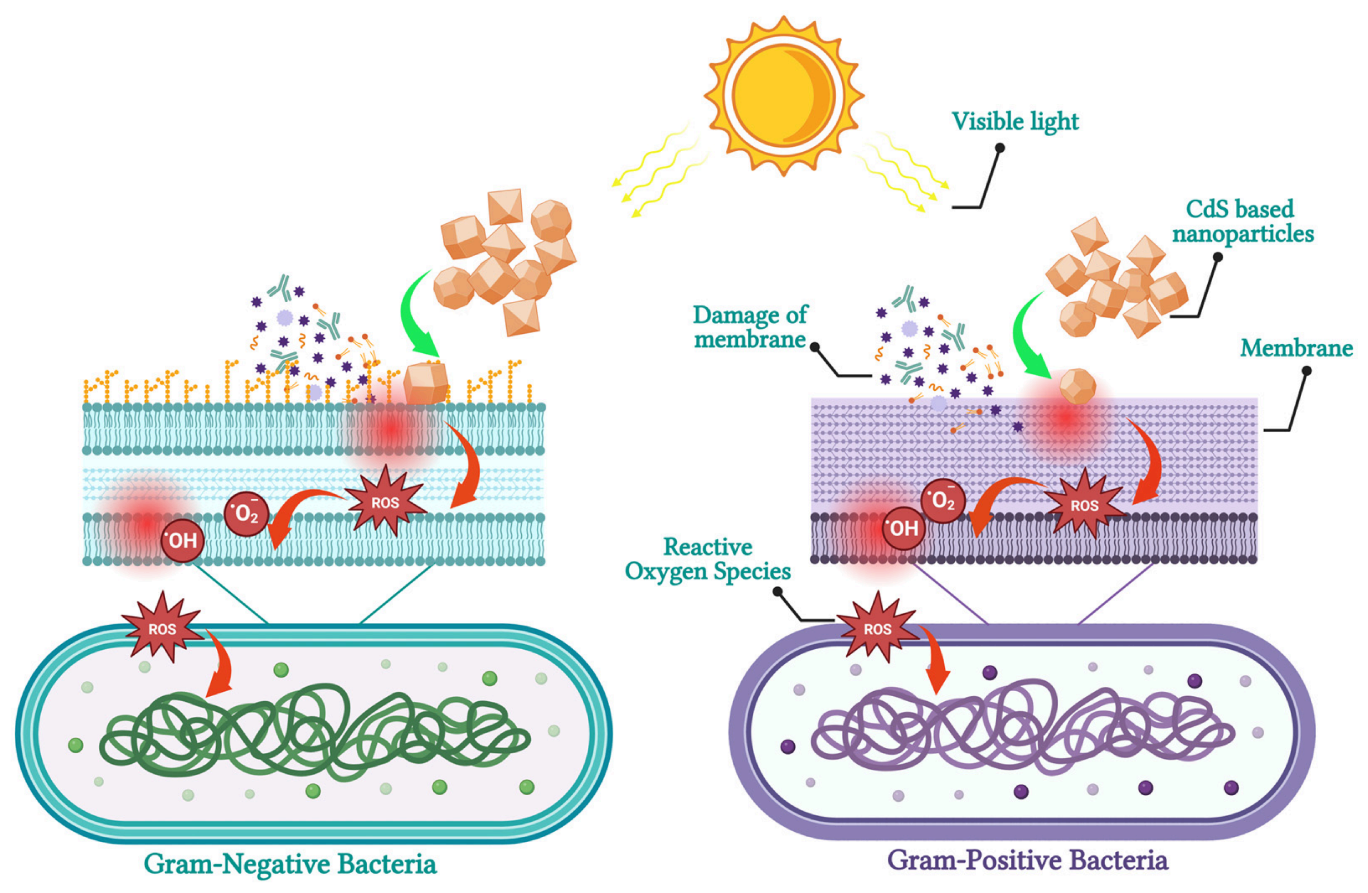
Comparative antibacterial mechanisms of CdS-based photocatalysts against Gram-negative and Gram-positive bacteria under visible-light irradiation. Created in BioRender. Tugelbay, S. (2026); https://BioRender.com/mlfr3mp, accessed on 8 July 2026.

The structural composition and physiological characteristics of microbial pathogens play a decisive role in determining their susceptibility to antimicrobial agents, including semiconductor nanomaterials such as CdS-based photocatalyst (Figure 4). Gram-positive bacteria, Gram-negative bacteria, and fungal pathogens possess distinct cell wall architectures and membrane compositions that regulate the penetration, accumulation, and activity of ROS and metal ions generated by CdS photocatalysts [44,45,46]. These structural disparities ultimately influence the antibacterial and antifungal efficiency of CdS-based photocatalysts and necessitate the development of tailored multimodal therapeutic strategies.

Gram-positive bacteria are characterized by a thick peptidoglycan layer typically ranging from 20 to 80 nm in thickness, which provides structural rigidity and mechanical protection against osmotic stress [45,46,47,48]. This layer consists of cross-linked N-acetylglucosamine and N-acetylmuramic acid units forming a dense polymeric network around the cytoplasmic membrane. In addition, teichoic and lipoteichoic acids embedded within the peptidoglycan matrix contribute to the overall negative charge of the cell wall and play a role in metal ion binding.

Unlike Gram-negative bacteria, Gram-positive species lack an outer membrane, which generally makes them more susceptible to antimicrobial nanoparticles and metal ions [46,47,48]. The relatively porous nature of the peptidoglycan layer allows small CdS particles, particularly those below 20 nm, to penetrate the cell wall and interact directly with intracellular components. Upon visible-light irradiation, CdS particles generate ROS such as •OH and singlet oxygen (^1^O_2_), which induce oxidative damage to cellular macromolecules. These ROS can disrupt peptidoglycan cross-linking, deactivate autolysins, and damage cytoplasmic membranes, ultimately leading to bacterial cell death.

Consequently, Gram-positive pathogens such as *Staphylococcus aureus*, *Streptococcus pneumoniae*, and *Bacillus subtilis* often exhibit high susceptibility to photocatalytic antimicrobial materials [46,47,48,49]. However, compared with Gram-negative bacteria, the absence of LPS layers may limit certain photothermal interactions, reducing the synergistic enhancement of antibacterial activity under near-infrared irradiation.

Gram-negative bacteria possess a more complex cell envelope structure that provides enhanced protection against external antimicrobial agents [47]. Their cell wall consists of a thin peptidoglycan layer, typically 2–7 nm thick, located within the periplasmic space between an inner cytoplasmic membrane and an outer membrane. The outer membrane is enriched with LPS, phospholipids, and porin proteins that regulate the selective transport of molecules into the cell.

The LPS layer forms a highly electronegative barrier that can hinder the penetration of cationic nanoparticles and metal ions. In addition, porin channels generally allow only small molecules (approximately 2 nm in diameter) to pass through, further limiting the uptake of larger nanomaterials [48]. Efflux pumps located within the membrane can also actively expel toxic compounds, contributing to the intrinsic resistance of Gram-negative pathogens such as *Escherichia coli*, *Pseudomonas aeruginosa*, *Klebsiella pneumoniae*, and *Salmonella* enterica.

To overcome these barriers, CdS-based nanocomposites are often engineered with reduced particle sizes or integrated with additional functional materials. For example, heterojunction structures such as CdS/ZnO or CdS/graphene oxide can enhance membrane interaction and facilitate penetration through the outer membrane [49]. In some cases, released Cd^2+^ or Zn^2+^ ions can interact with LPS molecules, destabilizing the membrane and increasing permeability. Moreover, graphene oxide/wrapped CdS nanostructures may inhibit bacterial efflux pumps and sustain intracellular ROS levels, thereby amplifying oxidative stress and leading to efficient bacterial inactivation even in biofilm-forming species.

Fungal pathogens differ fundamentally from bacteria due to their eukaryotic cellular structure and the presence of a multilayered cell wall composed primarily of chitin, β-glucans, and mannoproteins [50]. This wall is significantly thicker than bacterial cell walls, typically ranging from 100 to 300 nm, and provides strong mechanical protection against environmental stress. Beneath the cell wall lies a plasma membrane rich in ergosterol, a sterol component unique to fungal cells.

These structural features contribute to the inherent resistance of fungal organisms to many antimicrobial agents [51]. Opportunistic pathogens such as Candida albicans, *Aspergillus niger*, and Cryptococcus neoformans also exhibit morphological plasticity, including yeast-to-hypha transitions and biofilm formation, which further enhances their survival and pathogenicity.

Despite these protective mechanisms, photocatalytic nanomaterials such as CdS-based composites can exert antifungal effects through ROS-mediated oxidative stress. Generated ROS can penetrate fungal membranes and induce lipid peroxidation, particularly targeting ergosterol molecules and disrupting membrane integrity [52]. This oxidative damage may also affect mitochondrial function, DNA stability, and enzymatic processes involved in cell wall biosynthesis.

However, fungal cells also possess antioxidant defense systems, including glutathione and catalase enzymes, which can neutralize ROS and reduce antimicrobial efficacy. Therefore, multimodal approaches combining photocatalytic ROS generation with photothermal heating or controlled metal ion release are often required to achieve effective fungal inactivation. For instance, CdS-based nanocomposites incorporating noble metals or heterojunction semiconductors can simultaneously generate ROS and induce localized photothermal effects, thereby enhancing antifungal performance and inhibiting hyphal growth.

The substantial structural and compositional differences among Gram-positive bacteria, Gram-negative bacteria, and fungal pathogens highlight the importance of designing CdS-based photocatalysts with adaptable antimicrobial mechanisms. Gram-positive bacteria are generally more susceptible to direct ROS-mediated damage due to their porous cell walls, whereas Gram-negative pathogens require strategies that overcome outer membrane barriers and efflux mechanisms. Fungal pathogens, in contrast, demand stronger multimodal interventions due to their thick chitinous cell walls and antioxidant defense systems [53].

Therefore, future CdS photocatalyst designs should focus on optimizing nanoparticle size, surface functionalization, heterojunction formation, and bandgap engineering to enhance ROS generation and cellular penetration across diverse microbial species. Such tailored approaches will be critical for developing broad-spectrum antimicrobial systems capable of addressing the growing challenge of antimicrobial resistance.

## 5. Mechanisms of Antibacterial Action of CdS

**Figure 5:**
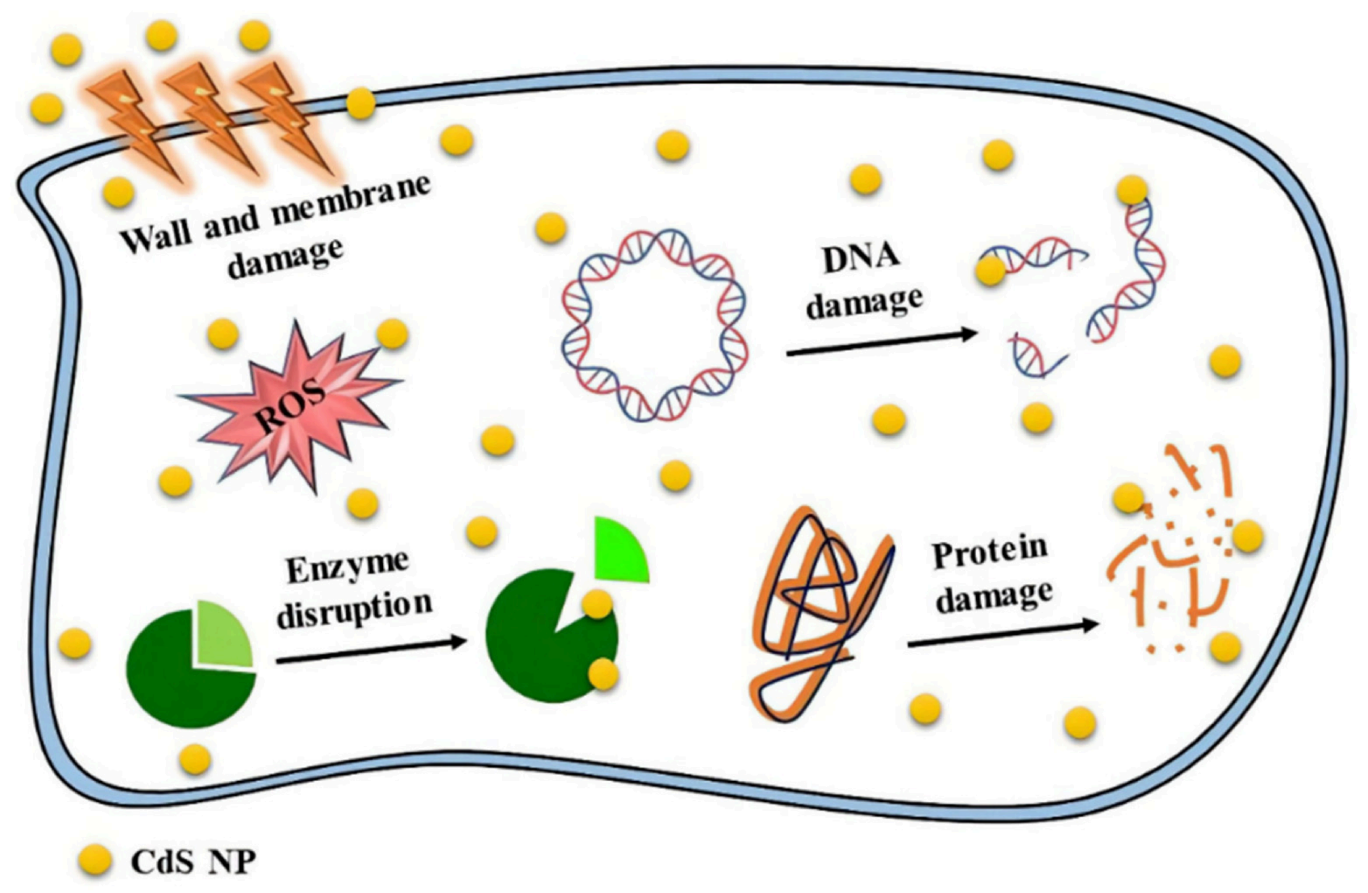
A schematic illustration of the mechanisms of action of the antimicrobial activities of CdS. Reprinted with permission from [54] (CC BY license).

CdS exhibit a multifaceted antibacterial mechanism that involves photocatalytic ROS generation, controlled release of metal ions, membrane disruption, and interference with bacterial metabolic pathways [54,55,56]. These mechanisms operate synergistically and are significantly enhanced when CdS is integrated with other functional materials to form heterojunction nanocomposites. Such multimodal activity enables CdS-based systems to effectively inactivate a broad spectrum of microorganisms, including antibiotic-resistant bacterial strains (Figure 5).

One of the primary antibacterial pathways of CdS arises from its photocatalytic activity under visible-light irradiation. CdS is a narrow-bandgap semiconductor (≈2.4 eV) that absorbs visible light and generates electron–hole pairs. Upon illumination, electrons are excited from the valence band to the conduction band, leaving holes behind. These photogenerated charge carriers participate in redox reactions with molecular oxygen and water, producing ROS such as •O_2_^−^, •OH, and ^1^O_2_. These ROS induce oxidative stress within microbial cells by attacking membrane lipids, denaturing proteins, and damaging nucleic acids [55,56,57,58,59,60]. Lipid peroxidation compromises membrane integrity, while oxidative modification of enzymes and ribosomal proteins disrupts cellular metabolism and protein synthesis, ultimately resulting in bacterial cell death.

A critical evaluation of the mechanistic evidence reveals that many studies infer antibacterial mechanisms indirectly rather than proving them experimentally. The most robust evidence for ROS involvement comes from electron spin resonance (ESR) spectroscopy coupled with spin-trapping agents such as DMPO, which enables unambiguous identification of radical species generated during photocatalysis [56,57,58]. For CdS systems, ESR studies have confirmed the generation of superoxide radicals (•O_2_^−^), hydroxyl radicals (•OH), and carbon-based radicals upon visible-light irradiation. Scavenger experiments using specific quenchers, such as EDTA-2Na for holes (h^+^), benzoquinone for superoxide radicals (•O_2_^−^), and isopropyl alcohol for hydroxyl radicals (•OH), provide complementary evidence for the relative contribution of different ROS [55,56,57,58,59,60]. For instance, trapping experiments on rGO/CdS QD heterostructures demonstrated that hydroxyl radicals were the primary reactive species responsible for photodegradation (discussed in Section 7 and Section 10). Similarly, fluorescence probing using terephthalic acid (TPA) has been employed to detect hydroxyl radical generation through the formation of fluorescent 2-hydroxyterephthalic acid. This TPA-based method, which converts non-fluorescent terephthalic acid into highly fluorescent 2-hydroxyterephthalic acid upon reaction with •OH radicals, has been widely used in semiconductor photocatalysis to quantify hydroxyl radical production and evaluate charge transfer efficiency [57,58,59,60,61,62].

The relative contribution of different ROS varies depending on the CdS architecture and reaction conditions. CdS predominantly generates superoxide radicals (•O_2_^−^) and singlet oxygen (^1^O_2_) under illumination, while hydroxyl radical (•OH) formation is less favored due to the valence band potential of CdS (approximately +1.8 V vs. NHE) being insufficient for direct water oxidation to •OH (E^0^ = +2.4 V). However, •OH can still be generated through sequential reduction of oxygen to hydrogen peroxide followed by Fenton-type reactions or through surface-mediated processes. In heterojunction systems with favorable band alignment, such as S-scheme g-C_3_N_4_/CdS, enhanced ROS generation including •OH becomes achievable due to the preserved strong oxidation potential. Studies on sulfide-based photocatalysts have demonstrated that charge transfer processes at heterojunction interfaces are critically important in determining photocatalytic performance, as efficient interfacial charge separation suppresses electron–hole recombination and promotes ROS generation [56,57,58,59,60,61,62,63].

The distinction between photocatalytic toxicity and dark toxicity is critical for understanding the true antibacterial mechanism. Several studies have reported that CdS nanomaterials exhibit limited or negligible antibacterial activity in the absence of light, with significant bacterial inactivation only observed upon illumination. For example, CdS/MCM-41/HNTs composites showed no pronounced activity in the dark even at high concentrations (1000 µg/mL), whereas under visible-light irradiation even comparatively small concentrations (125 µg/mL) achieved 100% bacterial growth inhibition. This light-dependent behavior confirms that ROS generation, rather than simple Cd^2+^ release, is the dominant antibacterial pathway in most CdS-based systems. However, some architectures, particularly those with a high surface area or unprotected surfaces, do exhibit dark toxicity primarily through Cd^2+^ ion release, which can bind to sulfhydryl groups of membrane proteins, disrupt enzymatic activity, and induce oxidative stress [59,60,61,62].

The contribution of dissolved Cd^2+^ ions versus ROS-mediated damage has been a subject of debate in the literature. While Cd^2+^ release is often observed, particularly under acidic conditions where photocorrosion is accelerated, several studies have demonstrated that the primary bactericidal mechanism is ROS-driven rather than ion toxicity. In a mechanistic study of CdS/C_60_ composites, researchers employed ICP technology to quantify Cd^2+^ release and performed control experiments with Cd^2+^ solutions at equivalent concentrations. The results confirmed that the antibacterial effect was primarily attributed to ROS produced during photocatalysis, which destroy cell membrane integrity and further damage intracellular DNA, rather than Cd^2+^ toxicity. Similarly, comparative toxicity studies of CdS, MoS_2_, and WS_2_ nanoparticles under UV irradiation revealed that ROS generation and toxic ion release jointly contributed to the antibacterial activity of CdS, with ROS playing a dominant role [56,57,58,59,60].

Distinguishing photocatalytic effects from photothermal effects requires careful experimental design. While some CdS-based composites incorporating noble metals (e.g., Au, Ag) or carbonaceous materials may exhibit localized heating under irradiation, the antibacterial activity of pristine CdS and most heterojunctions is primarily photocatalytic rather than photothermal. The relatively small bandgap of CdS (2.4 eV) enables efficient visible-light absorption for charge separation, but the photothermal conversion efficiency is generally low compared to materials with strong plasmonic or near-infrared absorption. Studies that report photothermal contributions typically involve composite systems with additional photothermal agents, and the mechanism should be confirmed through temperature monitoring and control experiments under identical illumination conditions.

The photocatalytic efficiency of CdS can be significantly improved through the formation of semiconductor heterojunctions or conductive composites. For instance, CdS combined with wide-bandgap semiconductors such as TiO_2_ or ZnO forms type II heterojunctions that promote spatial separation of photogenerated electrons and holes, reducing charge recombination and prolonging carrier lifetimes [57,58,59,60,61]. Similarly, the incorporation of conductive materials such as graphene or carbon-based nanostructures can create Schottky barriers that act as electron sinks, further suppressing recombination processes. These structural modifications enhance ROS production and significantly improve antibacterial performance compared with pristine CdS photocatalysts.

In addition to photocatalytic ROS generation, CdS can exert antibacterial effects through the release of Cd^2+^ ions. These ions interact strongly with sulfur-containing and nitrogen-containing functional groups present in bacterial proteins and enzymes. Binding of Cd^2+^ to thiol (–SH) groups can deactivate essential metabolic enzymes and interfere with respiratory pathways [59,60,61,62]. Furthermore, Cd^2+^ ions may penetrate the bacterial cytoplasm and interact with nucleic acids, leading to structural alterations that inhibit DNA replication and transcription. The combination of ion toxicity and oxidative stress amplifies the bactericidal effect of CdS-based nanomaterials.

Direct interactions between CdS and bacterial membranes also contribute to antimicrobial activity. Due to their high surface area and surface charge, CdS nanostructures can adhere electrostatically to negatively charged bacterial cell membranes. This interaction destabilizes membrane structures and increases permeability, causing leakage of intracellular components such as proteins, ions, and nucleotides [58,59,60,61,62,63]. Structural damage to membrane proteins and porin channels further disrupts nutrient transport and cellular homeostasis, accelerating bacterial inactivation even under dark conditions.

Recent studies have also demonstrated that CdS can exhibit additional antibacterial pathways when integrated with multifunctional materials. For example, CdS-based heterostructures coupled with noble metals or carbon materials may generate localized photothermal effects under near-infrared irradiation, leading to temperature increases that destabilize bacterial membranes and denature cellular proteins [62,63,64,65,66]. Similarly, magnetic or catalytic components incorporated into CdS can promote chemodynamic reactions that continuously generate reactive radicals, providing antibacterial activity even in the absence of light.

The convergence of these mechanisms, ROS-mediated oxidative stress, metal ion toxicity, membrane disruption, and photothermal or chemodynamic effects, creates a powerful multimodal antibacterial platform. Such synergistic interactions enable CdS to overcome bacterial defense mechanisms such as efflux pumps, antioxidant enzymes, and biofilm formation. As a result, CdS-based materials demonstrate strong potential for combating multidrug-resistant pathogens.

Despite these advantages, certain challenges remain in the application of CdS nanomaterials, particularly related to photocorrosion and potential cadmium ion toxicity. Strategies such as surface coating, core–shell structures, polymer encapsulation, and heterojunction engineering have been explored to stabilize CdS and control ion release while maintaining high antibacterial activity. Continued research into these design strategies will be essential for optimizing CdS as efficient and safe antibacterial agents for biomedical and environmental applications.

## 6. Antibacterial Performance of CdS-Based Pristine Materials

The antibacterial activity of pristine CdS is governed by a complex interplay of physicochemical parameters, including crystallite size, morphology, surface defects, synthesis route, and surface functionalization. Unlike composite systems where charge transfer across heterointerfaces dominates, pristine CdS relies entirely on its intrinsic photocatalytic ROS generation, controlled Cd^2+^ ion release, and direct membrane interaction. Consequently, optimizing synthesis conditions including temperature, precursor ratios, capping agents, and solvent systems, is important for maximizing antimicrobial efficacy while minimizing cytotoxicity. This section systematically evaluates the antibacterial performance of standalone CdS, ranging from quantum dots (2–10 nm) to hierarchical architectures, as reported in representative studies over the past decade. Particular attention is paid to how synthesis parameters influence the minimum inhibitory concentration (MIC), zone of inhibition (ZOI), and bacterial selectivity between Gram-positive and Gram-negative pathogens. Table 2 provides a comprehensive comparative summary of these pristine systems.

Based on the work of Singh et al. [67], the antibacterial and antifungal efficacy of CdS quantum dots (QDs) was systematically optimized by controlling synthesis parameters (Table 2). The study revealed that QDs synthesized at 50 °C with a 1:0.5 Cd:S ratio and surface modification using PEG and PVPP exhibited the most potent antimicrobial activity. Against *E. coli*, these QDs achieved ZOI values of up to 25.5 mm at 10 mg/mL (CdS concentration), while against *S. aureus*, the ZOI reached 19.5 mm under similar conditions. The QDs were also highly effective against the fungus *A. flavus*, with a ZOI of 20.0 mm at just 5 mg/mL (CdS concentration). The authors attributed this enhanced performance to the smaller particle size achieved at lower synthesis temperatures and the half Cd:S ratio, which increased the surface-area-to-volume ratio and promoted greater ROS generation. The surfactants PEG and PVPP played a crucial dual role: they stabilized the QDs, reducing cytotoxicity without compromising optical properties, while also enhancing interaction with microbial membranes. Gram-negative *E. coli* was more susceptible than Gram-positive *S. aureus* due to its thinner peptidoglycan layer. These findings underscore the importance of precise parameter control in engineering CdS for antimicrobial applications at record-low concentrations.

Mostafa et al. [68] successfully synthesized CdS as nanoparticles using one-pot green pulsed laser ablation in liquid media. In this method, a cadmium metal target was ablated in DMSO, which served as a non-toxic sulfur source. The resulting CdS exhibited a hexagonal crystalline structure with an average size of approximately 45–50 nm, as confirmed by XRD and TEM analysis. The antimicrobial efficacy of the synthesized CdS was evaluated against a panel of pathogens, including *Bacillus subtilis*, *Staphylococcus aureus*, *Escherichia coli*, *Salmonella* sp., and the fungus Candida albicans. While the CdS demonstrated clear antimicrobial activity in diffusion plate assays, their MIC was determined to be 6.25 µg/mL against the tested microorganisms, which was notably higher (less potent) than the 0.78 µg/mL MIC recorded for CuS nanoparticles also synthesized in the study. The paper posits that the antimicrobial mechanism of CdS is primarily limited to its ability to penetrate the cell membrane and disrupt replication, a more constrained mode of action compared to the multifaceted oxidative and membrane-damaging effects observed with copper-based nanoparticles. The study did not investigate the addition of any additives to the CdS to improve its performance, focusing instead on the comparative analysis of the different metal sulfides synthesized via the same method of pulsed laser ablation in liquid media.

Munishwar et al. [69] investigated the antibacterial activity of highly stable CdS embedded within a borosilicate glass matrix, synthesized via a melt–quench technique followed by controlled heat treatment. The growth of spherical CdS with an average size of approximately 3.85 nm was confirmed through HRTEM, XRD, and XPS analyses, and their quantum confinement effect was evidenced by a blueshift in the optical bandgap. The study evaluated the antibacterial efficacy of the QD-embedded glass powder against Gram-positive *Staphylococcus aureus* and Gram-negative *Escherichia coli* by determining the MIC required to inhibit 50% of the bacterial isolates (MIC50). The results demonstrated that the CdS exhibited concentration-dependent antibacterial activity under light conditions, with MIC50 values of 61.36 μg/mL for *S. aureus* and 88.07 μg/mL for *E. coli*, indicating greater susceptibility of the Gram-positive bacterium. The authors attributed the antibacterial mechanism to the photocatalytic generation of ROS upon light absorption by the CdS. The photogenerated electron–hole pairs react with oxygen and water to produce O_2_^−^, OH•, and ultimately H_2_O_2_. While negatively charged radicals remain on the bacterial surface, H_2_O_2_ can penetrate the cell membrane, causing oxidative damage and inhibiting growth. The thicker peptidoglycan layer of Gram-positive bacteria offers less protection against H_2_O_2_ compared to the complex outer membrane of Gram-negative bacteria, explaining the observed difference in MIC50 values. The embedding of CdS in the stable glass matrix effectively prevents photocorrosion and enhances their reusability as a solid antimicrobial agent, positioning this composite as a promising candidate for water treatment and pathogen inhibition applications.

Nasrin et al. [70] investigated the growth inhibitory mechanism of CdS as nanoparticles, biosynthesized using the fungus Aspergillus foetidus, against the model bacterium *Escherichia coli* K12. The biogenic route yielded spherical, polydispersed CdS with an average size of approximately 15 nm. The study established that the antibacterial effect is dose-dependent, with the MIC determined at 200 μL of the nanoparticle suspension, beyond which a drastic reduction in colony-forming units (from 10^9^ to 10^4^ CFU/mL) was observed. The authors elucidated that the primary mechanism of growth inhibition is not merely due to the presence of the particles but is mediated through the induction of significant oxidative stress. Treatment with 200 μL of CdS resulted in a substantial increase in intracellular ROS, with flow cytometry analysis showing that 46.82% of the treated cells were ROS-positive compared to only 0.53% in the control. This surge in ROS, quantified as an approximate 50% increase via fluorometric analysis, led to severe oxidative damage to the bacterial cell membrane. Specifically, the generated ROS caused a five-fold increase in lipid peroxidation, as measured by the malondialdehyde byproduct in the thiobarbituric acid assay. The consequence of this membrane damage was further evidenced by morphological changes, including cell filamentation up to 10 μm in length at sub-inhibitory concentrations, which ultimately culminated in cell lysis and death at the MIC. The work concludes that the A. foetidus-mediated CdS exert their antimicrobial effect through a reactive oxygen species-dependent pathway that leads to membrane lipid peroxidation, offering a mechanistic understanding of their potential for applications where controlling *E. coli* growth is essential.

Alsaggaf et al. [71] successfully biosynthesized stable, polydispersed CdS as nanoparticles using a green approach with the cadmium-tolerant fungus *Aspergillus niger* (RCMB 002002). The process involved challenging the fungal biomass with aqueous cadmium chloride and sodium sulfide, resulting in the formation of spherical nanoparticles with a size range of 2–10 nm, as confirmed by TEM and DLS analysis, with 100% of the particles measuring between 2.7 and 7.5 nm. Structural and optical characterization via XRD, FTIR, and photoluminescence spectroscopy confirmed the cubic phase of the CdS and their stabilization by fungal proteins, which acted as capping agents. The biosynthesized CdS exhibited significant antibacterial activity against both Gram-positive and Gram-negative bacteria, including *Bacillus subtilis*, *Staphylococcus aureus*, *Escherichia coli*, and *Proteus vulgaris*, with inhibition zones ranging from 14 to 30 mm. Notably, the nanoparticles showed greater efficacy against Gram-positive bacteria, attributed to the absence of an outer membrane and the presence of multiple peptidoglycan layers with negatively charged teichoic acids that facilitate interaction with cadmium ions. No antifungal activity was detected against Candida albicans. In addition to their antimicrobial properties, the CdS demonstrated potent in vitro cytotoxic activity against human cancer cell lines, with 50% inhibitory concentrations (IC50) of 190 μg/mL against breast cancer (MCF7), 246 μg/mL against prostate cancer (PC3), and 149 μg/mL against lung cancer (A549) cells. The proposed mechanism of anticancer activity involves the release of toxic Cd^2+^ ions from the nanoparticle surface under oxidative conditions and the generation of ROS, leading to cell death. This study highlights the potential of *A. niger*-mediated CdS as a multifunctional nanomaterial for both antimicrobial and anticancer biotechnological applications.

Alkhayatt and Ahmed [72] synthesized CdS as nanoparticles via a hydrothermal method at varying reaction temperatures (140, 160, 180, and 200 °C) and systematically investigated the effect of temperature on their structural, morphological, and antibacterial properties. XRD analysis confirmed the formation of highly crystalline, polycrystalline nanoparticles with a hexagonal wurtzite phase, with the average crystallite size increasing from approximately 21 to 24 nm as the reaction temperature rose. FESEM revealed that the nanoparticles exhibited a distinctive rounded “cauliflower-like” spherical morphology, with particle sizes ranging from 23 to 245 nm, where the smallest particle size was observed at the highest reaction temperature of 200 °C. The antibacterial efficacy of the synthesized CdS was evaluated against Gram-negative *Escherichia coli* and Gram-positive *Staphylococcus aureus* using the agar well diffusion method at concentrations of 100, 250, 500, and 1000 μg/mL. The results demonstrated a strong, concentration-dependent antibacterial effect. The highest antibacterial activity was achieved with nanoparticles prepared at 160 °C and 180 °C, producing maximum inhibition zones of 30 mm against *E. coli* and 19 mm against *S. aureus* at the highest concentration (1000 μg/mL). Notably, *E. coli* exhibited greater susceptibility to the CdS than *S. aureus*. The proposed antibacterial mechanism involves the adherence of CdS to the bacterial cell wall, leading to membrane damage and leakage of cellular contents. Furthermore, the nanoparticles can penetrate the cells, where they and their released ions (Cd^2+^) interact with and disrupt essential biomolecules such as proteins, enzymes, lipids, and DNA, ultimately impairing cellular respiration and causing cell death. This study underscores the potential of hydrothermally synthesized CdS, particularly those produced at optimized temperatures, as effective antibacterial agents for potential applications in nanomedicine.

Alpdoğan et al. [73] fabricated CdS as thin films on glass substrates via CBD at 70 °C with varying deposition times (2–5 h) and systematically investigated the effects of bacterial adhesion on the films’ structural, optical, and electrical properties. The as-deposited CdS thin films exhibited uniform spherical nano-sized grains with a polycrystalline structure composed of mixed hexagonal and cubic phases, an optical bandgap ranging from 2.37 to 2.42 eV, and a characteristic electrical resistivity on the order of 10^5^ Ω·cm with n-type conductivity. To assess bacterial interaction, the 4 h deposited films were incubated for 10 days with four biofilm-forming bacterial strains: *Bacillus subtilis*, *Klebsiella pneumoniae*, *Pseudomonas aeruginosa*, and *Staphylococcus aureus*. SEM imaging revealed that adhesion propensity was highly strain-dependent; *S. aureus* exhibited the greatest attachment and colonization, followed by *K. pneumoniae*, while *P. aeruginosa* showed minimal adhesion and *B. subtilis* cells were scarcely detected on the film surface. Crucially, despite the varying degrees of bacterial attachment and biofilm formation, the core functional properties of the CdS as thin films remained unaltered. The optical bandgap remained constant at 2.42 eV, and XRD analysis confirmed that neither the crystal structure, peak intensities, nor the calculated crystallite size and dislocation density were affected by bacterial interaction. Furthermore, the electrical resistivity of the films (~4 × 10^5^ Ω·cm) was unchanged, indicating that bacterial adhesion does not induce defects or disrupt the semiconductor’s performance. In parallel, disk diffusion tests evaluating the antimicrobial effect of the CdS themselves showed varying inhibition zones against the tested bacteria, with the highest antimicrobial effect observed against *B. subtilis* and the lowest against *S. aureus*. This study demonstrates that while bacterial adhesion to CdS as thin film surfaces varies by strain and can lead to colonization, it does not degrade the films’ critical optoelectronic properties, underscoring their potential stability in microbially rich environments for technological applications.

Burashev et al. [74] developed a novel, environmentally friendly mechanochemical method for synthesizing CdS (m-CdS) nanoparticles using non-toxic precursors, including thiourea as a sulfide ion source, in a planetary ball mill at room temperature. For comparison, CdS were also prepared via a conventional solvothermal method (s-CdS). Comprehensive characterization using XRD, Raman spectroscopy, XPS, SEM, and TEM revealed that m-CdS consisted of spherical nanoparticles with a diameter of 5–6 nm, approximately 10 times smaller than the s-CdS nanorods, and exhibited a mesoporous structure with a high specific surface area (126.98 m^2^/g) compared to s-CdS (49.31 m^2^/g). The photocatalytic activity of m-CdS was superior to that of s-CdS, achieving nearly 100% degradation of Orange II dye under visible light within 90 min, with a rate constant (0.0377 min^−1^) approximately 5.5 times higher than that of s-CdS (0.0068 min^−1^). Additionally, m-CdS decorated with NiS as a cocatalyst exhibited a maximum hydrogen evolution rate of 191.9 μmol g^−1^ h^−1^, over three times higher than that of s-CdS/NiS (58.0 μmol g^−1^ h^−1^). In antibacterial assays, m-CdS demonstrated potent and broad-spectrum activity against a panel of Gram-positive and Gram-negative bacteria, while s-CdS showed no antibacterial effect. The minimum bactericidal concentration (MBC) and minimum inhibitory concentration (MIC) for m-CdS against *Staphylococcus aureus* ATCC 6538-P were 0.04 mg/mL and 0.02 mg/mL, respectively, and it produced large inhibition zones, including 33.0 mm against *S. aureus*—exceeding the 25.7 mm zone of the reference antibiotic amoxicillin. The superior performance of m-CdS was attributed to its smaller particle size, higher surface area, and greater surface reactivity, which enhance charge separation, ROS generation, and interaction with microbial cells. This study highlights the mechanochemical approach as a rapid (20 min), solvent-free, and scalable green technology for producing highly multifunctional CdS with excellent photocatalytic and antibacterial properties for environmental and biomedical applications.

Ayodhya et al. [75] successfully synthesized CdS as nanoparticles capped with a Schiff base (SB), 2-[(4-methoxy-phenylimino)-methyl]-4-nitrophenol, using a simple and efficient sonochemical method. The SB served as a complexing and capping agent to control nanoparticle growth and morphology. Comprehensive characterization confirmed the formation of nearly spherical, monodisperse CdS as nanoparticles with a cubic crystal structure and an average particle size of 2–10 nm, as determined by TEM and XRD. UV-vis diffuse reflectance spectroscopy revealed a blue shift in the absorption edge (from 590 nm to 570 nm for SB-capped CdS), corresponding to a bandgap of 2.17 eV, indicative of quantum confinement effects. FTIR spectroscopy confirmed the interaction between CdS and the SB through Cd–O, Cd–N, and Cd–S bonds, while zeta potential measurements (+2.98 mV for capped vs. −2.04 mV for uncapped) indicated enhanced stability due to surface modification. Fluorescence studies showed that the SB effectively quenched the fluorescence of CdS as nanoparticle in a concentration-dependent manner, with a Stern-Volmer quenching constant (K_SV) of 0.12 × 10^5^ L mol^−1^, suggesting static quenching via strong interactions between the SB and the nanoparticle surface. The photocatalytic activity of the SB-capped CdS NPs was evaluated through the degradation of methyl red dye under sunlight irradiation. The nanoparticles exhibited efficient, concentration-dependent photocatalytic performance, with 30 mg of catalyst achieving 82.59% degradation within 4 h, following pseudo-first-order kinetics (rate constant k = 0.431 min^−1^). The degradation mechanism was elucidated via ESI-MS analysis, revealing the formation of intermediates such as 4,4-N,N-dimethyl aniline and 2-hydroxy benzoic acid, which ultimately mineralized to simple aliphatic acids and CO_2_. The antibacterial activity of the synthesized materials was assessed against Gram-negative *Escherichia coli* and *Pseudomonas aeruginosa*, and Gram-positive *Staphylococcus aureus* using the disk diffusion method. The SB-capped CdS exhibited significantly enhanced antibacterial activity compared to uncapped CdS and the free SB alone, with inhibition zones of 18.04 mm (*E. coli*), 21.31 mm (*S. aureus*), and 25.62 mm (*P. aeruginosa*) at a concentration of 1 mg/mL. This enhanced activity was attributed to the chelation effect, where coordination of the SB with Cd^2+^ reduces the polarity of the metal ion, increasing the lipophilic character and facilitating permeation through the bacterial lipid membrane, leading to cell damage and growth inhibition. This study demonstrates that SB-capped CdS are promising multifunctional nanomaterials for fluorescence-based sensing, photocatalytic wastewater treatment, and antibacterial applications.

Faisal et al. [76] successfully synthesized CdS as nanoparticles using a green, eco-friendly approach, where an ethanolic extract of the plant *Lathyrus aphaca* L. (CdS-LA) served as both a reducing and capping agent. The nanoparticles were formed by reacting cadmium acetate with sodium sulfide in the presence of the plant extract, which facilitated surface modification and stabilization. Comprehensive characterization confirmed the successful synthesis of the hybrid nanomaterial: XRD revealed a predominantly hexagonal crystal structure with an average crystallite size of approximately 58 nm; SEM showed a homogeneous surface morphology with spherical nanoparticles; EDX confirmed the presence of cadmium and sulfur along with carbon and oxygen, indicating the attachment of phytochemicals to the nanoparticle surface; and FTIR verified Cd-S bond formation alongside functional groups characteristic of plant-derived compounds, such as hydroxyl and C-N groups. UV–visible spectroscopy displayed an absorption peak at 543 nm, with a blue shift attributed to quantum confinement effects. The antimicrobial efficacy of the CdS-LA hybrid nanoparticles was evaluated against a panel of five bacterial strains (*Bacillus subtilis*, *Staphylococcus aureus*, *Streptococcus* species, *Escherichia coli*, and *Klebsiella pneumoniae*) and five fungal strains (*Aspergillus flavus*, *Alternaria alternata*, *Collatorichum dematium*, *Fusarium roseum*, and *Curvularia lunata*) using the well diffusion method. The nanoparticles exhibited concentration-dependent antimicrobial activity, with the highest concentration tested (1000 μg/mL) producing significant inhibition zones. Against bacteria, the greatest effect was observed against *Streptococcus* species (31.06 mm) and *Staphylococcus aureus* (28.82 mm), while *Klebsiella pneumoniae* showed the least susceptibility (10.2 mm). In antifungal assays, the nanoparticles demonstrated potent activity, particularly against *Collatorichum dematium*, achieving 77.8% inhibition relative to the standard Fluconazole. The proposed mechanism of action involves the generation of ROS leading to oxidative stress, direct physical disruption of microbial membranes due to the nanoscale size and high surface area, and potential synergistic effects from the bioactive phytochemicals (flavonoids, alkaloids, phenolic compounds) present on the nanoparticle surface, which may interfere with microbial metabolic pathways. This study highlights the potential of *L. aphaca*-capped CdS as versatile antimicrobial agents for biomedical and agricultural applications.

**Figure 6:**
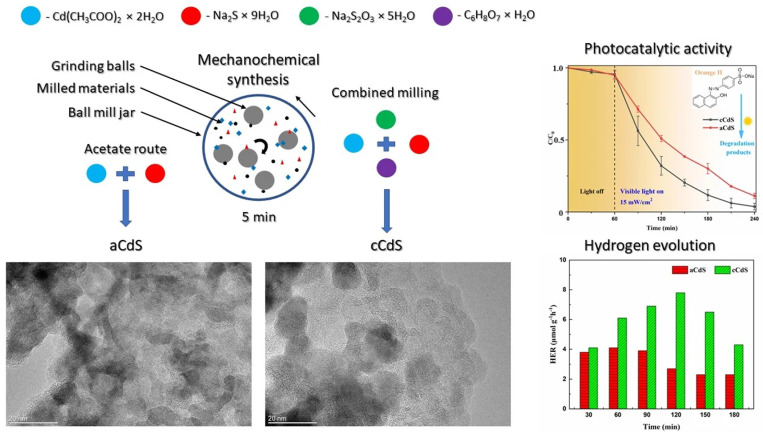
A schematic illustration of the synthesis, TEM image, photocatalytic activity, and hydrogen evolution of CdS. Reprinted with permission from [77] (CC BY license).

Shalabayev et al. [77] successfully synthesized CdS as nanoparticles using two solvent-free, environmentally friendly mechanochemical protocols: a conventional acetate route (aCdS) using cadmium acetate and sodium sulfide, and a novel combined approach (cCdS) incorporating sodium thiosulfate as an additional sulfur source alongside citric acid (Figure 6). Both methods yielded nanocrystalline CdS with a predominant cubic phase, as confirmed by XRD and TEM, though the cCdS sample exhibited smaller crystallites, a higher bandgap (2.78 eV vs. 2.36 eV for aCdS), and unique surface defects that enhanced charge separation. The photocatalytic performance of the nanoparticles was evaluated through the degradation of Orange II dye and hydrogen evolution under visible light. The cCdS demonstrated superior activity, achieving 93% dye degradation within 180 min with a rate constant 1.5 times higher than aCdS (0.018 min^−1^ vs. 0.012 min^−1^), and a peak hydrogen evolution rate of 7.8 μmol g^−1^ h^−1^, nearly double that of aCdS (4.1 μmol g^−1^ h^−1^). The enhanced photocatalytic activity of cCdS was attributed to its larger bandgap, which promotes stronger oxidizing and reducing properties, and its higher density of crystal defects, which facilitate electron–hole separation. The antibacterial potential of both CdS samples was assessed against reference strains of *Escherichia coli* (Gram-negative) and *Staphylococcus aureus* (Gram-positive) through 24 h toxicity tests, disk diffusion assays, and respiration monitoring during the logarithmic growth phase. While neither nanomaterial produced clear inhibition zones in disk diffusion tests, indicating minimal ion release, respiration monitoring revealed that both CdS could inhibit bacterial growth during the logarithmic phase in a concentration-dependent manner. Notably, the aCdS exhibited stronger growth inhibition than cCdS, likely due to their smaller grain size distribution, which enhances nanoparticle-cell interactions and toxicity. This study highlights the versatility of mechanochemical synthesis for producing CdS with tunable properties, where the combined milling approach (cCdS) is more favorable for photocatalytic applications due to enhanced charge dynamics, while the acetate route (aCdS) shows greater promise for antibacterial applications owing to its smaller particle size and higher surface reactivity.

Dadmehr et al. [78] developed an eco-friendly, green synthesis approach for producing CdS as QDs using an aqueous extract of saffron (*Crocus sativus* L.) stigma, which served as both a reducing and capping agent. The synthesis was optimized at different pH conditions (3, 7, and 11), with the basic condition (pH 11) yielding the most favorable optical properties, including the lowest energy bandgap of 2.4 eV, as determined by UV-Vis spectroscopy and Tauc plot analysis. The biosynthesized CdS were characterized by TEM, which revealed well-dispersed, spherical nanoparticles with a uniform size distribution ranging from 2 to 6 nm and a mean diameter of 4.1 nm. FTIR spectroscopy confirmed the presence of phytochemicals such as polyphenols and peptides on the QD surface, which contributed to their stabilization and functionalization. The photocatalytic activity of the CdS was evaluated through the degradation of Rhodamine B (RhB) dye under UV light irradiation, achieving 92% degradation within 80 min, with a pseudo-first-order rate constant of 0.029 min^−1^. The mechanism was attributed to the generation of electron–hole pairs upon light excitation, leading to the formation of reactive oxygen species (ROS) such as superoxide radicals (O_2_^−^) and hydroxyl radicals (OH•), which are responsible for dye breakdown. In antibacterial assays using the disk diffusion method, the CdS exhibited significant activity against both Gram-positive *Bacillus subtilis* and Gram-negative *Escherichia coli* at a concentration of 50 μg/mL, producing inhibition zones of 12.5 mm and 14.5 mm, respectively, with percentage inhibitions of 90% and 96%. These values were comparable to or better than the standard antibiotic streptomycin. Furthermore, the CdS demonstrated potent cytotoxicity against MCF-7 breast cancer cells in a dose-dependent manner, as determined by MTT assay, with the highest concentration (100 μg/mL) inducing approximately 79% cell death. The cytotoxic effect was attributed to the small size of the QDs facilitating cellular uptake and the photocatalytic generation of ROS under light, leading to oxidative stress and apoptosis. This study highlights the potential of saffron-mediated CdS as a multifunctional nanomaterial for photocatalytic water treatment, antibacterial agents, and targeted cancer therapy.

Rajeshkumar et al. [79] successfully demonstrated an eco-friendly, extracellular synthesis of CdS as nanoparticles using the marine bacterium *Enterococcus* sp. (RMAA). The synthesis was achieved by reacting the bacterial culture supernatant with CdSO_4_, where secreted biomolecules, likely enzymes such as sulfate reductase, facilitated the reduction of Cd^2+^ ions and formation of CdS. The process was visually confirmed by a color change from yellow to white and monitored by UV-Vis spectroscopy, which showed a characteristic surface plasmon resonance peak at 410 nm. Comprehensive characterization confirmed the successful formation of stable, spherical CdS with a crystalline cubic structure, as evidenced by XRD, and particle sizes ranging from 50 to 180 nm, as observed by SEM. FTIR analysis indicated the presence of protein capping on the nanoparticle surface, with amide linkages suggesting that biomolecules from the bacteria act as stabilizing agents, preventing agglomeration and enhancing long-term stability (over two months). The antimicrobial efficacy of the biosynthesized CdS was evaluated against a panel of pathogenic bacteria (*Serratia nematodiphila*, *Escherichia coli*, *Klebsiella planticola*, *Vibrio* sp., and *Planomicrobium* sp.) and fungi (*Aspergillus niger* and *Aspergillus flavus*) using the agar well diffusion method. The nanoparticles exhibited dose-dependent antimicrobial activity, with inhibition zones increasing with concentration (50, 100, and 200 μL). The highest antibacterial activity was observed against *K. planticola* (23.27 mm), *Planomicrobium* sp. (22.14 mm), and *Vibrio* sp. (21.28 mm) at 200 μL, while moderate activity was recorded against *S. nematodiphila* (14.27 mm) and *E. coli* (10.23 mm). In antifungal assays, the CdS showed significant activity, with maximum inhibition zones of 19.21 mm against *A. flavus* and 16.24 mm against *A. niger* at the highest concentration. The proposed mechanism of antimicrobial action involves electrostatic attraction between positively charged nanoparticles and negatively charged microbial cell membranes, leading to membrane disruption, protein denaturation, and ultimately cell death. This study highlights the potential of marine bacteria as a sustainable, green platform for the synthesis of stable, bioactive CdS with promising applications in controlling pathogenic microorganisms.

Ali et al. [80] successfully synthesized CdS as nanoparticles via a hydrothermal method using cadmium nitrate and sodium sulfide as precursors, and comprehensively characterized the material for its structural, optical, thermal, and biological properties. UV-Vis spectroscopy revealed a characteristic absorption peak at 496 nm, and the optical bandgap was determined to be 2.38 eV via Tauc plot, indicating its suitability for visible-light-driven photocatalysis. XRD confirmed the formation of highly crystalline, hexagonal-phase CdS, while SEM showed aggregated clusters of nanoparticles. EDX analysis confirmed the presence of Cd and S as the major elements, and FTIR spectroscopy verified Cd-S bond formation along with minor organic impurities. TGA demonstrated excellent thermal stability, with a total weight loss of only 6.54% up to 600 °C. The photocatalytic activity of the CdS was evaluated for the degradation of two organic dyes, Eosin B (EB) and Methyl Green (MG), under UV light irradiation. The degradation followed pseudo-second-order kinetics, with rate constants increasing with temperature. The calculated activation energies were 61.1 kJ/mol for EB and 32.11 kJ/mol for MG. Optimal degradation conditions were established—a catalyst dose of 0.03 g/L, a low initial dye concentration (10 ppm), and a temperature of 45 °C—achieving 95% degradation for EB and 90% for MG within 160 min. The degradation efficiency was strongly pH-dependent, with EB (cationic) showing maximum degradation at high pH (pH 10) due to electrostatic attraction with negatively charged catalyst surfaces, while MG (anionic) degraded most efficiently at low pH (pH 4). The catalyst exhibited good reusability, retaining >64% degradation efficiency after five cycles. The antibacterial activity of the CdS was assessed against pathogenic bacteria (*Staphylococcus aureus*, *Acinetobacter baumannii*, and *Escherichia coli*) using the agar well diffusion method. The nanoparticles showed good antibacterial activity, with the highest inhibition zone observed against *S. aureus* (approximately 22 mm at 100 μL), attributed to the small particle size facilitating membrane penetration and interaction with thiol-containing enzymes. Biocompatibility was evaluated via a human red blood cell (RBC) hemolysis assay, which revealed concentration-dependent hemolytic activity. This study highlights the multifunctional potential of hydrothermally synthesized CdS as an effective photocatalyst for wastewater treatment and as an antibacterial agent, while also underscoring the need for careful toxicity evaluation in biological applications.

Omar et al. [81] systematically investigated the influence of synthesis method and calcination temperature on the structural, morphological, and antimicrobial properties of CdS nanoparticles. CdS was synthesized via two distinct routes, co-precipitation (samples BA1–BA3) and solvothermal (samples BA4–BA6), followed by calcination at 100, 200, and 300 °C. XRD analysis confirmed that all samples crystallized in the hexagonal wurtzite phase with high phase purity, and crystallite sizes ranged from 11.9 to 41.3 nm, with solvothermally synthesized nanoparticles exhibiting larger crystallite sizes and enhanced crystallinity due to the elevated pressure and temperature conditions in the autoclave. SEM coupled with EDS revealed homogeneous distribution of cadmium and sulfur, confirming successful nanoparticle formation, with minor surface oxidation observed. The antimicrobial efficacy of the nanoparticles was rigorously evaluated against Gram-positive *Bacillus cereus* (ATCC 14579) and Gram-negative *Escherichia coli* (ATCC 25322) through disk diffusion, MIC, minimum bactericidal concentration (MBC), and antibiofilm assays. Strikingly, co-precipitated samples (BA1–BA3) exhibited negligible to zero antibacterial activity. In contrast, solvothermally synthesized nanoparticles (BA4–BA6) demonstrated potent, dose-dependent antibacterial activity, with inhibition zones reaching up to 28 mm against *E. coli* and 33 mm against *B. cereus* at 10 μg/mL. The most active sample, BA6 (solvothermal, 300 °C calcination), exhibited MIC values of 200 μg/mL against *E. coli* and 100 μg/mL against *B. cereus*, with corresponding MBC values of 400 μg/mL and 200 μg/mL, respectively. The close correlation between MIC and MBC values indicates a rapid transition from growth inhibition to bactericidal action. In antibiofilm assays, BA5 and BA6 demonstrated the highest efficacy in suppressing mature biofilm formation of both bacterial strains at concentrations of 200–800 μg/mL, as quantified by crystal violet staining. The superior performance of solvothermal nanoparticles was attributed to their larger crystallite size, higher crystallinity, and favorable surface morphology, which enhance interaction with bacterial membranes and promote ROS generation, leading to membrane disruption, oxidative stress, and cell death.

Jarhad et al. [82] successfully developed a green, cost-effective, and eco-friendly method for synthesizing multifunctional CdS as nanoparticles using an aqueous methanolic extract of cinnamon (*Cinnamomum verum*) sticks, which served as both a reducing and stabilizing agent. The phytochemicals present in the extract, particularly polyphenols and flavonoids, facilitated the reduction of Cd^2+^ ions and capped the nanoparticles, preventing agglomeration and enhancing biocompatibility. Comprehensive characterization confirmed the formation of crystalline CdS with a hexagonal wurtzite structure, an average crystallite size of approximately 5 nm (calculated via the Debye–Scherrer equation), and a spherical to irregular morphology, as revealed by XRD, SEM, and HR-TEM analyses. FTIR spectroscopy confirmed the presence of biomolecules on the nanoparticle surface, while UV-Vis spectroscopy showed a blue-shifted absorption edge with a calculated direct bandgap of 2.91 eV, indicating quantum confinement effects. The pH at the point of zero charge (pHZPC) was determined to be approximately 6.5, indicating surface charge neutrality at this pH. The photocatalytic activity of the CdS was evaluated for the degradation of methylene blue (MB) dye under both UV light and natural sunlight. The nanoparticles exhibited excellent degradation efficiencies, achieving 91% degradation under UV light within 90 min and 94% under sunlight within 70 min, following pseudo-first-order kinetics. The enhanced performance under sunlight was attributed to increased temperature and broader spectral absorption. Photoelectrochemical (PEC) studies, including linear sweep voltammetry (LSV) and chopped light voltammetry (CLV), confirmed the n-type semiconductor behavior of the CdS, with efficient charge separation and a photocurrent density of 0.42 mA cm^−2^, demonstrating their potential for solar energy conversion applications. In biomedical assays, the CdS exhibited moderate antibacterial activity against both Gram-positive (*Staphylococcus aureus*, *Bacillus subtilis*) and Gram-negative (*Escherichia coli*, *Pseudomonas aeruginosa*) bacteria using the disk diffusion method, with inhibition zones ranging from approximately 8 to 18 mm, showing slightly higher efficacy against Gram-positive strains due to their simpler cell wall structure. The nanoparticles also demonstrated significant, dose-dependent cytotoxicity against MCF-7 breast cancer cells in an MTT assay, with an IC_50_ value of 110 μg/mL, compared to 31.63 μg/mL for the standard drug cisplatin. This anticancer activity is likely enhanced by the synergistic effect of surface-bound cinnamon phytochemicals, such as cinnamaldehyde, which are known to induce apoptosis. Biocompatibility was assessed via a hemolysis assay using human red blood cells, revealing that the CdS caused less than 5% hemolysis even at the highest tested concentration (120 μg/mL), classifying them as slightly hemolytic and biocompatible at lower concentrations. This study highlights the potential of cinnamon extract-mediated CdS as a versatile nanomaterial for environmental remediation (dye degradation, solar energy conversion) and biomedical applications (antibacterial agents, cancer therapy), offering a sustainable and multifunctional platform for future technologies.

Borah et al. [83] successfully developed a low-cost, eco-friendly green strategy for synthesizing fluorescent CdS as QD using an aqueous extract of the cyanobacterium *Nostoc carneum*. The phytochemicals present in the extract, particularly carbohydrates and proteins, acted as effective stabilizing and capping agents, preventing agglomeration and ensuring the formation of stable, biocompatible QDs. Comprehensive characterization confirmed the successful synthesis of highly crystalline, spherical CdS with a cubic zinc blende structure, an average particle size of 2–6 nm (as determined by TEM), and a hydrodynamic diameter of 17.3 nm (from DLS). UV-Vis spectroscopy revealed an absorption edge at 423 nm with a calculated bandgap of 2.68 eV, indicating a blue shift due to quantum confinement compared to bulk CdS (2.42 eV). The QDs exhibited strong photoluminescence, with a broad emission band centered at 515 nm upon excitation at 425 nm, attributed to excitonic recombination and surface trap states. FTIR spectroscopy confirmed the presence of biomolecules on the QD surface, while EDX analysis verified the elemental composition of Cd and S with high purity. The photocatalytic activity of the CdS was evaluated for the degradation of two toxic organic dyes, methyl orange (MO) and rhodamine B (RhB), under laboratory scattered sunlight. The QDs demonstrated exceptional degradation efficiencies, achieving 91.4% degradation of MO and 90.0% degradation of RhB within 80 min, following pseudo-first-order kinetics with rate constants of 0.02896 min^−1^ and 0.02846 min^−1^, respectively. The catalyst exhibited excellent reusability, maintaining high efficiency over four consecutive cycles. The degradation mechanism was attributed to the generation of ROS, such as O_2_^−^ and •OH, upon photoexcitation of the CdS, which oxidize the dye molecules into non-toxic byproducts (CO_2_, H_2_O). The antibacterial activity of the CdS was assessed against four pathogenic bacterial strains: Gram-negative *Klebsiella pneumoniae*, *Pseudomonas aeruginosa*, *Escherichia coli*, and Gram-positive *Staphylococcus aureus*, using the disk diffusion method. The QDs exhibited significant, dose-dependent antibacterial activity, with inhibition zones ranging from approximately 6 to 12 mm at concentrations of 10–40 μg/mL. The highest activity was observed against *K. pneumoniae* (11 mm at 40 μg/mL). The proposed antibacterial mechanism involves electrostatic attraction between positively charged CdS and negatively charged bacterial cell membranes, leading to membrane disruption, generation of ROS, oxidative stress, and inhibition of DNA and RNA synthesis, ultimately causing cell death. The smaller size of the QDs (2–6 nm) provides a larger surface area for interaction, enhancing their bactericidal efficacy. This study highlights the potential of cyanobacterium-mediated CdS as multifunctional nanomaterials for environmental remediation (photocatalytic dye degradation) and biomedical applications (antibacterial agents), offering a sustainable and biocompatible alternative to chemically synthesized nanoparticles.

Jarhad et al. [84] successfully developed a novel, eco-friendly, one-pot method for synthesizing fluorescent CdS as QD at room temperature using aqueous methanolic extracts of two common Indian spices: green cardamom (*Elettaria cardamomum*) pods (CdS-Cm QDs) and ginger (*Zingiber officinale*) rhizomes (CdS-Gi QDs). The phytochemicals present in the extracts, such as limonene, eucalyptol, and gingerol, acted as effective reducing, stabilizing, and capping agents, facilitating the formation of quantum-confined nanoparticles and imparting enhanced biological properties. Comprehensive characterization confirmed the successful synthesis of amorphous, spherical QDs with particle sizes in the range of 2–5 nm, as determined by HR-TEM and XRD (using the Debye–Scherrer equation, which gave crystallite sizes of 2.03 nm for CdS-Cm and 1.93 nm for CdS-Gi). UV-Vis spectroscopy revealed absorption peaks at 449 nm and 447 nm for CdS-Cm and CdS-Gi QDs, respectively, with corresponding bandgaps of 3.00 eV and 3.06 eV, indicating a significant blue shift due to quantum confinement. FTIR spectroscopy confirmed the presence of various functional groups (O-H, C=O, C-O) from the spice phytochemicals on the QD surface, along with the characteristic Cd-S bond at 432 cm^−1^. Zeta potential measurements showed strong negative surface charges (−37 mV for CdS-Cm, −33 mV for CdS-Gi), indicating excellent colloidal stability. The anticancer activity of the synthesized QDs was rigorously evaluated against human breast cancer cells (MCF-7) using multiple assays. The MTT assay revealed that CdS-Cm QDs exhibited potent, dose-dependent cytotoxicity with an IC_50_ value of 39.03 μM, which was remarkably close to that of the standard chemotherapeutic drug cisplatin (30.76 μM), while CdS-Gi QDs showed a higher IC_50_ of 63.03 μM. The enhanced activity of CdS-Cm QDs was attributed to the synergistic effect of cardamom’s monoterpenes (α-pinene, limonene), which can induce apoptosis by disrupting mitochondrial function and causing endoplasmic reticulum stress. Lactate dehydrogenase (LDH) leakage assays confirmed concentration-dependent loss of cell membrane integrity, with IC_50_ values of 27.3 μg/mL for CdS-Cm and 63 μg/mL for CdS-Gi. Apoptosis was further confirmed through AO/EB and PI staining, which showed characteristic morphological changes (chromatin condensation, nuclear fragmentation) and a significant increase in apoptotic/necrotic cells upon treatment, with CdS-Cm QDs exhibiting a more profound effect. Biocompatibility was assessed via an erythrocyte lysis assay using human AB+ blood, which showed that both QDs caused less than 2.5% hemolysis even at high concentrations (up to 60 μg/mL), well within the acceptable limit of 5%, confirming their hemocompatibility. The QDs also demonstrated significant antioxidant activity in a DPPH radical scavenging assay, with CdS-Cm QDs showing a maximum inhibition of 67.58% at 500 μg/mL, attributed to the antioxidative phytochemical corona on the QD surface. Furthermore, the antibacterial activity was evaluated against Gram-positive (*Bacillus subtilis*, *Staphylococcus aureus*) and Gram-negative (*Escherichia coli*, *Klebsiella pneumoniae*) bacteria using the disk diffusion method. Both QDs exhibited dose-dependent antibacterial activity, with inhibition zones ranging from approximately 8 to 35 mm at concentrations of 60–100 μg/mL. The enhanced antibacterial effect was attributed to the synergistic action of the CdS and the bioactive spice compounds (e.g., gingerols, terpenes), which can disrupt bacterial cell membranes and facilitate QD penetration. This comprehensive study highlights the potential of spice-extract-mediated CdS, particularly those capped with green cardamom, as promising multifunctional nanobiomaterials for anticancer therapy, antioxidant applications, and antibacterial treatments, offering a sustainable and biocompatible alternative to chemically synthesized nanoparticles.

Rajbongshi and Kalita [85] successfully synthesized CdS with three distinct morphologies, spherical nanoparticles, nanopetals, and rose-like hierarchical structures, using a simple wet chemical method by systematically varying the concentration of triethanolamine (TEA) as a capping and structure-directing agent. Comprehensive characterization confirmed the successful formation of phase-pure, well-crystalline CdS with a hexagonal wurtzite structure across all morphologies. SEM revealed a clear morphological transformation from uniformly distributed spherical nanoparticles (at 0.01 M TEA) to nanopetal assemblies (at 0.05 M TEA) and finally to complex, three-dimensional rose-like hierarchical structures (at 0.1 M TEA), demonstrating the critical role of TEA concentration in morphology control. UV-Vis diffuse reflectance spectroscopy showed a morphology-dependent red shift in the absorption edge, with calculated bandgaps of 2.42 eV, 2.38 eV, and 2.3 eV for spherical, nanopetal, and rose-like CdS, respectively, indicating enhanced visible-light absorption for the rose-like structure. PL spectroscopy revealed a strong emission peak at ~576.6 nm for all samples, attributed to sulfur vacancy-related defect states, with the rose-like CdS exhibiting the highest PL intensity, indicating the presence of more surface defects that can act as electron–hole separation centers. Nitrogen adsorption–desorption measurements confirmed that the rose-like CdS possessed the highest specific surface area (8.93 m^2^/g) and largest pore diameter (5.25 nm) compared to spherical (4.18 m^2^/g) and nanopetal (6.64 m^2^/g) structures, classifying them as mesoporous materials (type IV isotherm, H3 hysteresis). EIS Nyquist plots demonstrated that the rose-like CdS electrode exhibited the smallest arc radius, indicating the lowest charge transfer resistance and highest electron transfer efficiency, which is crucial for photocatalytic applications. The photocatalytic activity was evaluated through the degradation of MB dye under direct sunlight irradiation. The rose-like CdS nanostructure exhibited superior photocatalytic performance, achieving 96.5% degradation within 120 min, significantly higher than nanopetal (71.9%) and spherical (48.6%) CdS, following pseudo-first-order kinetics with rate constants of 0.0301 min^−1^, 0.0114 min^−1^, and 0.0055 min^−1^, respectively. The enhanced activity was attributed to the synergistic effects of the rose-like morphology: a higher surface area providing more active sites, enhanced light absorption due to a narrower bandgap and light scattering within the hierarchical structure, efficient charge separation and transfer due to surface defects and reduced recombination, and the mesoporous nature facilitating dye adsorption. Total organic carbon (TOC) analysis confirmed 92% mineralization of MB, and atomic absorption spectroscopy (AAS) revealed minimal Cd^2+^ leaching (0.0031 ppm) after photocatalysis, indicating good photostability due to TEA encapsulation. The antibacterial activity of the CdS nanostructures was assessed against Gram-positive (*Staphylococcus aureus*, *Bacillus subtilis*) and Gram-negative (*Enterobacter aerogenes*, *Escherichia coli*) bacteria using the disk diffusion method. All morphologies exhibited dose-dependent antibacterial activity, with inhibition zones ranging from approximately 9 to 35 mm. Notably, the rose-like CdS nanostructure demonstrated the highest antibacterial efficacy against all tested strains, producing inhibition zones of 34.7 mm against *S. aureus* and 22.6 mm against *E. aerogenes*, outperforming both the spherical and nanopetal structures as well as the antibiotic control (Streptomycin) in some cases. The enhanced antibacterial activity was attributed to the smaller size, higher surface area, and greater defect density of the rose-like nanostructure, which facilitate stronger electrostatic interactions with bacterial cell membranes, leading to membrane disruption, generation of ROS, and ultimately cell death. This study conclusively demonstrates that morphology engineering, particularly the creation of hierarchical rose-like structures, is a powerful strategy for simultaneously enhancing the photocatalytic and antibacterial performance of CdS, positioning them as promising multifunctional agents for environmental remediation and biomedical applications.

Abd-Elkader and Shaltout [86] successfully deposited nanocrystalline CdS as thin films onto glass and silicon substrates using a low-cost, facile CBD method, with polyvinylpyrrolidone (PVP) employed as a capping agent to prevent agglomeration. By optimizing deposition parameters (stirring speed, temperature, pH, and time), they obtained uniform, adherent films of varying thicknesses (50, 80, and 100 nm). Comprehensive characterization confirmed the formation of high-quality films: XRD revealed that films deposited on glass were polycrystalline with a hexagonal (wurtzite) structure, while those on silicon were predominantly amorphous. The average grain size for the crystalline films was calculated to be approximately 16.29 nm using the Debye–Scherrer equation. TEM and selected area electron diffraction (SAED) further confirmed the nanocrystalline nature, with grain sizes around 30 nm. SEM showed uniform, crack-free surfaces, and EDX verified the elemental composition of Cd and S. UV-Vis-NIR spectroscopy yielded an optical bandgap of 2.4 eV, consistent with bulk CdS. The antibacterial activity of the 100 nm thick CdS films was evaluated against Gram-positive *Staphylococcus aureus* and Gram-negative *Pseudomonas aeruginosa* and *Escherichia coli* using the agar disk diffusion method, with experiments conducted both in the dark and under sunlight at 60 °C. The films exhibited significant antibacterial activity under both conditions, with markedly enhanced efficacy under sunlight irradiation. Epifluorescence microscopy of acridine orange-stained bacterial cells revealed that active, live bacteria emitted orange-red fluorescence, while areas treated with CdS films showed reduced fluorescence and fewer attached cells, indicating effective bactericidal action. The enhanced activity under sunlight was attributed to the photocatalytic generation of ROS by the CdS semiconductor upon light absorption, leading to oxidative stress, membrane damage, and cell death. This study demonstrates that nanocrystalline CdS thin films, particularly when photoactivated, possess strong antibacterial potential and could serve as effective antimicrobial coatings for medical and environmental applications.

Ayodhya and Veerabhadram [87] successfully developed a simple, eco-friendly, one-pot sonochemical method for synthesizing CdS as nanoparticles capped with an aqueous extract of *Calotropis gigantea* leaves, which served as both a reducing and stabilizing agent due to its rich phytochemical content (flavonoids, terpenoids, phenols, and proteins). Comprehensive characterization confirmed the formation of well-dispersed, spherical CdS with a cubic zinc blende structure, an average particle size of approximately 20 nm (from TEM, consistent with XRD analysis using the Debye–Scherrer equation), and a negative zeta potential of −31.6 mV, indicating excellent colloidal stability. UV-Vis diffuse reflectance spectroscopy (DRS) revealed a blue-shifted bandgap of 2.54–2.61 eV compared to bulk CdS (2.42 eV), confirming quantum confinement effects, while photoluminescence (PL) spectroscopy showed emission peaks at 409, 432, and 507 nm, attributed to band edge recombination and surface state passivation by the capping phytochemicals. FTIR and Raman spectroscopy confirmed the presence of biomolecules on the nanoparticle surface and the characteristic longitudinal optical (LO) phonon modes of cubic CdS, respectively. The photocatalytic activity of the bio-capped CdS was evaluated for the degradation of MB and eosin Y (EY) dyes under sunlight irradiation. The nanoparticles exhibited efficient, time-dependent degradation, achieving 85.53% degradation of MB and 91.12% degradation of EY within 60 min, following pseudo-first-order kinetics with rate constants of 0.0241 min^−1^ and 0.0332 min^−1^, respectively. The enhanced photocatalytic performance was attributed to the synergistic effects of the narrow bandgap facilitating visible-light absorption, the high surface area of the nanoparticles, and the effective separation of photogenerated electron–hole pairs. The catalyst demonstrated good stability and reusability, retaining >81% degradation efficiency after five cycles. The CdS nanoparticles were also explored as a fluorescent probe for the selective detection of metal ions via spectrofluorometry. Upon the addition of various metal ions (Ba^2+^, Ca^2+^, Sn^2+^, V^2+^, Mg^2+^, Sr^2+^, Pb^2+^, Co^2+^, Zn^2+^, Ni^2+^, Cu^2+^, Hg^2+^, Mn^2+^) at nanomolar concentrations, a significant and selective fluorescence quenching was observed specifically for Pb^2+^, Sn^2+^, and Hg^2+^, with the most pronounced effect for Pb^2+^, indicating strong affinity between these ions and the surface functional groups (hydroxyl, amine) from the capping agent, leading to efficient electron transfer and non-radiative recombination. The antimicrobial activity of the *C. gigantea*-capped CdS was evaluated against a panel of pathogenic bacteria (*Escherichia coli*, *Pseudomonas aeruginosa*, *Staphylococcus aureus*, *Bacillus thuringiensis*) and fungi (*Aspergillus niger*, *Candida albicans*) using the disk diffusion method. The bio-capped CdS exhibited significantly enhanced antimicrobial activity compared to the uncapped nanoparticles or the leaf extract alone, with inhibition zones ranging from approximately 21 to 31 mm for bacteria and 24 to 28 mm for fungi at a concentration of 0.15 μg/mL. The highest antibacterial activity was observed against *S. aureus* (31 mm), and the highest antifungal activity against *A. niger* (28 mm). Time-dependent cell viability assays confirmed a progressive loss of microbial cell viability over 6 h, with the highest death rates observed in the final hours. The enhanced antimicrobial mechanism was attributed to the synergistic action of the CdS and the surface-bound bioactive phytochemicals, which can generate ROS, disrupt cell membranes, interact with thiol groups of essential enzymes, and interfere with DNA replication, ultimately leading to cell death. This comprehensive study highlights the potential of *C. gigantea* leaf extract as an effective, renewable capping agent for the green synthesis of multifunctional CdS with promising applications in environmental remediation (photocatalytic dye degradation), analytical sensing (selective heavy metal ion detection), and biomedical fields (antimicrobial agents).

Shivaji et al. [88] successfully developed a novel, eco-friendly, biogenic synthesis route for producing fluorescent CdS as QD with an ultra-small particle size of 2–5 nm using an extract of tea leaves (*Camellia sinensis*). The phytochemicals present in the tea extract, particularly polyphenols, proteins, and amino acids, acted as effective, non-toxic stabilizing and capping agents, enabling the formation of highly homogeneous, spherical QDs with a quantum-confined bandgap of 3.03 eV, as confirmed by HRTEM, EDS, FTIR, and UV-Vis spectroscopy. The study comprehensively explored the biological activity of these green CdS across multiple applications. Antibacterial testing via the well diffusion method against a panel of pathogenic bacteria, including Gram-negative *Escherichia coli*, *Serratia marcescens*, *Klebsiella pneumoniae* and Gram-positive *Streptococcus pyogenes*, *Staphylococcus aureus*, demonstrated clear, dose-dependent bactericidal activity. The CdS produced inhibition zones ranging from approximately 9 to 21 mm at concentrations of 40–200 μg/mL, with the highest activity observed against *S. marcescens* (21 mm at 200 μg/mL), attributed to their small size enabling effective penetration of bacterial cell walls. Biocompatibility was confirmed through a hemolysis assay using human red blood cells (RBCs), which showed that the CdS caused minimal hemolysis (only 1.83% at 60 μg/mL), well within the permissible 5% limit for biomedical applications, indicating excellent hemocompatibility. Critically, the CdS exhibited potent, dose-dependent cytotoxicity against A549 lung cancer cells in an MTT assay, with efficacy comparable to the standard drug cisplatin. Mechanistic studies using fluorescence microscopy (AO/EtBr and DAPI staining) revealed that the QDs induce apoptosis, evidenced by chromatin condensation, nuclear fragmentation, and membrane blebbing. Flow cytometry analysis confirmed that the CdS arrest the cell cycle specifically at the S phase, inhibiting DNA replication and leading to programmed cell death. Furthermore, the intrinsic fluorescence of the QDs enabled high-contrast intracellular bioimaging of A549 cells without the need for additional fluorescent tags. This study highlights the remarkable multifunctional potential of tea leaf extract-mediated CdS as a promising, cost-effective nanomaterial platform for integrated antibacterial therapy, biocompatible imaging, and targeted cancer treatment, paving the way for future in vivo biomedical applications.

## 7. Antibacterial Performance of CdS/X Binary Composites Materials

The construction of binary heterojunctions, coupling CdS with a second semiconductor (e.g., TiO_2_, ZnO, Ag_2_S), a conductive carbonaceous support (graphene oxide, reduced graphene oxide, carbon nanotubes), or a noble metal, offers a well-established strategy to overcome these limitations. In such binary systems, the second component serves multiple synergistic functions: it acts as an electron sink to prolong charge carrier lifetimes, generates additional ROS through complementary redox pathways, releases secondary antimicrobial ions (e.g., Ag^+^, Zn^2+^), or physically disrupts bacterial membranes via sharp edges or high surface area. This section critically reviews the antibacterial performance of CdS-based binary composites, emphasizing the structure–activity relationships that emerge from different heterojunction architectures. Key parameters such as optimal loading ratios, charge transfer mechanisms (Type II vs. Schottky), and illumination conditions are discussed. A detailed tabulated summary is presented in Table 3, enabling direct comparison of inhibition zones, MIC values, and unique mechanistic features across diverse binary systems.

Iqbal et al. [89] successfully synthesized cadmium sulfide–silver sulfide (CdS-Ag_2_S) nanocomposites with varying CdS ratios (5%, 10%, and 15%) using a facile and economical co-precipitation method, with polyvinylpyrrolidone (PVP) employed as an effective capping agent to control particle morphology and prevent agglomeration (Table 3). Comprehensive characterization confirmed the successful formation of the nanocomposites: XRD analysis revealed the monoclinic β-Ag_2_S (acanthite) phase alongside hexagonal CdS peaks at higher concentrations, with crystallite sizes decreasing from 12.2 nm for pure Ag_2_S to 9.6 nm for the 15% CdS composite, attributed to the substitution of larger Ag^+^ ions (1.26 Å) with smaller Cd^2+^ ions (0.97 Å). SEM images showed spherical nanoparticles with a size distribution of 50–100 nm, while EDX confirmed the presence of Ag, Cd, and S elements. FTIR spectroscopy verified metal–sulfur (Ag-S, Cd-S) bond formation and the presence of surface-adsorbed species. The antibacterial activity of the pure and composite materials was rigorously evaluated against three pathogenic bacteria using the well diffusion method: Gram-negative *Pseudomonas aeruginosa*, *Escherichia coli*, and Gram-positive *Staphylococcus aureus*. The results demonstrated a clear synergistic enhancement in antibacterial efficacy for the CdS-Ag_2_S nanocomposites compared to the individual components. While pure Ag_2_S and CdS exhibited moderate activity, the 10% CdS-Ag_2_S nanocomposite showed the highest bactericidal performance, producing substantial inhibition zones of 35.88 mm against *P. aeruginosa*, 39.24 mm against *E. coli*, and 30.23 mm against *S. aureus*, corresponding to bacterial reductions of 80.3%, 90.7%, and 53.8%, respectively. This optimal composite also exhibited the lowest minimum inhibitory concentration (MIC) values. The enhanced activity is attributed to the synergistic interplay between the two semiconductors: the reduced crystallite size at optimal CdS loading increases the surface area for bacterial interaction, while the formation of a heterojunction between CdS and Ag_2_S facilitates efficient charge separation upon light exposure, leading to enhanced generation of ROS such as superoxide and hydroxyl radicals. These ROS, combined with the release of antimicrobial Ag^+^ ions, cause oxidative stress, membrane damage, and ultimately bacterial cell death. This study highlights the potential of PVP-capped CdS-Ag_2_S nanocomposites, particularly at an optimal 10% CdS ratio, as highly effective, broad-spectrum antibacterial agents for potential applications in medical devices, home appliances, and environmental disinfection.

Rafique et al. [90] successfully synthesized pristine CdS as QDs and cellulose nanocrystal-grafted polyvinylpyrrolidone (CNC-g-PVP)-doped CdS (at 2, 4, and 6% doping concentrations) using a facile and economical co-precipitation method, aiming to create multifunctional nanomaterials for environmental and biomedical applications. Comprehensive characterization confirmed the successful incorporation of the dopant and its influence on material properties: XRD verified the hexagonal wurtzite structure of CdS, with crystallite size decreasing from 9.05 nm (pristine) to 7.05 nm (6% doped) upon doping, while no significant peak shifts were observed. UV-Vis spectroscopy revealed a blue shift in the absorption edge with increasing dopant concentration, corresponding to a bandgap increase from 2.45 eV (pristine CdS) to 2.51 eV (6% doped CdS), attributed to the quantum confinement effect from reduced particle size (confirmed by TEM, which showed a decrease from 9.28 nm to 7.15 nm). FTIR spectroscopy confirmed the presence of functional groups from both CdS and the CNC-g-PVP dopant, including O-H, C-N, C-O, and Cd-S bonds. Photoluminescence (PL) spectra showed a single green emission band around 550 nm, with intensity increasing upon doping, indicating a higher recombination rate of electron–hole pairs. The catalytic activity of the QDs was evaluated for the degradation of MB dye in neutral, acidic, and basic media using NaBH_4_ as a reducing agent. The doped QDs exhibited significantly enhanced, pH-dependent catalytic performance, with the 6% doped CdS achieving 99.09% degradation in neutral medium (11 min), 95.20% in acidic medium (5 min), and 99.79% in basic medium (8 min), outperforming pristine CdS and the dopant alone. This enhanced activity was attributed to the increased surface area, reduced particle size, and improved electron transfer mediated by the CNC-g-PVP matrix. The antibacterial activity was assessed against mastitis-causing pathogens, Gram-positive *Staphylococcus aureus* and Gram-negative *Escherichia coli*, using the agar well diffusion method at concentrations of 500 and 1000 μg/50 μL. Pristine CdS showed minimal activity, while CNC-g-PVP alone showed none. However, the doped QDs exhibited significant, dose-dependent antibacterial effects, with the 6% doped sample producing the largest inhibition zones: 5.25 mm against *S. aureus* and 4.05 mm against *E. coli* at the highest concentration, corresponding to 84% and 95.5% efficiency relative to the ciprofloxacin control, respectively. The enhanced activity against Gram-negative *E. coli* was attributed to its thinner cell wall, facilitating QD penetration. The antibacterial mechanism was proposed to involve electrostatic interaction between cationic Cd^2+^ ions and anionic bacterial membranes, generation of ROS, and disruption of membrane integrity, leading to cell death. To rationalize these findings, molecular docking studies were performed against two key bacterial enzyme targets, DNA gyrase (from *E. coli*) and FabI (enoyl-ACP reductase, from *S. aureus*), essential for nucleic acid and fatty acid synthesis, respectively. In silico predictions showed that CNC-g-PVP doped CdS had significantly higher binding affinities (−8.773 kcal/mol for DNA gyrase; −9.756 kcal/mol for FabI) compared to the dopant alone, forming multiple hydrogen bonds and hydrophobic interactions with active site residues, suggesting that the nanocomposites act as effective enzyme inhibitors, providing a mechanistic basis for their bactericidal action. This study demonstrates that CNC-g-PVP-doped CdS are promising, eco-friendly nanomaterials for rapid catalytic dye degradation and potent antibacterial applications, with molecular docking providing valuable insights into their mode of action.

Lim et al. [91] successfully synthesized, for the first time, chitin-encapsulated cadmium sulfide quantum dots (CdS@CTN QDs) using a colloidal chemistry method, aiming to develop biocompatible and biodegradable QDs for potential biomedical applications. Chitin, isolated from mud crab (*Scylla serrata*) shells through demineralization and deproteinization, served as a natural biopolymer matrix for encapsulation. Comprehensive characterization confirmed the successful formation of core–shell QDs: UV-Vis spectroscopy showed an absorption peak at 460 nm, corresponding to an average particle size of 3.9 nm estimated using the Henglein model. X-ray diffraction (XRD) analysis revealed the coexistence of two crystalline phases—cubic sphalerite (zinc blende) CdS, with diffraction peaks at 26.7°, 43.7°, and 52.1° indexed to the (111), (220), and (311) planes, and α-chitin, with a characteristic peak at 26.5°. The crystallite size of the CdS core was calculated to be 4.3 nm using the Scherrer equation, slightly larger than that of conventional 3-mercaptopropionic acid (MPA)-capped CdS (3.6 nm) synthesized for comparison. FTIR spectroscopy confirmed the presence of chitin on the QD surface, with characteristic bands for O-H, N-H, amide C=O, and Cd-S vibrations, and indicated chelation between Cd^2+^ ions and the amide/hydroxyl groups of chitin. The effect of synthesis parameters (pH and chitin-to-cadmium precursor mass ratio) was investigated, revealing that higher pH (up to 12) and an optimal chitin mass of 0.2 g for 22 mg of Cd(NO_3_)_2_ precursor promoted greater chitin coordination and crystallinity on the QD surface. The antibacterial activity of the CdS@CTN QDs was screened against four bacterial strains: Gram-positive *Bacillus subtilis* and *Staphylococcus aureus*, and Gram-negative *Escherichia coli* and *Pseudomonas aeruginosa*, using the agar well diffusion method at a concentration of 2.0 mg/mL. In this preliminary screening, neither the CdS@CTN QDs nor the MPA-capped CdS exhibited any observable zone of inhibition against any of the tested bacteria, while the positive control (streptomycin sulfate) produced clear inhibition zones of 20–25 mm. This lack of activity was not attributed to inherent non-toxicity of the QDs but rather to two main factors: the high biocompatibility of the chitin encapsulation layer effectively masking the CdS core and potentially rendering the QDs inert, and the possibility that the QDs did not diffuse effectively through the agar medium under the test conditions. The study concludes that while the CdS@CTN QDs did not show antibacterial activity in this assay, the successful encapsulation with biocompatible chitin makes them promising candidates for other biomedical applications, such as fluorescent labels, bioimaging probes, and drug carriers, where biocompatibility and controlled interaction are paramount. The authors suggest that further studies, including MIC and MBC determinations in liquid media, are necessary to fully elucidate the antibacterial potential and biocompatibility of these novel chitin-encapsulated QDs.

Byeon et al. [92] successfully synthesized undoped CdS and zirconia-doped CdS (designated CZ1, CZ2, and CZ3 with 1, 2, and 3 wt% ZrO_2_, respectively) using an eco-friendly green approach with Moringa oleifera seed extract as a reducing and stabilizing agent. Comprehensive characterization confirmed the successful incorporation of zirconia and its profound influence on material properties: XRD revealed a phase transition from cubic to hexagonal CdS with increasing zirconia content, accompanied by a significant reduction in crystallite size from 31.18 nm (pure CdS) to 18.4 nm (CZ3). UV-Vis spectroscopy showed a blue shift in the absorption edge upon doping, with calculated bandgaps increasing from 2.1 eV (CdS) to 3.4 eV (CZ3), attributed to quantum confinement effects and structural modifications. FTIR spectroscopy confirmed the presence of bio-organic residues from the plant extract and metal–sulfur (Cd-S) and zirconium–oxygen (Zr-O) bonds. FESEM imaging revealed a progressive morphological transformation from aggregated nanospheres in pure CdS to more defined, fragmented, and well-dispersed nanoparticles with increased surface roughness in CZ3, while EDX and elemental mapping confirmed the homogeneous distribution of Cd, S, Zr, and residual carbon. The photocatalytic activity was evaluated against Rhodamine B (RhB, cationic) and Eosin Yellow (EY, anionic) dyes under sunlight irradiation. The CZ3 nanocomposite exhibited the highest degradation efficiency, achieving 92.3% for RhB and 90.6% for EY within 120 min, significantly outperforming pure CdS (75.7% and 68.2%, respectively). The enhanced performance was attributed to the synergistic effects of zirconia doping: reduced particle size (27.8 nm for CZ3) providing higher surface area, an optimized bandgap reducing electron–hole recombination, and improved charge carrier dynamics. Kinetic analysis confirmed that the degradation followed a pseudo-first-order model, with CZ3 showing rate constants of 1.8 × 10^−2^ min^−1^ (RhB) and 1.7 × 10^−2^ min^−1^ (EY), 1.6 and 2.1 times higher than pure CdS, respectively. The nanocomposites also exhibited excellent cyclic stability, with CZ3 retaining 97.95% (RhB) and 96.6% (EY) of its initial activity after five cycles, attributed to zirconia’s protective effect against photocorrosion. The antibacterial activity was rigorously evaluated against Gram-positive *Staphylococcus aureus* and Gram-negative *Escherichia coli* using both ZOI and turbidimetric growth inhibition assays at concentrations of 25, 50, and 100 μg/mL. The results demonstrated clear concentration-dependent and doping-dependent antibacterial efficacy. At 100 μg/mL, CZ3 produced the largest inhibition zones: 19 mm against *S. aureus* and 17 mm against *E. coli*, compared to 12 mm and 10 mm for pure CdS, respectively. Growth inhibition efficiency reached 92.1% against *S. aureus* and 87.33% against *E. coli* for CZ3 at the highest concentration. The higher susceptibility of *S. aureus* was attributed to its thicker peptidoglycan layer being more vulnerable to ROS-induced oxidative stress, while the outer membrane of *E. coli* provides an additional barrier. The antibacterial mechanism was proposed to involve enhanced ROS generation (superoxide and hydroxyl radicals) facilitated by zirconia-induced charge separation, leading to oxidative damage to cell membranes and cellular components. Additionally, the nanocomposites exhibited significant, concentration-dependent antioxidant activity in DPPH radical scavenging assays, with CZ3 achieving 89.6% scavenging at 100 μg/mL, attributed to the enhanced electron transfer capacity of the doped material. This comprehensive study demonstrates that green-synthesized zirconia-doped CdS, particularly the CZ3 formulation, are promising multifunctional materials with enhanced photocatalytic, antibacterial, and antioxidant properties, suitable for environmental remediation and biomedical applications.

Okla et al. [93] successfully synthesized cadmium sulfide–silver sulfide nanocomposites (CdS/Ag_2_S NCs) with varying Ag_2_S content (10%, 30%, 50%, and 80%) using a simple, one-step chemical co-precipitation method, aiming to create a multifunctional material with enhanced visible-light-driven photocatalytic and antimicrobial properties. Comprehensive characterization confirmed the successful formation of spherical, well-dispersed NCs with a narrow size range of 10–15 nm, composed of cubic CdS and monoclinic Ag_2_S phases, as evidenced by XRD, TEM, HRTEM, and SAED analysis. The CdS/Ag_2_S-50% NCs exhibited an optimal bandgap of 2.12 eV (compared to 2.43 eV for pure CdS and 1.62 eV for pure Ag_2_S), facilitating efficient visible-light absorption. BET analysis revealed a high surface area of 57.23 m^2^/g for the NCs, significantly higher than pure CdS (31.18 m^2^/g) and Ag_2_S (26.52 m^2^/g), providing abundant active sites for catalytic reactions. XPS analysis confirmed the presence of Cd, Ag, and S with expected oxidation states, and a slight shift in binding energies indicated strong electronic interaction and heterojunction formation between CdS and Ag_2_S. PL spectroscopy and EIS analysis demonstrated that the NCs effectively suppressed electron–hole recombination and facilitated charge transfer, crucial for photocatalytic activity. The photocatalytic performance was evaluated by degrading methylene blue (MB) dye under visible-light irradiation. The CdS/Ag_2_S-50% NCs exhibited excellent activity, achieving 95 ± 2.4% degradation of 25 mg/L MB within 60 min at optimal pH 8, significantly outperforming pure CdS (62%) and Ag_2_S (23%). This enhanced activity was attributed to the Type I heterojunction formed between CdS and Ag_2_S, which promotes efficient charge separation and migration of photogenerated electrons and holes, leading to enhanced generation of ROS. Scavenger studies identified •O_2_^−^ as the primary reactive species responsible for degradation. The NCs demonstrated excellent stability and reusability, retaining 94.3% degradation efficiency after six cycles, with XRD and XPS confirming structural integrity. TOC analysis showed 87% mineralization, indicating effective breakdown of dye into CO_2_ and H_2_O. The antibacterial activity was rigorously evaluated against Gram-positive *Staphylococcus aureus* (ATCC 25923) and two strains of Gram-negative *Escherichia coli* (ATCC 25922 and ATCC 13534) using a colony counting method at very low concentrations (0.1–10 mg/L) under both dark and visible-light conditions. In the dark, the NCs (10 mg/L) inhibited bacterial growth by 94% (*E. coli* 25922), 91% (*E. coli* 13534), and 79% (*S. aureus*). Under visible-light irradiation, the bactericidal efficacy was dramatically enhanced, reaching >99.9% inhibition for all strains at 10 mg/L, demonstrating the powerful synergistic effect of the photocatalytic generation of ROS. The NCs were more effective against Gram-negative *E. coli* due to its thinner peptidoglycan layer facilitating particle penetration. The antibiofilm activity was assessed against pre-formed biofilms and biofilm formation on NC-coated surfaces. Against pre-formed biofilms under light, 10 mg/L NCs achieved 48% (*E. coli* 25922), 43% (*E. coli* 13534), and 34% (*S. aureus*) disruption. More impressively, glass slides pre-coated with 10 mg/L NCs significantly inhibited biofilm formation, achieving 84% (*E. coli* 25922), 80% (*E. coli* 13534), and 72% (*S. aureus*) inhibition under light, demonstrating the potential for creating self-sanitizing surfaces. This study conclusively demonstrates that the optimized CdS/Ag_2_S-50% NCs are highly effective, stable, and reusable multifunctional nanomaterials with exceptional potential for visible-light-driven photocatalytic wastewater treatment and for combating bacterial infections and biofilm formation in biomedical and environmental applications.

Afreen et al. [94] successfully synthesized a reduced graphene oxide/cadmium sulfide quantum dot (rGO/CdS QD) heterostructure via a novel, environmentally friendly monowave-assisted solvothermal method, utilizing the room-temperature ionic liquid 1-ethyl-3-methylimidazolium thiocyanate ([EMIM]SCN) as a green sulfur precursor and stabilizing agent. The primary objective was to create a highly efficient, multifunctional photocatalyst capable of generating ROS for the simultaneous degradation of organic pollutants and disinfection of pathogenic bacteria in wastewater. Comprehensive characterization confirmed the successful formation of the heterostructure: XRD analysis revealed the hexagonal phase of CdS with a crystallite size of approximately 2–3 nm, uniformly dispersed on rGO sheets; TEM and SEM imaging showed spherical CdS tightly and uniformly spread over wrinkled, curled rGO sheets, which prevented CdS agglomeration and provided a high surface area (56.5 m^2^/g); Raman spectroscopy confirmed the reduction of GO to rGO with an increased D/G intensity ratio (from 0.87 to 0.97); and UV-Vis spectroscopy showed enhanced visible-light absorption with a bandgap of 2.25 eV for the heterostructure, slightly lower than pure CdS (2.36 eV). The photocatalytic performance was rigorously evaluated against a diverse range of model organic pollutants, including cationic dyes (methylene blue MB, rhodamine B RhB), anionic dyes (reactive black 39 RB39, methyl orange MO), and antibiotics (tetracycline TC, paracetamol PC) under visible-light irradiation. The rGO/CdS QD heterostructure exhibited significantly enhanced photocatalytic activity compared to pure CdS, achieving 97.1% degradation of MB within 60 min, and 90% degradation of PC within 120 min. The degradation followed pseudo-first-order kinetics, with rate constants for the heterostructure being 1.6 to 2.6 times higher than those for pure CdS. The photocatalyst showed excellent reusability, retaining 96% efficiency after four cycles. The enhanced activity was attributed to the efficient electron transfer from CdS to the highly conductive rGO sheets, which suppressed electron–hole recombination and promoted the generation of ROS. Trapping experiments using various scavengers (EDTA-2Na for h^+^, benzoquinone for O_2_^−^, and isopropyl alcohol for •OH) identified hydroxyl radicals (•OH) as the primary reactive species responsible for degradation, a finding corroborated by fluorescence spectroscopy using terephthalic acid as a probe. The antibacterial activity was assessed against Gram-positive *Staphylococcus aureus* and Gram-negative *Escherichia coli* using disk diffusion and growth kinetics assays at concentrations of 25, 50, 75, and 100 μg/mL. The rGO/CdS QD heterostructure exhibited excellent, concentration-dependent bactericidal activity, outperforming pure CdS. The MIC was found to be 16 μg/mL for *S. aureus* and 32 μg/mL for *E. coli*. SEM imaging of treated bacteria revealed severe morphological damage, including cell membrane disruption, cytoplasm leakage, and loss of cellular integrity. *S. aureus* was more susceptible than *E. coli*, attributed to the absence of an outer membrane in this Gram-positive bacterium, making it more vulnerable to ROS attack. Intracellular ROS measurement using a DCF-DA probe confirmed a concentration-dependent increase in ROS generation upon treatment with the heterostructure, and the addition of the ROS scavenger histidine significantly reduced the antibacterial effect, confirming the critical role of ROS in the disinfection mechanism. The proposed mechanism involves the rGO sheets wrapping around bacterial cell membranes, causing partial loss of integrity and facilitating the entry of the heterostructure. Upon visible-light irradiation, the generated ROS (primarily •OH) induce oxidative stress, leading to the oxidation of lipids, proteins, and nucleic acids, and ultimately cell death. This study conclusively demonstrates that the rGO/CdS QD heterostructure is a highly promising, eco-friendly, and reusable photocatalyst for the comprehensive treatment of wastewater, effectively degrading a wide spectrum of organic pollutants and inactivating pathogenic bacteria through ROS-mediated mechanisms.

Ahmad et al. [95] successfully synthesized a titanium dioxide/cadmium sulfide (TiO_2_/CdS) nanocomposite (NC) via a hydrothermal method, aiming to create a multifunctional photocatalyst capable of addressing environmental pollution and energy challenges. Comprehensive characterization confirmed the successful formation of the heterostructure: XRD analysis revealed the coexistence of tetragonal anatase TiO_2_ and hexagonal CdS phases, with crystallite sizes of 14.28 nm (TiO_2_), 67.18 nm (CdS), and 48.29 nm (TiO_2_/CdS). The NC exhibited a type IV isotherm with an H3 hysteresis loop, indicating a mesoporous structure with a BET surface area of 76.8 m^2^/g, which was higher than pristine CdS (1.7 m^2^/g) but slightly lower than TiO_2_ (80.2 m^2^/g). UV-Vis DRS analysis showed that the TiO_2_/CdS NC had a bandgap of 2.36 eV, significantly lower than TiO_2_ (3.12 eV) and comparable to CdS (2.33 eV), enabling efficient visible-light absorption. XPS analysis confirmed the presence of Ti^4+^, Ti^3+^, oxygen vacancies, and Cd^2+^ and S^2−^ states, with slight shifts in binding energies indicating strong interfacial interaction and heterojunction formation. TEM and HR-TEM imaging revealed the intimate mixing of TiO_2_ and CdS, with distinct lattice fringes corresponding to their respective planes. The photocatalytic activity was evaluated for the degradation of the persistent pharmaceutical pollutant carbamazepine (CBZ) under visible light. The TiO_2_/CdS NC exhibited superior performance, achieving 87.44% degradation of 10 mg/L CBZ within 240 min at pH 5.0 with a catalyst dose of 0.5 g/L, compared to only 15.81% for TiO_2_ and 55.46% for CdS. The degradation followed pseudo-first-order kinetics with a rate constant of 0.0082 min^−1^. Optimization studies revealed that degradation efficiency increased with catalyst dose up to 0.5 g/L, decreased at higher CBZ concentrations, and was highest at pH 5.0. Thermodynamic analysis showed the process to be endothermic (ΔH = +15.09 kJ/mol) and spontaneous (ΔG negative), with an activation energy of 17.62 kJ/mol. Radical trapping experiments identified hydroxyl radicals (•OH) as the primary reactive species, followed by holes (h^+^) and superoxide radicals (O_2_^−^). A Z-scheme mechanism was proposed, where electrons from the TiO_2_ conduction band recombine with holes from the CdS valence band, leaving highly reductive electrons in the CdS CB and highly oxidative holes in the TiO_2_ VB. The NC also demonstrated excellent stability, retaining 93.1% of its initial activity after five cycles. In addition, the NC showed promising hydrogen evolution activity (687 μmol g^−1^ h^−1^) and an apparent quantum yield of 3.8% at 420 nm. The antibacterial activity was assessed against Gram-negative *Escherichia coli* using the agar well diffusion method at concentrations of 45 and 55 μg/mL. The TiO_2_/CdS NC exhibited the highest inhibition zones of 11.05 mm and 14.13 mm at 45 and 55 μg/mL, respectively, outperforming both pristine CdS (10.69 and 13.30 mm) and TiO_2_ (10.86 and 13.99 mm), though slightly less than the standard antibiotic ciprofloxacin (15.37 mm at 55 μg/mL). The enhanced antibacterial activity was attributed to the generation of ROS under light, leading to cell membrane disruption and bacterial cell death. This study demonstrates that the TiO_2_/CdS NC is a highly promising, stable, and reusable multifunctional material with significant potential for pharmaceutical pollutant degradation, renewable hydrogen production, and bacterial disinfection in water treatment applications.

Akhtar et al. [96] successfully synthesized a p-n heterojunction CdS/CuO nanocomposite (with 5% CdS loading) via a hydrothermal method, aiming to create a multifunctional photocatalyst for environmental and energy applications. Comprehensive characterization confirmed the successful formation of the heterostructure with hexagonal CdS and monoclinic CuO phases, a bandgap of 1.66 eV enabling excellent visible-light absorption, and efficient charge separation confirmed by PL spectroscopy. The antibacterial activity was assessed against Gram-negative *Escherichia coli* using a colony count method. The CdS/CuO nanocomposite exhibited significant antibacterial potential, achieving a 70.82% reduction in bacterial cell density at a concentration of 30 mg/mL, compared to 30.88% for pristine CdS and 39.16% for pristine CuO, approaching the efficacy of the positive control ciprofloxacin (78.94%). The enhanced antibacterial activity was attributed to a combination of physical damage (membrane disruption via direct contact) and chemical damage (oxidative stress from generated ROS such as •OH, O_2_^−^, and h^+^), which attack thiol groups in enzymes and disrupt cellular physiology. This study demonstrates that the CdS/CuO heterojunction nanocomposite is a highly promising multifunctional material with significant potential for bacterial disinfection in water treatment applications.

Byeon et al. [97] synthesized pristine CdS and niobium-doped CdS (CdS-Nb1, CdS-Nb2, and CdS-Nb3 with 1–3% Nb) via a green method using Syzygium cumini seed extract. Nb doping induced a morphological shift from irregular clusters to uniform spherical and rod-like structures, increased crystallite size (24.2 to 28.05 nm), and widened the bandgap from 2.48 to 3.49 eV, enhancing charge separation. The CdS-Nb3 sample showed superior photocatalytic degradation of Alizarin Red (90.3%) and Malachite Green (98.69%) under sunlight and achieved 90.1% degradation of real industrial textile effluent. Antibacterial assays against *Staphylococcus aureus* and *Escherichia coli* revealed concentration-dependent activity, with CdS-Nb3 producing inhibition zones of 14.8 mm and 11.2 mm at 100 mg/mL, outperforming pristine CdS. The enhanced antibacterial effect was attributed to increased ROS generation, improved surface interactions, and higher surface area. CdS-Nb3 also exhibited strong antioxidant activity (92.8% DPPH scavenging). This study highlights Nb-doped CdS as a multifunctional nanomaterial for wastewater treatment and biomedical applications.

Pandeya et al. [98] successfully fabricated a flexible, self-standing CdS/TiO_2_ nanofibrous membrane (NFM) via electrospinning followed by controlled calcination with zirconium doping for mechanical integrity, and subsequent deposition of CdS using the successive ionic layer adsorption and reaction (SILAR) method. Comprehensive characterization confirmed the successful formation of the heterojunction: XRD analysis revealed the anatase phase of TiO_2_ and cubic CdS, with CdS peak intensity increasing with SILAR cycles; SEM and TEM imaging showed continuous nanofibers with uniformly attached spherical CdS NPs; and UV-Vis DRS analysis demonstrated a significant reduction in bandgap from 3.19 eV (TiO_2_) to 2.23 eV (CdS/TiO_2_), enhancing visible-light absorption. PL spectroscopy confirmed efficient charge separation in the composite. The antibacterial activity was evaluated against Gram-positive *Enterococcus faecium* and Gram-negative *Escherichia coli* using the disk diffusion method, and quantitatively against *Pseudomonas aeruginosa* via colony count. The CdS/TiO_2_ membranes exhibited significantly enhanced, dose-dependent antibacterial activity compared to the pristine TiO_2_ membrane. The zone of inhibition (ZOI) increased with CdS content, with the CdS/TZ 3 membrane (20 SILAR cycles) producing the largest inhibition zones: 12.73 mm against E. faecium and 20.36 mm against *E. coli*, the latter being comparable to the positive control Amikacin (24 mm). Quantitative suspension tests against *P. aeruginosa* showed a dramatic reduction in CFU/mL over time for the CdS/TiO_2_ membrane, far exceeding the TiO_2_ membrane. The enhanced antibacterial activity was attributed to the synergistic effects of TiO_2_ and CdS: TiO_2_ generates reactive oxygen species (ROS) such as superoxides, hydroxyl radicals, and hydrogen peroxide under UV light, which damage the bacterial cell wall, while CdS NPs release Cd^2+^ ions that interact with thiol groups in bacterial respiratory enzymes, disrupt protein layers, inhibit active transport, and interfere with DNA/RNA synthesis. The positive charge of CdS NPs also facilitates electrostatic interaction with negatively charged bacterial surfaces, further disrupting membrane integrity. This study demonstrates that the CdS/TiO_2_ Janus nanofibrous membrane is a highly promising, reusable, and mechanically flexible material with significant potential for photocatalytic wastewater treatment and antibacterial applications.

Ahmed et al. [99] synthesized CdS via pulsed laser ablation and ZnO nanoparticles via chemical precipitation, and then prepared CdS:ZnO composites (75:25, 50:50, 25:75). XRD confirmed CdS had a mixed hexagonal/cubic structure (crystallite size 54.16 nm), while ZnO showed a hexagonal wurtzite structure (29.23 nm). FESEM revealed CdS as agglomerated spheres (34.53 nm) and ZnO as dense nanopetals (51.65 nm). Bandgaps were 2.5 eV (CdS) and 4.25 eV (ZnO). Antimicrobial testing against *S. aureus*, *S. epidermidis*, *E. coli*, *Klebsiella*, and *Candida* showed inhibition zones of 15–40 mm. Pure ZnO outperformed pure CdS due to its smaller size. The composites, especially 50:50 and 25:75, showed enhanced activity, with the 50:50 composite producing a 36 mm zone against *E. coli* and 35 mm against *S. aureus*. The highest activity was against Candida (40 mm for ZnO). Enhanced efficacy was attributed to the synergistic effects of small size, high surface area, and combined Cd^2+^/Zn^2+^ ions, leading to membrane disruption, ROS generation, and the inhibition of essential biomolecules. ZnO-rich composites are promising broad-spectrum antimicrobial agents.

Al-Kilabi et al. [100] successfully synthesized polyaniline (PANI) via chemical oxidative polymerization and cadmium sulfide nanoparticles using a sol–gel method, and subsequently prepared PANI-CdS with varying CdS content (10–40 wt%) to evaluate their antibacterial efficacy. XRD analysis confirmed that PANI exhibited a semicrystalline structure with a broad peak at 25.57°, while CdS NPs showed a highly crystalline hexagonal wurtzite phase. In the nanocomposites, the characteristic CdS peaks remained, but their intensity decreased, and crystallite sizes increased from 14.27 nm (pure CdS) to 23.96 nm (20 wt% composite), indicating interaction between CdS and PANI molecular chains. FTIR spectroscopy confirmed the presence of characteristic PANI and CdS bands, with shifts in peak positions suggesting strong interfacial interactions. FESEM imaging showed uniform dispersion of CdS NPs within the porous, fibrous PANI matrix. The antibacterial activity was evaluated against Gram-positive *Staphylococcus aureus* and Gram-negative Proteus mirabilis, *Escherichia coli*, and *Klebsiella pneumoniae* using the agar well diffusion method. The nanocomposites exhibited strong, dose-dependent antibacterial activity, with inhibition zones increasing with CdS content. The PANI-CdS 40 wt% composite showed the highest activity, producing inhibition zones of 37 mm against *S. aureus*, 29 mm against *P. mirabilis*, 28 mm against *E. coli*, and 26 mm against *K. pneumoniae*, significantly outperforming pure PANI. The enhanced activity was attributed to the synergistic effect of PANI and CdS, where the porous PANI matrix facilitates bacterial adhesion, and CdS NPs generate reactive oxygen species (ROS) and release Cd^2+^ ions, disrupting cell membranes, interacting with thiol groups of enzymes, and inhibiting DNA synthesis. This study demonstrates that PANI-CdS, particularly at 40 wt% CdS, are promising, cost-effective antimicrobial agents with broad-spectrum activity.

Samadi-Maybodi et al. [101] successfully synthesized a magnetically separable Fe_3_O_4_@CdS core–shell type II nanohybrid (NH) photocatalyst, designed for enhanced photocatalytic activity and antibacterial properties with the advantage of easy recovery. Comprehensive characterization confirmed the formation of a core–shell structure: XRD analysis revealed that the CdS shell exhibited a hexagonal wurtzite structure, while the characteristic peaks of the Fe_3_O_4_ core were completely absent, indicating successful shell formation with a crystallite size of 12.6 nm. TEM imaging showed spherical particles with an average diameter of 14.2 nm. UV-Vis spectroscopy revealed a significant red shift in the absorption edge of the NHs (553 nm) compared to CdS (470 nm), with a corresponding bandgap reduction, and photoluminescence (PL) spectroscopy showed a dramatically increased exciton lifetime (128.5 ns for the NHs vs. 23.5 ns for CdS), confirming efficient type II charge separation. Quenching experiments with electron (NO_3_^−^) and hole (4-methoxyphenol) scavengers, coupled with time-resolved PL, demonstrated that in the photoexcited NHs, electrons are confined to the Fe_3_O_4_ core and holes to the CdS shell, validating the type II band alignment. The antibacterial activity was rigorously evaluated against Gram-positive Staphylococcus saprophyticus and Gram-negative *Escherichia coli* using both broth dilution (for MIC/MBC) and disk diffusion methods. The Fe_3_O_4_@CdS NHs exhibited superior, dose-dependent bactericidal activity compared to pure CdS and Fe_3_O_4_. The minimum inhibitory concentration (MIC) values were 5.0 mg/mL for *E. coli* and 4.0 mg/mL for *S. saprophyticus*, with corresponding minimum bactericidal concentration (MBC) values of 7.0 mg/mL and 6.0 mg/mL. In disk diffusion assays, the NHs produced clear inhibition zones of 9.0 mm (against *E. coli*) and 15.7 mm (against *S. saprophyticus*), outperforming pure CdS (11.0 and 14.0 mm, respectively). The nanohybrids also demonstrated excellent reusability, retaining ~94% of their antimicrobial efficacy after four cycles and one month of storage. The enhanced activity was attributed to the efficient generation of ROS, particularly •OH, as confirmed by terephthalic acid (TPA) fluorescence probing. The mechanism of bacterial cell death was elucidated through lipid peroxidation and protein carbonyl assays, which showed significant oxidative damage to cell membranes (6.2-fold increase in malondialdehyde in *E. coli*) and proteins upon exposure to the NHs under light. This study demonstrates that Fe_3_O_4_@CdS type II core–shell nanohybrids are highly promising, magnetically recoverable, and reusable photocatalysts with significantly enhanced antibacterial activity, offering a multifunctional solution for environmental remediation and biomedical applications.

Kumary et al. [102] synthesized CdS@ZIF-8 nanocomposites via “ship-in-the-bottle” (CdS within ZIF-8) and “bottle-around-the-ship” (ZIF-8 around CTAB-capped CdS) methods. Characterization confirmed successful encapsulation: FTIR showed Zn-N and Cd-S peak shifts in the ship-in-bottle composite, indicating strong interaction; XRD confirmed both phases; and BET surface area decreased significantly (from 2026 m^2^/g for ZIF-8 to 221.96 and 977.73 m^2^/g), confirming pore encapsulation. TEM revealed CdS (<5 nm) within frameworks. Antibacterial activity against *E. coli*, *S. aureus*, and *B. subtilis* showed dose-dependent inhibition zones of 12.2–35.1 mm. The ship-in-bottle composite showed highest activity against *E. coli* (22.0 mm), attributed to greater Zn^2+^ availability. The bottle-around-ship composite showed superior activity against Gram-positive *S. aureus* (26.0 mm) and *B. subtilis* (21.2 mm), likely due to CTAB disrupting fatty acid-enriched liposomes. DNA-binding studies with *E. coli* DNA revealed hyperchromic effects and red shifts, indicating intercalative binding. This study demonstrates that both encapsulation strategies yield CdS@ZIF-8 nanocomposites with enhanced, tunable antibacterial properties and DNA-binding capabilities, promising for anticancer research.

Barman et al. [103] synthesized Fe-doped ZnO/CdS (0.5–2.0 wt% Fe) via a chemical method. XRD with Rietveld refinement confirmed a hexagonal wurtzite structure with Fe^3+^ ions substituting into Cd/Zn vacancies, yielding crystallite sizes of 8–12 nm. TEM revealed monodispersed, spherical/hexagonal particles (~40 nm), while SEM showed sizes of 22–42 nm. EDX confirmed elemental purity. UV-Vis spectroscopy showed bandgap variation from 3.83 to 4.1 eV, attributed to lattice mismatch from Fe^2+^ incorporation. Antibacterial activity against *E. coli*, *K. pneumoniae*, *P. aeruginosa*, and *S. aureus* was evaluated via well diffusion (50–100 μL). The composites exhibited dose-dependent activity, with inhibition zones of 3–20 mm. At 100 μL, zones were 18 mm (*E. coli*), 20 mm (*K. pneumoniae*), 17 mm (*P. aeruginosa*), and 20 mm (*S. aureus*), compared to tetracycline (31–35 mm). Enhanced activity was attributed to ROS generation, leading to oxidative stress, membrane damage, and cell death. This study highlights Fe-doped ZnO/CdS as promising antimicrobial agents for water purification and biomedical applications.

Sundaria et al. [104] synthesized star-shaped and spherical copper-doped cadmium sulfide (Cu:CdS) nanoparticles via a wet chemical method by varying reaction temperature. Lowering the temperature reduced particle kinetic energy, minimizing collisions and preserving sharp edges, resulting in star-shaped nanoparticles, while room-temperature synthesis produced spherical particles. UV-Vis spectroscopy showed absorption peaks at ~310 nm (spherical) and ~340 nm (star-shaped), with corresponding photoluminescence emissions at 410 nm and 415 nm, confirming successful Cu doping. SEM imaging clearly revealed the distinct morphologies. The antibacterial activity was evaluated against Serratia marcescens and antibiotic-resistant *Streptococcus* strains using the disk diffusion method. Against S. marcescens, star-shaped Cu:CdS produced a larger inhibition zone (10 mm) compared to spherical nanoparticles (7 mm) at 1 mg/mL. Against ampicillin-resistant *Streptococcus*, star-shaped nanoparticles showed a 16 mm zone versus 12 mm for spherical at 8 mg/mL. Against streptomycin-resistant *Streptococcus*, star-shaped nanoparticles produced a 19 mm zone, significantly larger than the 14 mm zone from spherical nanoparticles. The enhanced antibacterial activity of star-shaped nanoparticles was attributed to their sharp edges, which can physically disrupt bacterial plasma membranes more effectively than spherical particles, in addition to the inherent toxicity of Cu:CdS. This study demonstrates that nanoparticle morphology significantly influences antibacterial efficacy, and star-shaped Cu:CdS represents a promising agent against drug-resistant bacteria.

**Figure 7:**
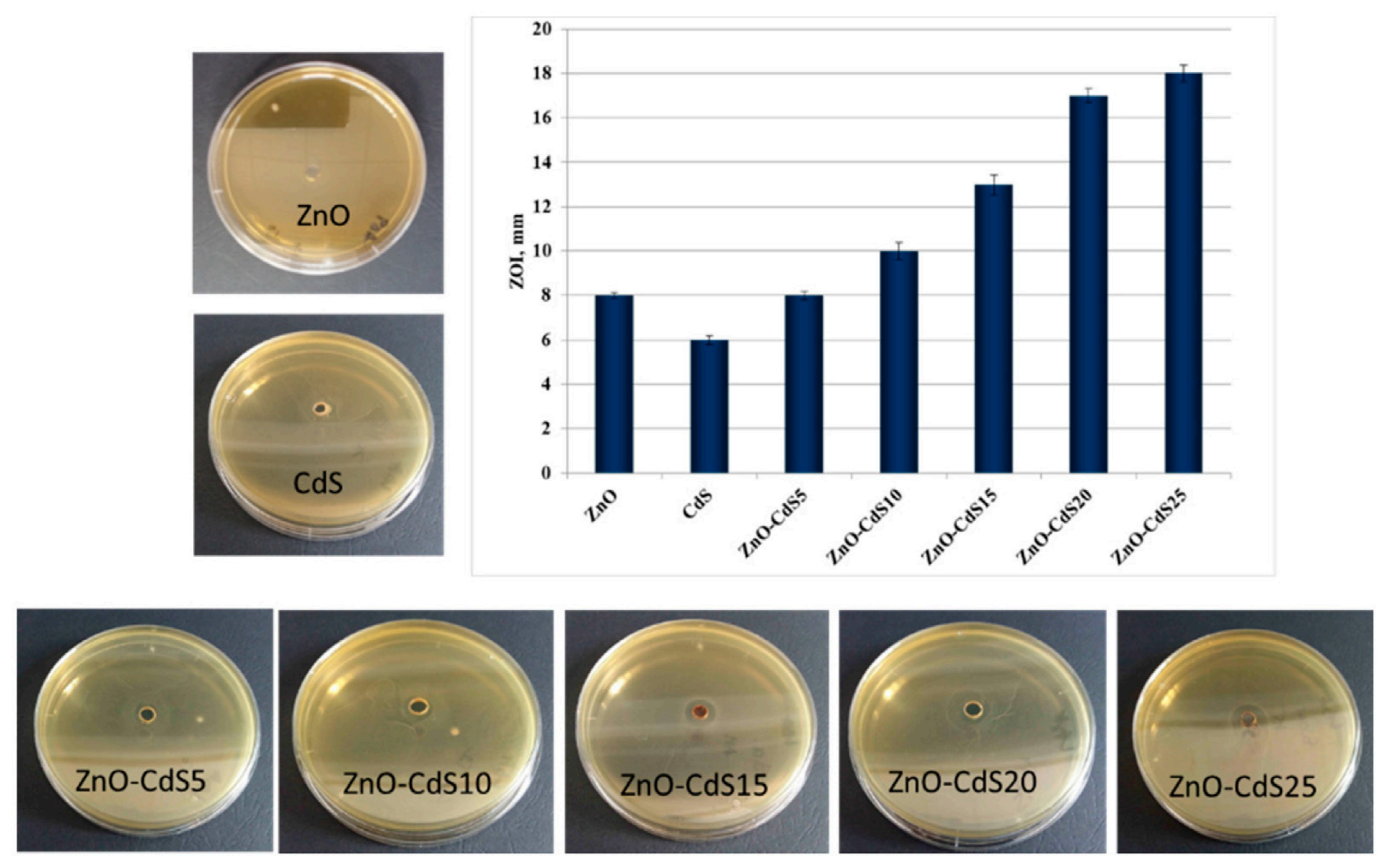
The antibacterial activity of ZnO, CdS, and ZnO–CdS powders toward *Escherichia coli* ATCC 8738, showing the respective zones of inhibition. Reprinted with permission from [105] (CC BY license).

Zgura et al. [105] synthesized ZnO-CdS composite powders with varying CdS concentrations (5–25 mM) via chemical precipitation. XRD confirmed hexagonal ZnO and cubic CdS phases, with CdS peak intensity increasing with concentration. XPS confirmed Zn^2+^, O^2−^, Cd^2+^, and S^2−^ states and oxygen vacancies (Figure 7). SEM revealed flower-like structures tunable by CdS content. Photocatalytic tests showed most composites outperformed pristine ZnO in methylene blue degradation, attributed to efficient charge separation and hydroxyl radical generation. Antibacterial activity against *E. coli* was dose-dependent, with inhibition zones increasing from ~5 mm to ~20 mm as CdS content rose, following a sigmoidal trend. Enhanced activity was attributed to ZnO improving composite solubility and cellular uptake, the flower-like morphology aiding membrane disruption, and released Zn^2+^/Cd^2+^ ions electrostatically interacting with bacterial membranes. Antioxidant capacity (35–52%) increased with CdS content. Cytotoxicity tests on human fibroblast BJ cells showed composites with 5 and 10 mM CdS were non-toxic below 0.05 mg/mL, while higher CdS concentrations exhibited significant toxicity. This study demonstrates that ZnO-CdS composites, particularly with optimized CdS content, are promising for photocatalytic and antibacterial applications but require careful balancing of efficacy and biocompatibility.

Stavitskaya et al. [106] synthesized CdS as QDs on various mesoporous aluminosilicate and silicate carriers, including halloysite nanotubes (HNTs), MCM-41, SBA-15, and a novel hierarchical MCM-41/HNTs composite, to evaluate their photocatalytic antibacterial activity under visible light. Characterization confirmed the successful formation of CdS with sizes below 10 nm and Cd content of 5–7 wt%. Bandgap energies varied from 2.36 to 2.58 eV, indicating quantum confinement effects. Antibacterial activity was assessed against antibiotic-resistant *Staphylococcus aureus* (Gram-positive) and *Escherichia coli* (Gram-negative). In the dark, negligible activity was observed. Under visible light, all composites exhibited significant, dose-dependent antibacterial effects. The hierarchical CdS/MCM-41/HNTs composite demonstrated the highest efficacy, achieving 100% growth inhibition of *S. aureus* at 125 μg/mL and a bacteriostatic effect at 63 μg/mL. Against *E. coli*, CdS/MCM-41/HNTs and CdS/SBA-15-SH achieved 100% inhibition at 1000 μg/mL. The enhanced activity was attributed to efficient charge separation, high surface area, and optimal CdS dispersion, promoting ROS generation. Cytotoxicity tests on A549 human lung cancer cells showed that CdS/MCM-41/HNTs was the least toxic, with acceptable biocompatibility at lower concentrations. This study highlights the critical role of the carrier in modulating both antibacterial efficacy and eukaryotic toxicity, identifying CdS/MCM-41/HNTs as the most promising candidate for visible-light-driven photocatalytic antibacterial applications.

Pitchaimani et al. [107] deposited undoped CdS, Co-doped CdS (Cd_0.98_Co_0.02_S), and Mn, Co dual-doped CdS (Cd_0.96_Mn_0.02_Co_0.02_S) thin films on glass substrates via chemical bath deposition to investigate their structural, optical, and antibacterial properties. XRD analysis confirmed the cubic phase for all films, with peak shifts towards higher angles and decreased crystallite size upon doping, indicating successful substitution of Cd^2+^ by smaller Co^2+^ (0.74 Å) and Mn^2+^ (0.66 Å) ions. Film thickness increased from 280 nm (CdS) to 381 nm (dual-doped). SEM imaging revealed morphological evolution from irregular clusters (CdS) to mixed spherical/rod-like (Co-doped) and compact plate-like structures (Mn,Co dual-doped). UV-Vis spectroscopy showed a blue shift in the absorption edge, with bandgap increasing from 2.44 eV (CdS) to 2.57 eV (dual-doped), attributed to quantum confinement. Photoluminescence spectra exhibited a strong green emission (538–547 nm) from sulfur vacancy-related surface states. Antibacterial activity against *Escherichia coli* and *Staphylococcus aureus* was evaluated using the disk diffusion method. The Mn, Co dual-doped CdS (Cd_0.96_Mn_0.02_Co_0.02_S) exhibited the highest antibacterial efficacy, producing inhibition zones of ~32 mm against *E. coli* and ~31 mm against *S. aureus*. This enhanced activity was attributed to the increased surface area from reduced crystallite size and the favorable plate-like morphology facilitating bacterial cell wall penetration. This study demonstrates that Mn, Co dual-doping significantly enhances the antibacterial properties of CdS thin films.

Selvan et al. [108] deposited undoped CdS, Zn-doped (6 at.% Zn), and (Zn + F) doubly doped CdS thin films with varying F concentrations (1–3 wt%) on glass substrates at 400 °C using a low-cost spray technique. XRD confirmed that all films were polycrystalline hexagonal with strong (002) orientation. Doping shifted peaks to higher angles, indicating lattice contraction due to smaller Zn^2+^ (0.74 Å) and F^−^ (1.33 Å) substituting for Cd^2+^ (0.97 Å) and S^2−^ (1.84 Å). The 2 wt% F film exhibited highest crystallinity with increased crystallite size (25.43 nm) and reduced strain. SEM revealed morphological evolution from nano-needled grains (1 wt% F) to honeycomb structures (2 wt% F). EDX confirmed Zn and F incorporation and increased sulfur vacancies. Optical studies showed bandgap widening (2.45 to 2.72 eV) attributed to the Moss–Burstein effect. Antibacterial activity against *E. coli* via disk diffusion showed inhibition zones of 25–40 mm, attributed to ROS generation from sulfur vacancy-related defects producing H_2_O_2_ that disrupts bacterial membranes. This study highlights (Zn + F) doubly doped CdS thin films as promising antimicrobial agents.

Patel et al. [109] synthesized pure and manganese-incorporated cadmium sulfide (Mn-CdS) nanoparticles with varying Mn concentrations (10, 15, and 20 mol%) via a chemical co-precipitation method using PVP as a capping agent. UV-Vis-NIR spectroscopy revealed a blue shift in the absorption edge compared to bulk CdS, with the bandgap decreasing from 3.23 eV (pure CdS) to 3.13 eV (20% Mn-CdS), attributed to sp-d exchange interactions between Mn^2+^ ions and CdS valence electrons. FTIR confirmed Cd-S and Mn-S bonding. Photoluminescence spectra showed two emission peaks at 397 nm (band edge recombination) and ~541 nm (surface trap-induced emission), with intensity enhanced by Mn incorporation. TGA indicated that Mn incorporation decreased thermal stability, with weight loss increasing with Mn concentration. Antibacterial activity was evaluated against Gram-positive (*Staphylococcus aureus*, *Bacillus subtilis*) and Gram-negative (*Escherichia coli*, *Salmonella paratyphi* A) bacteria using the agar plating–spot inoculation method at concentrations of 1–5 mg/mL. Pure CdS showed antagonism only against Gram-positive bacteria. In contrast, Mn-incorporated CdS exhibited broad-spectrum activity against both Gram-positive and Gram-negative strains, particularly at higher Mn concentrations and sample concentrations (e.g., 20% Mn-CdS at 5 mg/mL), with inhibition zones of approximately 7–11 mm. The enhanced activity was attributed to the toxicity of Mn^2+^ ions, which disrupt Fe-S cluster assembly in bacterial enzymes, leading to inactivation. This study demonstrates that Mn incorporation effectively tunes CdS into broad-spectrum antimicrobial agents.

Wali et al. [110] synthesized cadmium sulfide nanoparticles via a hydrothermal method at different pH values (2, 5, 7, 9, and 11), followed by calcination at 500 °C, and subsequently prepared gold-decorated CdS (Au/CdS) using a photo-deposition method. XRD and FESEM analysis confirmed the formation of nanoparticles with sizes ranging from 20 to 100 nm. The antibacterial activity was evaluated against Proteus mirabilis using the agar well diffusion method at concentrations ranging from 400 to 4000 μg/mL. MIC and MBC were determined via broth dilution. CdS alone showed limited activity, with MIC values of 1000–2000 μg/mL and MBC values of 2000–4000 μg/mL, depending on synthesis pH. In contrast, Au/CdS exhibited significantly enhanced antibacterial efficacy, with MIC values of 800–1000 μg/mL and MBC values of 1000–4000 μg/mL. The largest inhibition zones (up to 15.33 mm) were observed for Au/CdS synthesized at pH 9 and 11 at 4000 μg/mL. The enhanced activity was attributed to the synergistic effect of gold, which increases surface plasmon resonance and improves nanoparticle–bacteria interaction.

Ahmed et al. [111] synthesized bare CdS and Ni-Cu co-incorporated CdS solid solutions (Cd_80_Ni_10_Cu_10_S, Cd_60_Ni_20_Cu_20_S, and Cd_40_Ni_30_Cu_30_S) via chemical co-precipitation. pXRD confirmed the cubic structure of all samples, with successful Ni^2+^ and Cu^2+^ incorporation causing slight peak shifts and decreased crystallinity due to their smaller ionic radii. Crystallite sizes ranged from 0.80 to 1.56 nm. FTIR confirmed Cd-S stretching (~648 cm^−1^) and successful metal incorporation. SEM revealed spherical morphology with agglomeration. UV-Vis spectroscopy showed tunable bandgaps, with Cd_60_Ni_20_Cu_20_S exhibiting the lowest bandgap (2.06 eV), making it optimal for applications. Photocatalytic degradation of Congo Red dye under visible light reached 92% with Cd_60_Ni_20_Cu_20_S, outperforming bare CdS (85%), attributed to enhanced light absorption and defect-mediated charge separation. Antibacterial activity against *E. coli* and *Lactobacillus* was assessed via agar well diffusion. Cd_60_Ni_20_Cu_20_S exhibited significant, concentration-dependent inhibition, with zones of 22–26 mm, compared to negligible activity for bare CdS. Antifungal activity against *Aspergillus niger* and *Aspergillus flavus* showed inhibition zones of 24–28 mm for Cd_60_Ni_20_Cu_20_S, comparable to the standard Nystatin. Molecular docking studies revealed good binding affinities of the doped nanoparticles with target enzymes (FabH, β-lactamase, glucoamylase, urate oxidase), supporting the experimental antimicrobial results. This study demonstrates that Ni-Cu co-doping significantly enhances the photocatalytic, antibacterial, and antifungal properties of CdS.

Suryawanshi et al. [112] synthesized CdS as nanoparticles capped with varying concentrations of cetyltrimethylammonium bromide (CTAB) (1%, 2%, and 3%) using a novel ultrasonication technique. XRD analysis confirmed the hexagonal phase of CdS, with crystallite sizes increasing from 3.65 to 4.31 nm as CTAB concentration increased. SEM revealed uniform surface morphology with particle sizes of 18.56–25.61 nm, while TEM showed individual particle sizes of 3.13–4.45 nm, confirming nanoparticle formation. UV-Vis spectroscopy showed bandgap values decreasing from 1.62 to 1.57 eV with increasing CTAB, lower than bulk CdS (2.42 eV), indicating quantum confinement effects. FTIR confirmed the presence of CTAB on the nanoparticle surface. TGA revealed weight loss of 16.64–25.57% up to 800 °C, attributed to water loss and CTAB degradation. The antibacterial activity was evaluated against Methicillin-Resistant *Staphylococcus aureus* (MRSA, ATCC 4330) using disk diffusion and minimum inhibitory concentration (MIC) assays. The CdS exhibited moderate antibacterial activity, with MIC values > 256 μg/mL for the 2% and 3% CTAB-capped samples, while the 1% CTAB-capped sample was inactive. Zone of inhibition measurements at 1 μg/mL concentration showed values of 8.46 mm and 8.57 mm for the 2% and 3% CTAB samples, respectively, compared to 8.74 mm for the standard antibiotic sultamicillin. The antibacterial mechanism was attributed to the small particle size facilitating cell membrane penetration and interaction with cellular components. This study demonstrates that CTAB-capped CdS have potential as moderate antibacterial agents against drug-resistant MRSA strains.

Nadeem et al. [113] successfully fabricated a direct Z-scheme ZnNiNdO/CdS heterojunction nanocomposite via co-precipitation and ultra-sonication methods, designed for enhanced photocatalytic and antibacterial applications. XRD confirmed the hexagonal wurtzite structure of ZnO and the presence of CdS peaks, indicating successful heterojunction formation. Co-doping with Ni and Nd reduced the ZnO bandgap from 3.37 to 2.9 eV, which was further reduced to 2.6 eV upon coupling with CdS, enabling efficient visible-light absorption. PL spectroscopy revealed reduced electron–hole recombination in the heterojunction. FE-SEM showed a connected network morphology beneficial for surface interactions. The photocatalytic activity was evaluated against multiple organic pollutants under sunlight. The ZnNiNdO/CdS nanocomposite exhibited exceptional degradation efficiencies: 99.7% for methylene blue (MB), 49% for p-nitroaniline, 96.6% for methyl orange (MO), 98.6% for methyl red (MR), and 98.6% for rhodamine B (RhB) within 50 min. The rate constant for MB degradation was 0.1243 min^−1^, significantly higher than pristine and singly doped counterparts. The catalyst demonstrated excellent stability, retaining ~95% activity after seven cycles. Radical trapping experiments confirmed superoxide (O_2_^−^) and hydroxyl (•OH) radicals as the primary reactive species. The antibacterial activity was assessed against human pathogenic bacteria, including *Staphylococcus aureus*, *Escherichia coli*, *Proteus vulgaris*, and *Pseudomonas aeruginosa*, using the disk diffusion method at concentrations of 2, 4, 6, and 8 μg/mL. The ZnNiNdO/CdS nanocomposite exhibited potent, concentration-dependent antibacterial activity, producing maximum inhibition zones of 30 mm (*S. aureus*), 33 mm (*E. coli*), 27 mm (*P. vulgaris*), and 31 mm (*P. aeruginosa*), outperforming both the individual components and the standard antibiotic ciprofloxacin. The enhanced activity was attributed to the Z-scheme heterojunction facilitating efficient charge separation, leading to enhanced generation of ROS (O_2_^−^ and •OH), which cause oxidative stress, membrane damage, and cell death. This study demonstrates that energy-level-matched Z-scheme heterojunctions are a powerful strategy for developing multifunctional nanomaterials for environmental remediation and antimicrobial applications.

Reena et al. [114] synthesized DNA-capped cadmium sulfide nanoparticles (D-CdS) via a co-precipitation method using herring sperm DNA as a capping agent to enhance biocompatibility and dispersion. Characterization confirmed successful formation: UV-Vis spectroscopy showed an absorption edge at 480 nm with a calculated bandgap of 2.58 eV, indicating quantum confinement. XRD revealed a cubic crystalline structure with crystallite sizes of 1.5–2.7 nm. TEM and SEM showed spherical morphology with an average diameter of 10 nm for D-CdS, compared to >50 nm for uncapped CdS. PL spectroscopy exhibited enhanced fluorescence with peaks at 486 nm and 541 nm, blue-shifted relative to uncapped CdS. Photocatalytic studies showed D-CdS degraded 91% of methylene blue and 64% of rhodamine 6G under UV irradiation within 120 min. Antibacterial activity was evaluated against *Pseudomonas aeruginosa* (Gram-negative), *Staphylococcus aureus* (Gram-positive), and *Streptococcus* mutans (Gram-positive) using the disk diffusion method at 1000 μg/mL. D-CdS exhibited inhibition zones of 24 mm against *P. aeruginosa*, 20 mm against *S. aureus*, and 17 mm against *S. mutans*, outperforming uncapped CdS (12, 12, and 11 mm, respectively). The enhanced activity was attributed to the smaller particle size and DNA capping facilitating interaction with bacterial membranes, particularly against Gram-negative bacteria due to lipopolysaccharide–DNA interactions. Cytotoxicity assays on HeLa cells showed D-CdS had an LC_50_ of 8 μg/mL, demonstrating improved biocompatibility over uncapped CdS (LC_50_ = 2.4 μg/mL). Fluorescence imaging confirmed D-CdS as a promising candidate for bioimaging applications. This study highlights DNA-capped CdS as a multifunctional nanomaterial with enhanced photocatalytic, antibacterial, and bioimaging properties.

Golabiazar et al. [115] successfully synthesized CdS and CdS/Fe_3_O_4_ nanocomposites (NCs) using an eco-friendly green approach with hawthorn (*Crataegus* spp.) leaf extract, which served as a natural reducing, stabilizing, and capping agent. Comprehensive characterization confirmed the successful formation of the nanomaterials: XRD analysis revealed the coexistence of hexagonal CdS and cubic Fe_3_O_4_ phases in the nanocomposite, with an average crystallite size of 21.2 nm (Scherrer method) and 34.6 nm (Williamson–Hall method), indicating the presence of tensile strain. FESEM and TEM imaging showed spherical, aggregated particles with average sizes of 16.6 nm (CdS) and 28.13 nm (CdS/Fe_3_O_4_), with TEM revealing a thin organic layer from the plant extract on the NC surface. FTIR spectroscopy confirmed the presence of phytochemicals on the nanoparticle surface and metal–sulfur (Cd-S) and metal–oxygen (Fe-O) bonds. EDX analysis confirmed the elemental composition with a CdS:Fe_3_O_4_ mole ratio of approximately 1:2. The photocatalytic activity was evaluated for the degradation of methyl orange (MO) dye under UV irradiation. The CdS/Fe_3_O_4_ NCs exhibited excellent pH-dependent photocatalytic performance, with degradation efficiency increasing with pH from 3 to 10, achieving the highest rate at pH 10. The degradation followed pseudo-first-order kinetics, with rate constants increasing from 0.090 min^−1^ (pH 3) to 0.268 min^−1^ (pH 10). The enhanced activity was attributed to the synergistic effect of the CdS-Fe_3_O_4_ heterojunction, which facilitates efficient charge separation and generation of ROS, particularly •OH, and the enhanced electrostatic attraction between the positively charged NC surface and anionic MO molecules at basic pH. The antibacterial activity was evaluated against a panel of pathogenic bacteria, including Gram-positive Enterobacter aeruginosa and Gram-negative *Escherichia coli*, *Pseudomonas aeruginosa*, and *Klebsiella pneumoniae*, using the well diffusion method at concentrations of 0.001–0.025 g/mL. The CdS/Fe_3_O_4_ NCs exhibited concentration-dependent antibacterial activity, with inhibition zones ranging from 17 to 26 mm. The highest activity was observed against *K. pneumoniae* (26 mm at 0.025 g/mL), followed by *P. aeruginosa* (22 mm), *E. coli* (19 mm), and *E. aeruginosa* (25 mm). The NCs significantly outperformed the hawthorn extract control, demonstrating the enhanced bioactivity of the nanomaterial. The antibacterial mechanism was attributed to the generation of ROS (superoxide radicals, hydroxyl radicals, hydrogen peroxide) from the NCs, leading to oxidative stress, damage to proteins and DNA, and ultimately cell death. This study demonstrates that green-synthesized CdS/Fe_3_O_4_ nanocomposites are promising multifunctional materials for photocatalytic wastewater treatment and antibacterial applications, with the added advantage of magnetic separability.

Abdelmoneim et al. [116] successfully synthesized a multifunctional CdS/TiO_2_ nanocomposite by decorating hydrothermally prepared TiO_2_ nanowires with CdS via a wet impregnation method. Comprehensive characterization confirmed the successful formation of the heterostructure: XRD revealed the anatase phase of TiO_2_ and cubic phase of CdS, with peak intensities confirming CdS loading. TEM and SEM imaging showed TiO_2_ nanowires (8–15 nm diameter, 42–80 nm length) densely decorated with spherical CdS (11–17 nm). XPS confirmed the presence of Ti^4+^, O^2−^, Cd^2+^, and S^2−^, with binding energy shifts indicating strong interfacial interaction. UV-Vis spectroscopy showed that the nanocomposite had a bandgap of 3.02 eV, between those of TiO_2_ (3.3 eV) and CdS (2.46 eV), enabling enhanced visible-light absorption. The photocatalytic activity was evaluated for MB dye degradation under UV-Vis light. The CdS/TiO_2_ nanocomposite exhibited superior performance, achieving 95.46% degradation within 120 min at optimal conditions (pH 11, 1 g/L catalyst, 10 ppm MB), significantly outperforming pure TiO_2_ nanowires (88.5%) and CdS (54.8%). The enhanced activity was attributed to the efficient heterojunction facilitating charge separation and generation of ROS. Radical trapping experiments confirmed •OH as the primary species. The nanocomposite demonstrated excellent reusability, retaining high activity after five cycles. Antibacterial activity was assessed against Gram-positive (*Bacillus subtilis*, *Staphylococcus aureus*) and Gram-negative (*Escherichia coli*, *Pseudomonas aeruginosa*) bacteria using the diffusion method at concentrations of 0.156–5 mg. The CdS/TiO_2_ nanocomposite exhibited the highest, dose-dependent activity, with inhibition zones up to 32.6 mm against *B. subtilis* at 5 mg, outperforming both pure components. The MIC values were 0.312 mg for *B. subtilis*, 0.625 mg for *S. aureus*, and 1.25 mg for *E. coli* and *P. aeruginosa*. The enhanced antibacterial activity was attributed to membrane disruption by the nanocomposite and ROS generation. Notably, the nanocomposite also exhibited potent larvicidal activity against Culex pipiens larvae, achieving 99.6% mortality at 1 mg after 72 h, the first report of such activity for CdS/TiO_2_. This study demonstrates that the CdS/TiO_2_ nanocomposite is a highly promising multifunctional material for photocatalytic wastewater treatment, antibacterial applications, and mosquito vector control.

Dey et al. [117] synthesized ligand-free CdS and silver-doped CdS (Ag/CdS) nanocrystals via a chemical method to investigate their antibacterial activity against Gram-positive *Staphylococcus aureus* and Gram-negative *Escherichia coli*. TEM and SEM analysis revealed spherical morphology with average particle sizes of 5 nm for CdS and 7 nm for Ag/CdS. XRD confirmed both nanocrystals possessed a polycrystalline cubic zinc blende structure, with a slight shift in peaks for Ag/CdS indicating successful Ag incorporation. EDX confirmed elemental composition with 15.92 wt% Ag in the doped sample. Antibacterial activity was evaluated using the Kirby-Bauer disk diffusion method at concentrations of 10, 50, 100, 150, and 200 μg/mL. Both CdS and Ag/CdS nanocrystals showed no activity against *E. coli* at these concentrations, attributed to the rigid outer membrane and lipopolysaccharide layer of Gram-negative bacteria acting as a permeability barrier. In contrast, both nanocrystals exhibited dose-dependent activity against *S. aureus*. For CdS, inhibition zones increased from 6 mm (10 μg/mL) to 12 mm (200 μg/mL). Ag/CdS showed enhanced activity, with zones increasing from 7 mm (10 μg/mL) to 16 mm (200 μg/mL). The enhanced activity of Ag/CdS was attributed to the release of Ag^+^ ions, which interact with thiol groups in bacterial enzymes and proteins, causing dysfunction. The activity against *S. aureus* was facilitated by the porous peptidoglycan layer of Gram-positive bacteria, allowing for nanocrystal penetration. This study demonstrates that Ag doping significantly enhances the antibacterial efficacy of CdS nanocrystals against Gram-positive bacteria at low concentrations.

Gupta et al. [118] successfully synthesized a pectin–cadmium sulfide nanocomposite (Pc/CSNC) via an environmentally friendly aqueous phase co-precipitation method at 60 °C, using pectin as a natural coupling and encapsulating agent. FTIR spectroscopy confirmed the incorporation of pectin through characteristic carboxyl and methylene peaks, and the presence of Cd-S stretching vibrations at 616 cm^−1^ and 760 cm^−1^. XRD analysis revealed a cubic crystalline structure for CdS with broad peaks at 2θ = 25.91°, 44.24°, and 52.20°, corresponding to the (111), (220), and (311) planes, with an average crystallite size of 3.2 nm calculated via the Debye–Scherrer equation. TEM imaging showed spherical nanoparticles well dispersed within the pectin matrix, with particle sizes ranging from 4 to 10 nm, consistent with XRD results. SEM analysis revealed a porous, irregular morphology. TGA showed that the nanocomposite was thermally stable, with major weight loss (28%) between 175 and 520 °C corresponding to pectin decomposition. The photocatalytic activity was evaluated for MB dye degradation under sunlight and sodium lamp irradiation. Pc/CSNC achieved 95.5% degradation under sunlight and 88.9% under sodium lamp within 6 h, following pseudo-first-order kinetics with rate constants of 0.00413 min^−1^ (sunlight) and 0.0033 min^−1^ (sodium lamp). The enhanced activity was attributed to efficient electron–hole pair generation and reduced recombination. Antibacterial activity against Gram-negative *Escherichia coli* was assessed via growth curve and well diffusion methods at 50 and 100 μg/mL. The nanocomposite exhibited dose-dependent antibacterial activity, with inhibition zones of 14 mm (50 μg/mL) and 27 mm (100 μg/mL). Growth curve analysis confirmed reduced bacterial proliferation over 12 h. The mechanism was attributed to the release of Cd^2+^ ions from the nanocomposite, which bind to thiol groups in bacterial membrane proteins, disrupting membrane permeability, inhibiting enzyme activity, and ultimately leading to cell death. This study demonstrates that pectin-capped CdS are promising, eco-friendly materials for photocatalytic wastewater treatment and antibacterial applications.

Gao et al. [119] successfully synthesized graphene oxide–cadmium sulfide (GO-CdS) composites via a novel two-phase mixing method, where CdS (5–6 nm) was uniformly self-assembled onto GO sheets at a water/toluene interface. Characterization confirmed strong interfacial interactions and uniform deposition, which minimized CdS aggregation and facilitated charge transfer. Under visible light, GO-CdS exhibited significantly enhanced photocatalytic degradation of organic pollutants (AO7, MB, RhB) and photoreduction of Cr^6+^ compared to pure CdS, with over 80% AO7 degradation in 60 min. The composite also showed excellent photostability, leaching only 3.5 wt% Cd^2+^ versus 38.6% for pure CdS. Crucially, this study reports for the first time the disinfection activity of GO-CdS composites. Under visible-light irradiation, GO-CdS achieved nearly 100% inactivation of both Gram-negative *Escherichia coli* and Gram-positive *Bacillus subtilis* within 24 h, significantly outperforming pure CdS (55% kill). The enhanced activity was attributed to efficient charge transfer from CdS to GO, reducing electron–hole recombination and promoting hydroxyl radical (•OH) generation, confirmed by transient photocurrent measurements, ESR spectroscopy, and radical scavenger (DMSO) tests. This study demonstrates GO-CdS as a highly promising, stable photocatalyst for water purification and disinfection.

Gao et al. [120] successfully synthesized novel hierarchical “spindle-like” TiO_2_/CdS composites by growing CdS nanocrystals uniformly on nanoporous TiO_2_ mesocrystals via a two-step hydrothermal and hot-injection method, using L-cysteine as a bifunctional linker to ensure excellent contact and uniform distribution. Characterization confirmed the anatase TiO_2_ mesocrystals (200–400 nm) were uniformly decorated with CdS (5–8 nm) without aggregation. The TiO_2_/CdS-2 composite (optimal Ti/Cd mass ratio of 2) exhibited the highest specific surface area (91.7 m^2^/g) and enhanced visible-light absorption (bandgap ~2.4 eV). Under visible light, TiO_2_/CdS-2 achieved 95% degradation of Rhodamine B within 60 min, with a rate constant 10 times higher than that of pure TiO_2_ and 3.5 times higher than that of pure CdS. Total organic carbon (TOC) analysis confirmed effective mineralization. Crucially, the composite demonstrated exceptional antibacterial activity against *Escherichia coli* (Gram-negative) under visible light. At 5 mg, in a 50 mL bacterial solution (10^8^ CFU/mL), TiO_2_/CdS-2 achieved >99.9% inactivation within 10 min, significantly outperforming pure CdS (94% kill) and TiO_2_ (22% kill). The mechanism was attributed to efficient charge transfer from CdS to TiO_2_ via the L-cysteine linker, reducing electron–hole recombination and generating reactive oxygen species (•OH and O_2_^−^), which damage bacterial cells. This study highlights the importance of optimal CdS loading and intimate heterojunction contact for maximizing photocatalytic and antibacterial performance.

Jana et al. [121] successfully synthesized CdS/ZnO nanocomposites with varied morphologies by engineering the precursor molar ratio (1:1, 1:2, and 1:3 CdS:ZnO) via a facile wet chemical method. Structural and morphological characterization revealed that the CdS/ZnO 1:1 (CZ1:1) composite formed a compact flower-like structure with well-integrated CdS-ZnO interfaces, while CZ1:2 exhibited loosely bound ball-like structures, and CZ1:3 showed agglomerated flower-like structures with some dissociation. XRD confirmed the presence of cubic CdS and hexagonal ZnO phases. Photocatalytic studies under visible light showed that CZ1:1 exhibited the highest activity, degrading 90% of Rhodamine B within 190 min, with a rate constant of 0.01333 min^−1^, significantly outperforming CZ1:2 and CZ1:3. This enhanced activity was attributed to the larger interface area and efficient charge separation in the compact flower-like structure. Antibacterial activity was evaluated against Gram-positive *Staphylococcus aureus* and Gram-negative *Escherichia coli* and *Klebsiella pneumoniae* using the well diffusion method in the dark. Bare CdS and ZnO showed no activity. All nanocomposites exhibited significant, morphology-dependent antibacterial activity, with inhibition zones increasing with ZnO content. The CZ1:3 composite (1:3 ratio) showed the highest activity, producing inhibition zones of 22 mm against *S. aureus*, 14 mm against *E. coli*, and 13 mm against *K. pneumoniae*, compared to the positive control Norfloxacin (30, 24, and 23 mm, respectively). The enhanced activity was attributed to Zn^2+^ ion release and superoxide anion generation from ZnO, disrupting bacterial membranes. This study demonstrates that CdS/ZnO nanocomposites can be structurally tuned for multifunctional applications, with compact flower-like structures favoring photocatalysis and higher ZnO content favoring antibacterial activity.

Zirak et al. [122] synthesized vertically aligned ZnO@CdS nanorod heterostructure films via a three-step sol–gel/hydrothermal/SILAR method, with varying CdS nanoparticle loadings (10, 20, and 30 SILAR cycles) to investigate their visible-light-induced antibacterial activity against *Escherichia coli*. Characterization confirmed that ZnO nanorods (~50 nm diameter) were uniformly coated with CdS (~5 nm), forming a type II heterojunction with bandgaps of 2.5–2.6 eV, enabling visible-light absorption. XPS analysis revealed that the ZnO nanorods synthesized hydrothermally contained significant surface OH groups, which decreased with increasing CdS loading. Antibacterial tests showed that neither bare ZnO nanorods under visible light nor the heterostructures in the dark exhibited significant activity. However, under visible-light irradiation, the ZnO@CdS nanorods with optimal CdS loading (10 cycles, effective CdS thickness < 15 nm) achieved complete (100%) inactivation of *E. coli* within 24 h. Higher CdS loadings (20 and 30 cycles) resulted in reduced activity (77% and 63% kill, respectively), attributed to the depletion of surface OH groups essential for hydroxyl radical (•OH) generation. The proposed mechanism involves photoexcited electron transfer from CdS to ZnO, promoting charge separation and •OH production from surface OH groups, leading to bacterial cell damage. Water contact angle measurements confirmed that higher surface OH content correlated with increased hydrophilicity and enhanced antibacterial activity. This study demonstrates that optimizing CdS shell thickness and preserving surface hydroxyl groups are critical for maximizing the visible-light photocatalytic antibacterial performance of ZnO@CdS heterostructures.

Narasimman et al. [123] successfully deposited undoped and zirconium-doped cadmium sulfide (CdS:Zr) thin films with varying Zr concentrations (0–4 wt.%) on glass substrates using a low-cost spray technique with a perfume atomizer. XRD analysis confirmed that all films were polycrystalline with a hexagonal wurtzite structure and a strong (002) preferential orientation. Zr doping caused a shift in diffraction peaks to higher angles, indicating lattice contraction due to the substitution of smaller Zr^4+^ ions (0.74 Å) for Cd^2+^ ions (0.97 Å). Crystallite size decreased from 28.89 nm (undoped) to 24.26 nm (4 wt.% Zr). SEM imaging revealed smooth, densely packed surfaces with the appearance of nanonoodle-like structures in doped films. EDX and XPS confirmed the successful incorporation of Zr^4+^ ions. Optical studies showed increased transparency (up to 93%) and a blue shift in bandgap from 2.42 eV to 2.44 eV with doping, attributed to the Moss–Burstein effect. Electrical resistivity decreased significantly from 7.12 × 10^1^ Ω·cm (undoped) to 0.56 × 10^1^ Ω·cm (3 wt.% Zr), due to increased free carrier concentration from Zr^4+^ donating extra electrons. PL spectroscopy revealed enhanced emission intensity with doping, indicating increased sulfur vacancies and structural defects. Magnetic measurements showed a transition from paramagnetic behavior (undoped) to ferromagnetic ordering in doped films. Antibacterial activity was evaluated against Gram-negative *Klebsiella pneumoniae* using the agar well diffusion method. The zone of inhibition (ZOI) increased progressively with Zr doping, from 18 mm (undoped) to 31 mm (4 wt.% Zr). The enhanced activity was attributed to increased sulfur vacancies and free carrier concentration, leading to greater generation of reactive oxygen species (ROS) such as hydroxyl radicals and superoxide anions, which cause oxidative stress, membrane damage, and cell death. This study demonstrates that Zr doping significantly enhances the optoelectronic, magnetic, and antibacterial properties of CdS thin films, making them suitable for multifunctional device applications.

Neelgund et al. [124] developed antimicrobial nanohybrids (f-MWCNTs-CdS and f-MWCNTs-Ag_2_S) by covalently grafting fourth-generation amine-terminated PAMAM dendrimer onto multiwalled carbon nanotubes (MWCNTs) and subsequently depositing CdS and Ag_2_S quantum dots (QDs) in situ. Characterization (FTIR, XRD, Raman, TEM, EDS) confirmed successful PAMAM grafting and uniform QD deposition. Antibacterial activity was evaluated against Gram-negative *Escherichia coli* and *Pseudomonas aeruginosa*, and Gram-positive *Staphylococcus aureus* at 20 μg/mL. A stepwise enhancement in activity was observed: MWCNTs-COOH showed 14.7–26.8% reduction; f-MWCNTs showed 22.8–60% reduction; PAMAM dendrimer alone showed 17.7–49.7% reduction; CdS showed 39–61.2% reduction; Ag_2_S QDs showed 48–85.2% reduction. The f-MWCNTs-CdS nanohybrid achieved 46.7–87.2% reduction, while f-MWCNTs-Ag_2_S achieved 55.7–97.8% reduction, with the highest activity against *P. aeruginosa* (78.5–97.8%). Enhanced activity was attributed to synergistic effects: PAMAM’s amine groups facilitate bacterial membrane penetration, while CdS/Ag_2_S QDs generate reactive oxygen species and release ions, damaging DNA and proteins. All samples showed higher activity against Gram-negative bacteria due to their thinner peptidoglycan layer and negatively charged lipopolysaccharides facilitating dendrimer interaction. This study demonstrates that PAMAM-grafted MWCNTs decorated with QDs are highly effective, stable, water-dispersible antimicrobial agents.

Ali et al. [125] successfully synthesized a ZnO/CdS nanocomposite via a solution combustion method followed by chemical precipitation, aiming to enhance antibacterial activity. XRD analysis confirmed the presence of both hexagonal wurtzite ZnO and CdS phases, with an average crystallite size of 16 nm for the composite, smaller than pure ZnO (22 nm). TEM imaging revealed hexagonal-shaped nanoparticles, and EDX confirmed the elemental composition with no impurities. UV-Vis spectroscopy showed a blue shift in the absorption edge of the composite (328 nm) compared to pure ZnO (376 nm), corresponding to an increased bandgap from 3.36 eV to 3.74 eV, attributed to quantum confinement effects. Antibacterial activity was evaluated against Gram-positive *Staphylococcus aureus* using the standard zone of inhibition (ZOI) microbiology assay. The ZnO/CdS nanocomposite exhibited dose-dependent antibacterial activity, with inhibition zones increasing with concentration from 5 to 11 μg/mL. The composite showed more pronounced antibacterial effects than CdS synthesized by other methods, and was more effective against Gram-positive bacteria. The enhanced activity was attributed to the synergistic effect of ZnO and CdS, where the smaller particle size and increased bandgap facilitated greater interaction with bacterial cells and generation of reactive oxygen species. This study demonstrates that ZnO/CdS is a promising candidate for controlling skin infections caused by drug-resistant *S. aureus* strains.

Hu et al. [126] successfully synthesized mesoporous SiO_2_@CdS composite nanospheres with an average diameter of 300 nm via a facile process involving hydrolysis, calcination, and sulfidation. The composite exhibited a high BET surface area of 640 m^2^/g and an average pore size of 2.82 nm, enabling strong adsorption capacity. XRD confirmed hexagonal CdS nanocrystals (~6 nm) decorated on amorphous silica. TEM and HRTEM revealed uniform nanospheres with crystalline CdS on the surface. UV-Vis spectroscopy showed a characteristic CdS absorption peak at ~470 nm. The material demonstrated excellent photocatalytic activity, achieving complete degradation of Rhodamine B (4.8 mg/L, 50 mL) within 30 min under visible light using 40 mg of catalyst with higher CdS loading, attributed to enhanced light absorption and efficient electron–hole separation. Antibacterial activity was evaluated against *Escherichia coli* by incubating cells (1 × 10^5^/mL) with varying concentrations (25–500 μg/mL) of SiO_2_@CdS nanospheres for 24 h. The results showed concentration-dependent antibacterial efficacy, with approximately 70% bacterial kill at 50 μg/mL and nearly 95% kill at higher concentrations. The enhanced antibacterial activity was attributed to the generation of reactive oxygen species (ROS) by CdS under ambient light, causing oxidative stress and cell damage. The high surface area of the mesoporous silica also facilitated close contact with bacterial cells. This study demonstrates that mesoporous SiO_2_@CdS nanospheres are highly effective, reusable photocatalysts and promising antibacterial agents for environmental and biomedical applications. Hu et al. [38] synthesized mesoporous SiO_2_@CdS composite nanospheres (300 nm) with a high surface area (640 m^2^/g) and uniform pore size (2.82 nm). XRD confirmed hexagonal CdS nanocrystals (~6 nm) on amorphous silica. TEM showed uniform nanospheres with crystalline CdS on the surface. The composite exhibited excellent photocatalytic activity, achieving complete Rhodamine B degradation within 30 min under visible light. Antibacterial activity against *E. coli* (1 × 10^5^ cells/mL) was evaluated at 25–500 μg/mL for 24 h, showing concentration-dependent efficacy with ~70% kill at 50 μg/mL and ~95% at higher concentrations, attributed to ROS generation from CdS. This study highlights SiO_2_@CdS as a highly effective photocatalyst and antibacterial agent.

Rani and Chauhan [127] successfully synthesized novel CdS/ZnS heterostructure nanocomposites via a hydrothermal method at varying reaction temperatures (140, 160, and 180 °C) and evaluated their antibacterial activity for the first time. XRD confirmed the coexistence of hexagonal CdS and ZnS phases, with crystallite sizes decreasing from 14.75 to 9.16 nm as temperature increased, indicating improved crystallinity. SEM revealed flower-like morphology, which enhances bacterial interaction. TEM showed particle size decreasing from 39 to 32 nm with increasing temperature, with corresponding increases in interplanar spacing (0.12 to 0.17 nm). UV-Vis spectroscopy showed two distinct absorption edges, with bandgaps narrowing significantly for the 180 °C sample (2.67 and 1.52 eV) compared to 140 °C (3.37 and 2.75 eV), attributed to enhanced charge transfer between CdS and ZnS. PL spectroscopy revealed decreased emission intensity at higher temperatures, indicating reduced electron–hole recombination. BET analysis showed that the 180 °C sample had the highest surface area (185.45 m^2^/g) and smallest pore diameter (2.25 nm), providing more active sites. Antibacterial activity against *E. coli* (Gram-negative) and *S. aureus* (Gram-positive) was evaluated via agar well diffusion at 50–250 μg/mL. The CdS/ZnS-180 nanocomposite exhibited the highest activity, with inhibition zones of 25 mm (*E. coli*) and 21 mm (*S. aureus*) at 250 μg/mL, significantly outperforming bare CdS (16 mm, 15 mm) and ZnS (14 mm, 13 mm). The enhanced activity was attributed to efficient type I band alignment promoting charge separation, increased ROS generation, high surface area, and flower-like morphology facilitating bacterial membrane penetration. This study demonstrates CdS/ZnS heterostructures as promising antibacterial agents.

## 8. Antibacterial Performance of CdS/X/X Composites

Building upon the success of binary heterojunctions, ternary CdS-based composites represent the next frontier in photocatalytic antibacterial design. By incorporating two complementary functional components, for example, a second semiconductor for cascade charge transfer, a noble metal cocatalyst for surface plasmon resonance or electron trapping, and/or a magnetic or mesoporous support for recoverability and enhanced adsorption, ternary systems achieve synergistic effects unattainable in binary or pristine counterparts. Among the most promising architectures are Z-scheme and S-scheme heterojunctions, which preserve the strong redox potentials of both components while spatially separating photogenerated carriers, leading to exceptionally high ROS generation and rapid bacterial inactivation (often >90% within 10–60 min). This section examines representative ternary nanocomposites, including Bi_2_S_3_/CdS, CdS/ZIF-67, Fe_3_O_4_/CdS, and polymer-supported CdS/ZnO/Ag_3_PO_4_ systems. Emphasis is placed on how interfacial engineering, band alignment, and the synergistic interplay among three components enhance antibacterial efficacy beyond binary counterparts. Table 4 provides a consolidated overview of these advanced ternary systems, including their activity against pathogens and proposed mechanisms of action.

Mushtaq et al. synthesized a ternary S-g-C_3_N_4_/Cr-doped CdS (Cr-CdS@SCN) hybrid nanocomposite via a chemical co-precipitation method, evaluating its multifunctional potential in photocatalysis and biomedicine [128] (Table 4). For biological applications, the study assessed antibacterial, antioxidant, and enzyme inhibition activities. The antibacterial efficacy was tested against Gram-negative *E. coli* and Gram-positive *S. aureus* using the agar well diffusion method at concentrations ranging from 0.5 to 2.0 mg/mL. The 5% Cr-CdS@70% SCN nanocomposite exhibited the strongest antibacterial effect, with inhibition zones of 23 mm against *E. coli* and 22 mm against *S. aureus* after 24 h of incubation, outperforming pure and doped CdS (Table 4). This enhanced activity was attributed to ROS generation and bacterial membrane damage.

Mughal et al. synthesized [129] TiO_2_/CdS/PbS and TiO_2_/PbS/CdS hierarchical nanocomposites via a pseudo-successive ionic layer adsorption and reaction (p-SILAR) technique, investigating their antibacterial efficacy alongside photocatalytic and electrochemical properties. The antibacterial activity was assessed against Gram-negative *Escherichia coli* (*E. coli*) using the agar well diffusion method. A bacterial inoculum of 100 μL/100 mL was incorporated into nutrient agar, and after solidification, wells were filled with 50–70 μL of the nanocomposite samples. The plates were incubated at 37 °C for 24 h, and the zones of inhibition were measured. TiO_2_/PbS/CdS exhibited the strongest antibacterial effect with an inhibition zone of ~14 mm, significantly higher than pristine TiO_2_ (~8 mm) and TiO_2_/CdS (~11 mm). TiO_2_/CdS/PbS showed a reduced inhibition zone of ~9 mm. The enhanced antibacterial activity of TiO_2_/PbS/CdS was attributed to its favorable band-aligned structure, which facilitated efficient electron transfer and reactive oxygen species (ROS) generation under visible light, leading to bacterial cell membrane damage and death. The study underscores the role of quantum dot sensitization in amplifying the photobiological activity of TiO_2_-based nanocomposites.

Shi et al. synthesized [130] a series of hierarchical Z-scheme Bi_2_S_3_/CdS heterojunctions via a one-pot solvothermal method, using different solvents (DMF, EG, DI) to achieve solvent-dependent morphological control. Among these, the Bi_2_S_3_/CdS composite synthesized in DMF (BC-DMF) exhibited a well-ordered hierarchical nanoflower structure with CdS uniformly distributed on Bi_2_S_3_ nanowire surfaces, providing enhanced photocatalytic active centers and improved light harvesting. The photocatalytic antibacterial activity was evaluated against Gram-negative *Escherichia coli* (*E. coli*) and Gram-positive *Staphylococcus aureus* (*S. aureus*) using the plate counting method. For the assay, 50 mg of BC-DMF photocatalyst was dispersed in 100 mL of bacterial suspension and irradiated under visible light (>420 nm) using a 300 W Xenon lamp. After 10 min of irradiation, BC-DMF achieved an antibacterial efficiency of 93.03% against *E. coli*, significantly outperforming pure Bi_2_S_3_ (55.41%), CdS (40.11%), and P25 (54.70%). Under extended irradiation (100 min), BC-DMF showed 93.85% antibacterial efficiency against *S. aureus*. The enhanced activity was attributed to the Z-scheme heterojunction mechanism, which facilitated efficient photo-induced electron–hole separation and transfer, preserving the strong reduction ability of CdS and oxidation ability of Bi_2_S_3_, thereby promoting the generation of superoxide radicals (•O_2_^−^) as the primary reactive species for bacterial inactivation [130,131]. The study highlights the potential of morphology-controlled Z-scheme heterojunctions for efficient photocatalytic disinfection of pathogenic bacteria in wastewater.

Midya et al. developed [132] a novel nanocomposite, cl-Ch-pMAc@ZnO/CdSQs_2_, by embedding ZnO and CdS quantum dots onto anionically functionalized crosslinked chitosan (cl-Ch-pMAc) under microwave irradiation, and evaluated its antibacterial activity against Gram-positive *Bacillus subtilis* and Gram-negative *Escherichia coli*. The antibacterial efficacy was assessed using the disk diffusion method on Mueller Hinton agar plates. Bacterial suspensions at a concentration of 10^7^ cells/mL were uniformly spread, and filter paper disks loaded with varying concentrations of the nanocomposite (6–18 mg/dish) were placed on the plates. Taxim (4 mg/dish) served as a positive control, while a disk without nanocomposite was used as a negative control. The plates were incubated at 37 °C for 24 h, and the ZOI were measured. The nanocomposite exhibited significant antibacterial activity, with inhibition zones ranging from 5 to 40 mm depending on the concentration and bacterial strain. For *B. subtilis*, a clear inhibition zone was observed at 18 mg/dish, while for *E. coli*, activity was evident at 12 mg/dish. No inhibitory effect was observed below 6 mg/dish. The antibacterial mechanism was attributed to the release of Zn^2+^ ions and the generation of ROS such as superoxide anions (O^2−^) from ZnO under light, which disrupt bacterial cell walls and cause cell death. The study highlights the potential of this chitosan-based nanocomposite as an effective, reusable, and environmentally benign antibacterial agent.

Guo et al. synthesized [133] BiVO_4_/CdS heterojunctions with different morphologies via a deposition–precipitation method, using citric acid and CTAB as dispersants to obtain spherical BiVO_4_ (BS) and sheet-like BiVO_4_ nanoarrays (BA), respectively. CdS was subsequently deposited on the BiVO_4_ surfaces to form BSC-x and BAC-x composites. The photocatalytic antibacterial activity was evaluated against Gram-negative *Escherichia coli* (*E. coli*) under visible-light irradiation (>420 nm) using a 300 W Xe lamp. Bacterial suspensions at an initial concentration of 1.0 × 10^6^ CFU/mL were mixed with the photocatalysts, and aliquots were taken at 30 min intervals over 120 min to assess inactivation. Among the composites, BAC-60 (with 60% CdS mass ratio on BA) exhibited the highest bactericidal performance, achieving approximately 98% inactivation of *E. coli* within 60 min of irradiation, outperforming pure CdS and BiVO_4_. The enhanced activity was attributed to the Z-scheme charge transfer mechanism at the BiVO_4_/CdS interface, which promoted efficient separation of photogenerated electron–hole pairs and preserved the strong reduction ability of CdS and oxidation ability of BiVO_4_. MALDI-TOF-MS analysis revealed that the degradation of low-molecular-weight proteins (2–6 kDa) was responsible for bacterial inactivation. The study also confirmed that the (020) exposed facet of BiVO_4_ nanoarrays contributed to higher photocatalytic activity compared to spherical BiVO_4_. This work highlights the importance of morphology control and heterojunction design in developing efficient visible-light-driven photocatalysts for bacterial disinfection.

Min et al. synthesized [134] NaNbO_3_/CdS nanorod composites (NNO-C) via a two-step hydrothermal method and evaluated their pyrocatalytic antibacterial activity against *Salmonella* typhimurium under room-temperature cold–hot cycles. The composites were prepared by loading CdS onto NaNbO_3_ nanorods, with NNO-C2 (40 wt% CdS) exhibiting optimal performance. For antibacterial testing, 10 mg of NNO-C2 and 10 mg of CdS were separately dispersed in 49.5 mL of 85% NaCl solution, followed by the addition of 1 mL of a 10^3^-fold diluted *Salmonella* suspension. The mixture was stirred in the dark for 15 min, then subjected to cold–hot cycles between 17 °C and 37 °C. After every nine cycles, 300 µL of the solution was collected, and 100 µL was spread on solid media and incubated at 37 °C for 12 h. NNO-C2 achieved approximately 90% antibacterial rate after 45 cold–hot cycles, significantly outperforming pure CdS (~60%) and pure NaNbO_3_. The enhanced activity was attributed to the S-scheme heterojunction formed between NaNbO_3_ and CdS, which facilitated efficient separation of pyroelectric-induced electron–hole pairs under temperature fluctuation, generating reactive oxygen species (ROS) such as superoxide radicals (•O_2_^−^) and hydroxyl radicals (•OH). These ROS disrupted bacterial cell membranes, inhibited protein synthesis, and caused DNA damage, leading to cell death. The study demonstrates the potential of pyrocatalytic nanocomposites for disinfection in environments with natural temperature variations, offering a sunlight-independent antibacterial strategy.

Liang et al. developed [135] an S-scheme CdS@ZM heterojunction by in situ coating of CdS quantum dots (QDs) onto ZIF-67 dodecahedrons (ZM) synthesized in methanol. The exposed Co^2+^ centers in ZM acted as anchoring sites for S^2−^ precursors, enabling the formation of highly dispersed, tightly integrated CdS (2–4 nm) with intimate interfacial contact. The photocatalytic antibacterial activity was evaluated against *Staphylococcus aureus* under visible light (λ ≥ 420 nm). In the assay, 10 mg of photocatalyst was dispersed in 40 mL saline, and 100 µL of bacterial suspension (2.0 × 10^9^ CFU/mL) was added. After irradiation, aliquots were diluted, spread on agar plates, and incubated at 37 °C for 18 h. The 40% CdS@ZM composite achieved 97.6% sterilization within 15 min, significantly outperforming pure ZM (51.0%) and CdS (21.9%). The enhanced activity was attributed to the S-scheme mechanism, which facilitated efficient charge separation and generated reactive oxygen species (•O_2_^−^, •OH, h^+^), causing severe bacterial cell wall damage. This study highlights the potential of MOF-based S-scheme heterojunctions for rapid photocatalytic disinfection.

Iftikhar et al. synthesized [136] Fe-doped CdS (Fe@CdS) and a nanocomposite with sulfonated graphitic carbon nitride (SCN) via a co-precipitation method, and evaluated their antibacterial activity against *Staphylococcus aureus* (Gram-positive) and *Escherichia coli* (Gram-negative) using the agar well diffusion method. For the assay, 100 µL of bacterial inoculum was spread on nutrient agar plates, and wells were filled with 100 µL of sample solutions at varying concentrations. The plates were incubated at 37 °C for 24–48 h, and ZOI values were measured. The 9% Fe@CdS with 50% SCN nanocomposite exhibited the highest antibacterial activity, with inhibition zones ranging from 5 to 20 mm, significantly outperforming pure CdS and SCN. The enhanced activity was attributed to the increased surface area and reduced electron–hole pair recombination due to SCN incorporation, which promoted the generation of ROS, leading to bacterial cell damage. The study demonstrates the potential of Fe@CdS/SCN heterojunctions as effective antibacterial agents for biomedical and environmental applications.

Kebede et al. synthesized [137] a novel polyaniline-supported ternary n-n heterojunction, PANI/CdS-ZnO-Ag_3_PO_4_ (PANI-CdS-ZnO-AP), via impregnation and evaluated its photocatalytic antibacterial activity against four bacterial strains: *Escherichia coli*, Shigella (Gram-negative), *Streptococcus*, and *Staphylococcus aureus* (Gram-positive). The antibacterial assay was performed using the paper disk diffusion technique under visible-light irradiation (85 W fluorescent lamp, 480 nm). Sterilized filter paper disks (6 mm diameter) were impregnated with 0.30 mg/L of the photocatalyst solution and placed on nutrient agar plates seeded with the respective bacteria. The plates were incubated at 37 °C for 24 h, and ZOI were measured. While ZOI values were not explicitly reported in the main text, the study demonstrated that PANI-CdS-ZnO-AP exhibited significantly higher antibacterial efficiency compared to binary (AP-ZnO, CdS-ZnO) and single systems (ZnO, CdS, Ag_3_PO_4_). The enhanced activity was attributed to the tandem n-n heterojunction structure, which facilitated efficient separation of photogenerated electron–hole pairs, generating ROS such as •O_2_^−^ and •OH that cause bacterial cell damage. Statistical analysis (ANOVA, Tukey’s test) confirmed the superior performance of the supported ternary composite, highlighting its potential for environmental disinfection applications.

Ikram et al. synthesized [138] chitosan (CS) and carbon nitride (C_3_N_4_) co-doped CdS (C_3_N_4_/CS-CdS) composites via a co-precipitation method and evaluated their antibacterial activity against *Staphylococcus aureus* (Gram-positive) and *Escherichia coli* (Gram-negative) using the well diffusion method. Bacterial cultures at 1.5 × 10^8^ CFU/mL were swabbed on agar plates, and 6 mm wells were filled with 50 μL of sample concentrations (500 and 1000 μg/50 μL). Ciprofloxacin (5 μg/50 μL) and deionized water served as positive and negative controls. Plates were incubated at 37 °C for 48 h, and inhibition zones were measured. The 4% C_3_N_4_/CS-CdS nanocomposite exhibited the highest antibacterial activity, with inhibition zones of 4.05 mm against *S. aureus* and 2.45 mm against *E. coli* at 1000 μg/50 μL, outperforming pure CdS, CS-CdS, and 2% C_3_N_4_ samples. The enhanced activity was attributed to the synergistic effect of CS and C_3_N_4_, which improved reactive oxygen species (ROS) generation and facilitated bacterial cell membrane disruption through electrostatic interaction with negatively charged bacterial surfaces. The study demonstrates the potential of biopolymer and carbon nitride co-doped CdS composites as effective antibacterial agents for environmental and biomedical applications.

Wang et al. synthesized [139] CdSe/CdS/N-acetyl-L-cysteine (NAC)/quercetin nanocomposites to enhance the water solubility and bioactivity of quercetin while imparting fluorescence properties for potential biomedical applications. The nanocomposites were characterized using HRTEM, SEM, XRD, FTIR, TGA, UV-vis, and photoluminescence spectroscopy, confirming successful coating of quercetin on the core–shell CdSe/CdS/NAC quantum dots with homogeneous particle size and good monodispersity. The antibacterial activity was evaluated against Gram-positive *Staphylococcus aureus*, *Enterococcus faecalis* and Gram-negative *Escherichia coli*, *Pseudomonas aeruginosa* using optical density and streak plate methods. Bacterial strains (200 µL) were incubated in 50 mL LB liquid medium with the nanocomposites at 37 °C for 12 h. The minimum inhibitory concentration (MIC) values ranged from 1.57 to 25 mg/mL, demonstrating significant antibacterial efficacy. The mechanism was attributed to the combined effects of quercetin’s inherent antimicrobial properties and the quantum dots’ ability to generate reactive oxygen species (ROS), leading to bacterial cell membrane damage and cell death. The study highlights the potential of quercetin-functionalized quantum dots as multifunctional agents for drug delivery and targeted cancer therapy, combining strong fluorescence with antibacterial activity.

Shariati et al. synthesized [140] reverse type I core/shell ZnS/CdS quantum dots decorated with dual cocatalysts (Ag nanoparticles and PdS) to enhance photocatalytic antibacterial activity. The Ag-PdS/ZnS/CdS nanohybrids were characterized using XRD, STEM-EDS, steady-state and time-resolved photoluminescence spectroscopy, confirming efficient spatial separation and transfer of photogenerated charge carriers. Antibacterial activity was evaluated against *Escherichia coli* (Gram-negative) and Staphylococcus saprophyticus (Gram-positive) using broth tube dilution and disk diffusion methods. Bacterial suspensions (1 mL, 0.5 McFarland standard) were mixed with varying photocatalyst concentrations (2.0–11.0 mg/mL), irradiated under solar light for 30 min, incubated at 37 °C for 3 h, and then plated on agar for 18 h to determine MIC and MBC values. The Ag-PdS/ZnS/CdS composite exhibited the lowest MIC (3.0 mg/mL for *E. coli*, 2.0 mg/mL for *S. saprophyticus*) and MBC (5.0 and 4.0 mg/mL, respectively), outperforming ZnS/CdS, CdS, and ZnS alone. The enhanced activity was attributed to the dual cocatalysts facilitating efficient electron–hole separation, generating reactive oxygen species (•O_2_^−^ and •OH) that caused lipid peroxidation, protein oxidation, and DNA damage. The study demonstrates the potential of dual cocatalyst-loaded reverse type I core/shell QDs for effective photocatalytic disinfection.

Kiani et al. synthesized [141] a novel Pt-Ag_3_PO_4_/CdS/chitosan nanocomposite via a multi-step method and evaluated its antibacterial activity against six bacterial strains, Gram-positive *Staphylococcus aureus*, *Bacillus cereus* and Gram-negative *Escherichia coli*, *Pseudomonas aeruginosa*, *Salmonella typhimurium*, and *Klebsiella pneumoniae* using the disk diffusion method under visible-light and dark conditions. Bacterial cultures were spread on Muller Hinton agar, and disks soaked with 0–20 μg/mL of the nanocomposite were placed on the plates, incubated at 37 °C for 24 h, and ZOI measured. At 20 μg/mL under visible light, Pt-Ag_3_PO_4_/CdS/chitosan exhibited superior antibacterial activity with ZOI values ranging from approximately 0 to 44 mm, significantly outperforming CdS and CdS/Ag_3_PO_4_. The enhanced activity was attributed to the synergistic effects of Pt nanoparticles acting as electron sinks, delaying electron–hole recombination and promoting ROS generation, particularly •OH radicals, which caused bacterial cell membrane damage. The chitosan matrix improved stability and adsorption. The nanocomposite also showed good cytocompatibility on HEK-293, HeLa, HepG2, and PC12 cell lines. This study demonstrates the potential of Pt-decorated Ag_3_PO_4_/CdS/chitosan as an efficient visible-light-driven antibacterial agent for environmental and biomedical applications.

Malekkiani et al. synthesized [142] a ternary Ag-CdS/MWCNTs heterojunction nanocomposite by anchoring CdS and silver nanoparticles (Ag NPs) onto multiwalled carbon nanotubes (MWCNTs) and evaluated its antibacterial activity against Gram-positive *Staphylococcus aureus*, *Bacillus subtilis* and Gram-negative *Escherichia coli* using the disk diffusion method. Bacterial cultures were spread on nutrient agar plates, and sterilized filter paper disks impregnated with 15 µL of the nanocomposite solution were placed on the inoculated surfaces. The plates were incubated overnight at 37 °C, and zones of inhibition (ZOIs) were measured. The Ag-CdS/MWCNTs nanocomposite exhibited clear inhibition zones with diameters of 10.7 mm against *S. aureus*, 9.88 mm against *B. subtilis*, and 3.34 mm against *E. coli*, demonstrating greater efficacy against Gram-positive bacteria. The enhanced antibacterial activity was attributed to the generation of reactive oxygen species (ROS) under light irradiation, facilitated by the synergistic effect of CdS and Ag NPs on the MWCNT support, which promoted efficient electron–hole separation and charge transfer. The MWCNTs served as electron acceptors, reducing recombination and prolonging the lifetime of charge carriers. The study highlights the potential of Ag-CdS/MWCNTs as an effective photocatalyst for water remediation and antibacterial applications.

**Figure 8:**
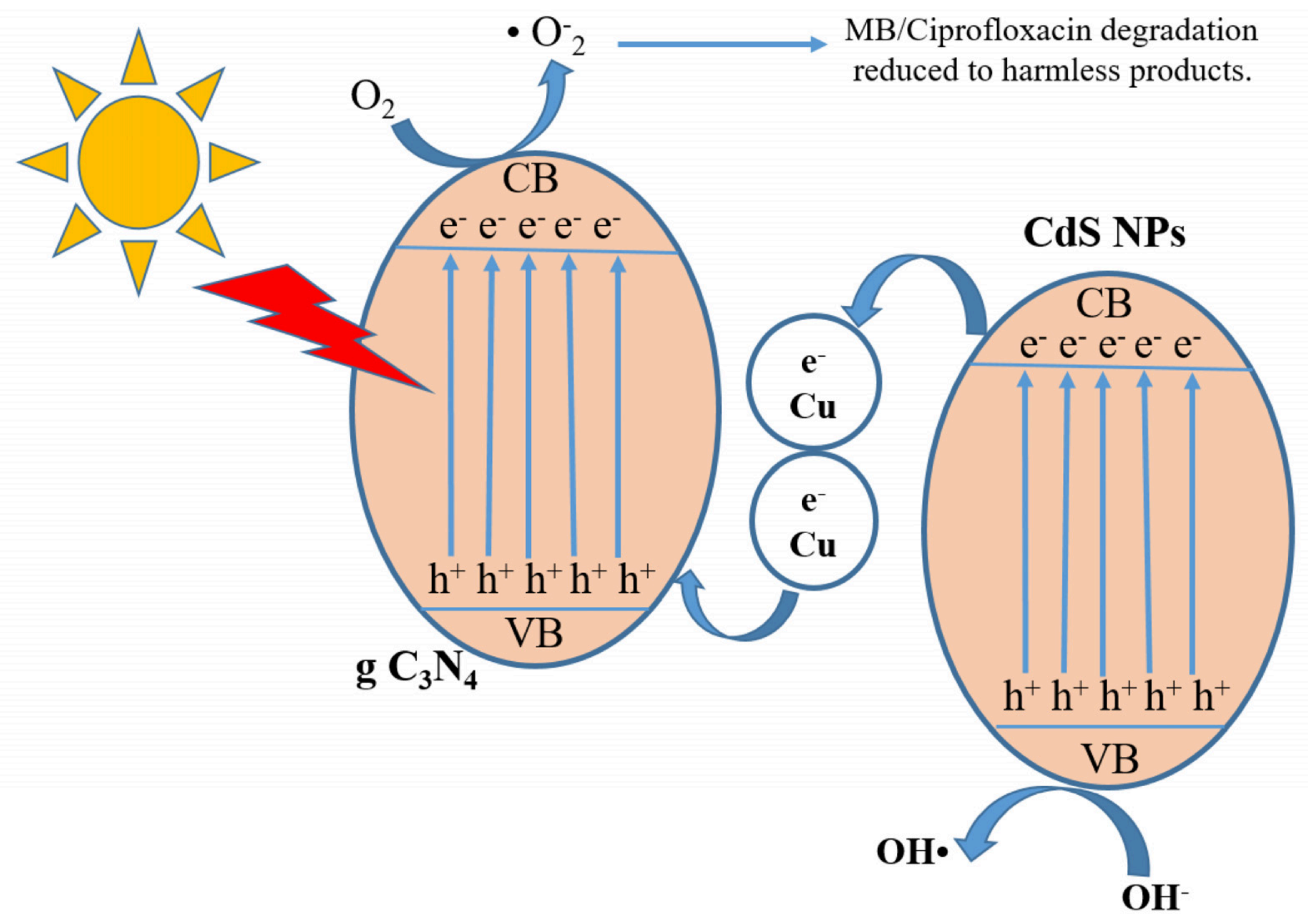
Mechanism of photocatalytic activity [143]. Reprinted with permission from [143] (CC BY license).

Afzal et al. synthesized [143] a ternary g-C_3_N_4_/Cu@CdS nanocomposite via co-precipitation and evaluated its antibacterial activity against *Staphylococcus aureus* (Gram-positive) and *Escherichia coli* (Gram-negative) using the agar well diffusion method (Figure 8). Stock suspensions of CdS, Cu@CdS, g-C_3_N_4_, and g-C_3_N_4_/Cu@CdS were prepared at concentrations of 50–200 mg/mL. Muller Hinton agar plates were inoculated with bacterial strains, and wells were filled with 20 µL of each sample. Ciprofloxacin (1 mg/mL) served as a positive control. Plates were incubated at 37 °C for 24 h, and zones of inhibition (ZOI) were measured. The 5% Cu@CdS exhibited the highest activity against *S. aureus*, while 7% Cu@CdS was most effective against *E. coli*. The g-C_3_N_4_/Cu@CdS nanocomposite showed moderate antibacterial activity. The enhanced effect was attributed to Cu doping, which reduced the bandgap and improved charge separation, promoting ROS generation under sunlight. The composite also demonstrated excellent photocatalytic degradation of methylene blue (95%) and ciprofloxacin (76%). This study highlights the potential of Cu-doped CdS-based ternary nanocomposites for dual environmental and antibacterial applications.

Sabah et al. synthesized [144] highly luminescent CdSe/CdS/ZnS core–multi-shell quantum dots via a phosphine-free precursor injection method in paraffin liquid and oleic acid, and further encapsulated them with polymers (PEG, PVA, PVP, PAA) to enhance stability. The QDs exhibited cubic zinc blende structure with crystallite sizes ranging from 22 to 44 nm and strong UV absorption (302–380 nm). After 48 h of sunlight exposure, absorption and emission red-shifted to 389–464 nm and 492–509 nm, respectively, indicating enhanced visible light activity. Antibacterial activity was evaluated against Gram-negative *Pseudomonas aeruginosa* and *Klebsiella pneumoniae* using the agar well diffusion method. Bacterial cultures were incubated at 37 °C for 24 h, and zones of inhibition (ZOIs) were measured. CdSe/CdS/ZnS QDs showed ZOI of 18–20 mm against *P. aeruginosa* with tetracycline, and 19 mm against *K. pneumoniae* with cefotaxime. Polymer-capped QDs also exhibited significant activity against *P. aeruginosa*, with ZOI ranging from 2 to 20 mm. The antibacterial mechanism was attributed to reactive oxygen species (ROS) generation causing bacterial membrane damage. The study demonstrates the potential of core–multi-shell QDs for optoelectronic and biomedical applications.

Gupta et al. synthesized [145] ZnO-CdS-Ag ternary nanocomposites with two different ZnO morphologies, nanorods (NRs) and combustion-synthesized nanoparticles (CSs), and evaluated their photocatalytic antibacterial activity against *Escherichia coli* under UV and visible light. The nanocomposites were prepared by embedding CdS on ZnO via precipitation, followed by Ag impregnation using hydrazine reduction. Antibacterial experiments were conducted with an initial bacterial concentration of ~10^7^ CFU/mL and catalyst loadings of 0.1–0.5 g/L. After 1 h of dark adsorption, the suspensions were irradiated for 1 h, and samples were serially diluted and spread on agar plates for colony counting. The optimum catalyst loading was 0.25 g/L. The ZnO(NR)-CdS-Ag composite exhibited the highest photocatalytic inactivation, with rate constants of 11 ± 0.3 h^−1^ under UV and 12 ± 0.6 h^−1^ under visible light, outperforming ZnO(CS)-CdS-Ag and physical mixtures. The enhanced activity was attributed to the high aspect ratio of nanorods facilitating unidirectional charge transfer, CdS acting as a visible-light photosensitizer, and Ag serving as an electron trap to suppress recombination. This study highlights the critical role of ZnO morphology in designing efficient ternary photocatalysts for bacterial disinfection.

Kang et al. developed [146] a ternary hybrid CdS/Pt-TiO_2_ nanotube (NT) photoelectrode by depositing Pt nanoparticles via a dipping and deposition technique and CdS via successive ionic layer adsorption and reaction (SILAR) onto anodized TiO_2_ nanotubes. The hybrid structure exhibited enhanced visible-light absorption, reduced electron–hole recombination, and improved photoelectrochemical activity due to the synergistic effects of CdS sensitization and Pt-mediated charge separation. The antibacterial activity was evaluated against *Escherichia coli* (~5 × 10^8^ CFU/mL) under simulated solar light irradiation with an applied bias of 0.6 V. Bacterial survival was assessed by plate counting after 24 h incubation. The CdS/Pt-TiO_2_ NT electrode achieved 99.2% inactivation within 60 min, significantly outperforming Pt-TiO_2_ NTs (85.1%) and pure TiO_2_ NTs (51.4%). The enhanced performance was attributed to efficient photogenerated electron transfer from CdS to TiO_2_ and then to Pt, which suppressed recombination and promoted the generation of ROS such as •OH and H_2_O_2_, leading to bacterial membrane damage. FTIR analysis confirmed degradation of bacterial proteins. This study demonstrates the potential of ternary hybrid nanotube arrays for photoelectrocatalytic disinfection.

Ranjith et al. synthesized [147] Ni/Ce co-doped CdS nanoparticles (NPs) via a hydrothermal method and evaluated their antibacterial activity against Gram-negative *Escherichia coli* and Gram-positive *Staphylococcus aureus* using the agar well diffusion method. Bacterial suspensions (0.1 mL of cultivated bacteria) were spread on agar plates, and wells were filled with 25–75 µL/mL of pure or Ni/Ce co-doped CdS colloidal solutions dispersed in DMSO. Plates were incubated at 37 °C for 24 h, and zones of inhibition (ZOIs) were measured. The Ni/Ce co-doped CdS NPs exhibited significantly enhanced antibacterial activity compared to pure CdS, with ZOI ranging from 4.0 to 21.0 mm depending on concentration and bacterial strain. At 75 µL/mL, the co-doped NPs showed ZOIs of 21.0 mm against *E. coli* and 10.5 mm against *S. aureus*, outperforming pure CdS (8.2 mm and 11.5 mm, respectively). The enhanced activity was attributed to the synergistic effect of Ni and Ce co-doping, which narrowed the bandgap, reduced electron–hole recombination, and promoted the generation of ROS under visible light, leading to bacterial cell membrane damage. The study demonstrates the potential of transition metal and rare-earth co-doping in CdS for improved photocatalytic and antibacterial applications.

## 9. Atomistic Modeling and Simulation Details

**Figure 9:**
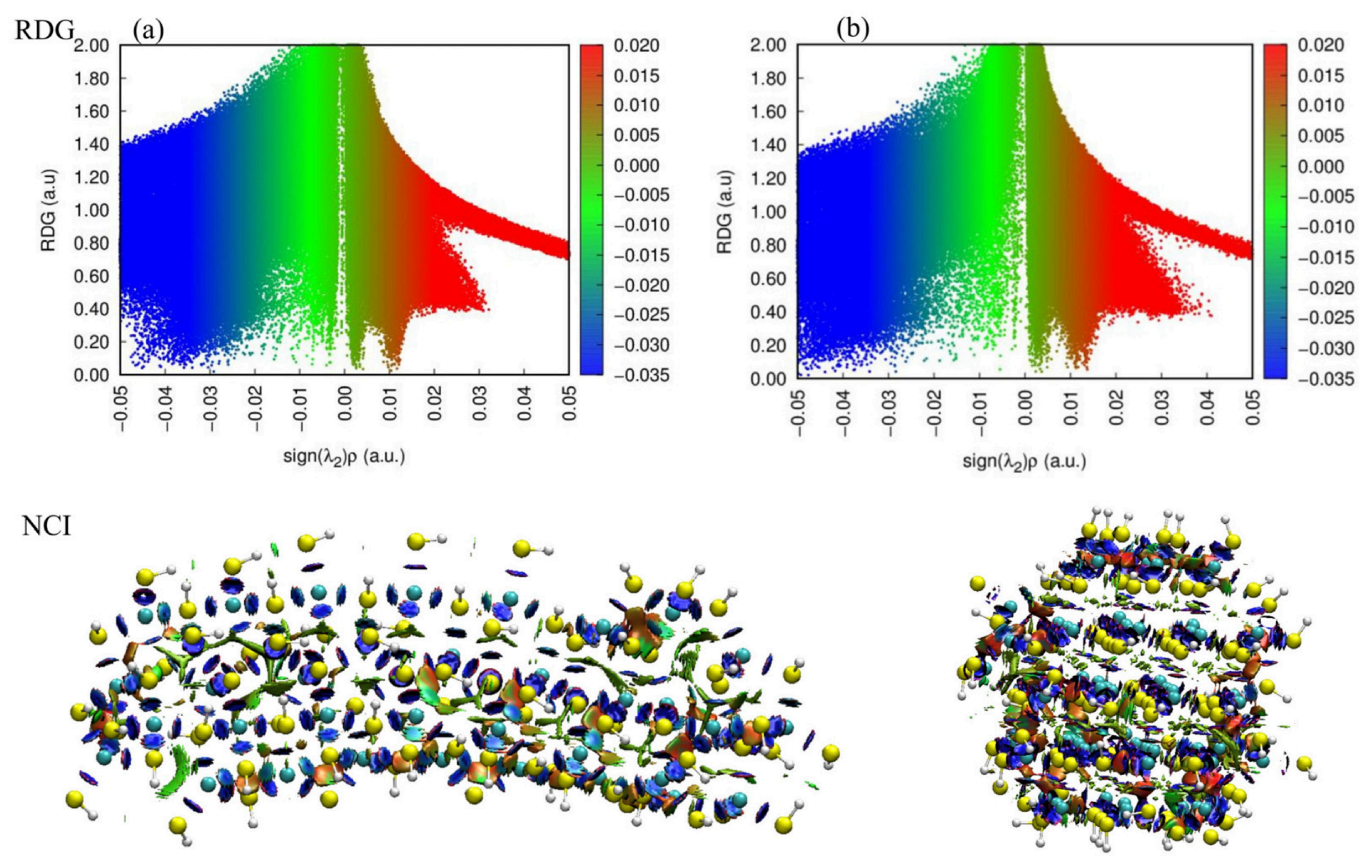
RDG and NCI plots of (**a**) rod-shaped CdS and (**b**) cluster-shaped CdS optimized in an implicit water environment. Atom colors are cyan for Cd, yellow for S, and white for H. The RDG/NCI isosurfaces are colored blue, green, and red to indicate strong attractive, van der Waals, and strong repulsive interactions, respectively. Reprinted with permission from [17] (CC BY license).

Computational approaches have emerged as powerful tools for elucidating the fundamental mechanisms governing the enhanced photocatalytic and antibacterial performance of CdS (Figure 9) [148,149,150].

Ahmed et al. [111] performed molecular docking simulations to investigate the interaction mechanisms of Ni-Cu co-incorporated CdS (Cd_60_Ni_20_Cu_20_S) with key microbial enzyme targets. The docking results revealed binding energies of −2.8 kcal mol^−1^ for both FabH and β-lactamase, with the Ni-Cu-incorporated CdS forming specific interactions with ASN-247 (bond distance 3.6 Å) in FabH and ARG-260 (bond distance 3.7 Å) in β-lactamase. These enzymes are critically involved in bacterial fatty acid biosynthesis (FabH) and peptidoglycan biosynthesis (β-lactamase); their inhibition leads to cell wall rupture and bacterial death. For antifungal activity, docking against glucoamylase (PDB ID: 1KUL) yielded a binding energy of −2.1 kcal mol^−1^ with interaction at THR-599 (3.7 Å), while urate oxidase (PDB ID: 1R51) showed a binding energy of −2.3 kcal mol^−1^ with interaction at GLU-221 (3.4 Å). These computational predictions correlated well with experimental antimicrobial results, where Cd_60_Ni_20_Cu_20_S nanoparticles exhibited inhibition zones of 26 mm against *E. coli*, 25 mm against *Lactobacillus*, 24 mm against *Aspergillus niger*, and 28 mm against *Aspergillus flavus*. The molecular docking approach thus provided mechanistic validation for the experimentally observed antimicrobial efficacy, demonstrating that Ni-Cu co-doped CdS acts as a potential enzyme inhibitor through specific amino acid residue interactions.

In a complementary computational study, Shi et al. [130] employed DFT calculations using the CASTEP code to investigate the interfacial charge transfer mechanisms in hierarchical Z-scheme Bi_2_S_3_/CdS heterojunctions. The work functions (Φ) of Bi_2_S_3_, CdS, and the Bi_2_S_3_/CdS heterojunction were calculated using the equation Φ = *E_vac_* − *E_F_*, where *E_vac_* represents the electrostatic potential of the vacuum level near the surface and *E_F_* is the Fermi energy level. The calculations revealed work function values of 4.79 eV for Bi_2_S_3_ (101), 5.67 eV for CdS (101), and 5.42 eV for the Bi_2_S_3_/CdS heterojunction. The corresponding Fermi levels were determined to be −6.94 eV and −5.02 eV for Bi_2_S_3_ and CdS, respectively. This significant difference in Fermi levels (approximately 1.92 eV) drives spontaneous charge transfer from CdS to Bi_2_S_3_ upon contact until Fermi level equilibrium is achieved at −6.55 eV, resulting in the generation of an internal electric field at the heterointerface. Critically, this internal electric field facilitates the recombination of photogenerated electrons from the conduction band of Bi_2_S_3_ with holes from the valence band of CdS, while preserving the strongly reducing electrons in the CdS conduction band and the strongly oxidizing holes in the Bi_2_S_3_ valence band, a hallmark of Z-scheme charge transfer. The calculated work functions were validated by ultraviolet photoelectron spectroscopy (UPS) measurements, which yielded values of 4.37 eV, 4.97 eV, and 4.87 eV for Bi_2_S_3_, CdS, and the heterojunction, respectively, showing excellent agreement with the DFT predictions.

Together, these computational studies demonstrate that atomistic modeling—ranging from DFT calculations of electronic structure and charge transfer to molecular docking of enzyme inhibitor interactions—provides indispensable mechanistic insights that guide the rational design of CdS-based photocatalysts for antimicrobial applications.

## 10. Comprehensive Analysis and Critique of CdS for Antibacterial Applications and Future Perspective

The collective evidence from over a decade of research establishes CdS as a potent, visible-light-driven antibacterial agent. Pristine CdS demonstrates inhibition zones ranging from 6 to 35 mm depending on morphology, synthesis method, and target microorganism, with quantum dots (2–10 nm) exhibiting enhanced membrane penetration and ROS generation.

A systematic analysis of the data compiled in Table 5 reveals several critical insights that transcend individual study results. The most striking observation is the clear hierarchy in antibacterial performance that emerges from increasingly sophisticated material engineering: ternary S-scheme heterojunctions > ternary Z-scheme systems > binary composites > doped CdS > pristine CdS > encapsulated systems. This hierarchy reflects the fundamental importance of charge separation efficiency in determining photocatalytic antibacterial activity, as systems that more effectively suppress electron–hole recombination consistently achieve superior bacterial inactivation.

Analysis across several studies reveals that hexagonal wurtzite CdS consistently outperforms cubic sphalerite counterparts, with ZOI values higher for wurtzite-phase materials. This enhancement is attributed to the polar surfaces and internal electric fields inherent to the wurtzite structure, which facilitate spatial charge separation. However, the metastable cubic phase, when stabilized in quantum dots (<10 nm), can achieve comparable activity through quantum confinement effects that increase the bandgap and enhance redox potential. Particle size demonstrates an inverse relationship with antibacterial activity. This size-dependent activity is attributed to the exponentially increasing surface-area-to-volume ratio, enhanced membrane penetration for sub-20 nm particles, and quantum confinement effects that optimize band edge positions.

A hierarchical 3D architecture consistently outperforms lower-dimensional counterparts, with rose-like structures achieving higher ZOI values compared to 18–22 mm for simple spherical nanoparticles. The superior performance of 3D structures arises from multiple synergistic effects: high specific surface areas providing abundant active sites, enhanced light harvesting through multiple scattering events, mesoporous structures facilitating bacterial adhesion, and surface defects serving as charge separation centers. This morphology-dependent activity strongly suggests that future material design should prioritize hierarchical architectures over simple nanostructures.

Gram-positive bacteria (particularly *S. aureus*) are more susceptible to CdS-based antibacterial agents than Gram-negative bacteria (*E. coli*, *P. aeruginosa*). This differential susceptibility is attributed to fundamental structural differences in bacterial cell envelopes. The thick, porous peptidoglycan layer of Gram-positive bacteria (20–80 nm) lacks the protective outer membrane found in Gram-negative species, allowing for easier nanoparticle penetration and metal ion access. Additionally, the negatively charged teichoic acids in Gram-positive cell walls facilitate electrostatic attraction to cationic nanoparticles and Cd^2+^ ions. This consistent trend has important implications for practical applications, as it suggests that CdS-based materials will be more effective against Gram-positive pathogens and that strategies to overcome the Gram-negative outer membrane barrier (such as surface functionalization or ultrafine particle sizes) should be prioritized.

The photocorrosion of CdS represents the most significant fundamental limitation to practical implementation. Analysis of reuse cycle data reveals a clear hierarchy of protection effectiveness: S-scheme heterojunctions (10–15 cycles, 90–97% retention) > core–shell architectures (10–12 cycles, 85–94%) > Z-scheme systems (8–12 cycles, 80–95%) > polymer encapsulation (5–8 cycles, 70–80%) > Type II heterojunctions (5–8 cycles, 70–85%) > pristine CdS (1–2 cycles, 30–50%). This hierarchy reflects the distinct protection mechanisms operating in each system. S-scheme heterojunctions achieve exceptional stability through the formation of an internal electric field that selectively promotes recombination of low-energy charge carriers while preserving high-energy carriers, effectively suppressing the accumulation of holes at the CdS surface. A core–shell architecture provides complete physical isolation from the aqueous environment while enabling charge transfer through carefully engineered interfaces. Carbonaceous supports such as rGO offer dual protection through electron extraction (reducing hole concentration) and physical shielding.

Critically, the mechanism of photocorrosion involves stepwise oxidation of sulfide species: S^2−^ (161.5 eV) → S^0^ (163.5 eV) → SO_4_^2−^ (168 eV). This oxidation is dramatically accelerated in oxygenated aqueous environments and can proceed even in ostensibly coated systems if pinhole defects exist. The presence of oxygen shifts the corrosion pathway from elemental sulfur formation to sulfate formation: CdS + 4h^+^ + 2H_2_O + O_2_ → Cd^2+^ + SO_4_^2−^ + 4H^+^. This insight is crucial for understanding why even protective coatings can fail if they contain defects, and highlights the importance of developing conformal, defect-free protection strategies.

Cd^2+^ release kinetics show dramatic variation across material architectures, with cumulative release after 5 cycles ranging from <3% for S-scheme heterojunctions to >40% for unprotected quantum dots. This architectural dependence is critically important for environmental risk assessment, as the EPA maximum contaminant level for cadmium in drinking water is 5 µg/L. Systems with release rates exceeding this threshold are unsuitable for water treatment applications without post-treatment removal. Core–shell architectures (Fe_3_O_4_@CdS) and carbonaceous-supported systems (rGO/CdS) demonstrate the lowest release rates (<5% cumulative), due to physical encapsulation and surface shielding, respectively. S-scheme heterojunctions achieve similarly low release through efficient charge separation that reduces the hole concentration available for anodic sulfide oxidation [90,91,92,93,94,95,96,97,98,99,100,101,102,103,104,105,106,107,108,109,110].

The architectural dependence of Cd^2+^ release kinetics is exemplified by comparing two prominent CdS-based composite designs: core–shell Fe_3_O_4_@CdS nanohybrids and rGO-wrapped CdS quantum dots. Samadi-Maybodi et al. [101] synthesized magnetically separable Fe_3_O_4_@CdS type II core–shell nanohybrids where the CdS shell (12.6 nm crystallite size) completely encapsulates the Fe_3_O_4_ core (9.3 nm). This core–shell architecture creates a physical barrier that significantly retards Cd^2+^ ion release while maintaining photocatalytic activity through type II charge transfer, where photogenerated electrons are confined to the magnetite core and holes to the CdS shell, as confirmed by quenching experiments with electron (NO_3_^−^) and hole (4-methoxyphenol) scavengers [101]. The enhanced stability of this architecture is evidenced by the nanohybrids retaining approximately 94% of their antimicrobial efficacy after four cycles and one month of ambient storage [101]. In contrast, Afreen et al. [94] demonstrated that rGO-wrapped CdS quantum dots (2–3 nm crystallite size) exploit the impermeable graphene oxide layers to physically isolate the CdS surface from the aqueous environment. The rGO sheets serve as electron acceptors and transporters, efficiently reducing electron–hole recombination and simultaneously providing physical protection against photocorrosion [94]. The photocatalytic stability is markedly enhanced, with the rGO/CdS QD heterostructure retaining over 99% of its photodegradation efficiency after four consecutive cycles [94]. The key distinction between these architectures lies in their protection mechanisms: core–shell structures rely on complete physical encapsulation and interfacial charge transfer, while rGO wrapping combines electron extraction with surface shielding. Unprotected nanoparticles or simple binary mixtures, by contrast, exhibit rapid and substantial ion release, often exceeding regulatory limits for environmental discharge and biomedical use. These architectural differences fundamentally determine the risk–benefit profile of CdS-based materials and must be systematically evaluated for each proposed application.

The mechanism of Cd^2+^ release is not simply passive dissolution but is intimately connected to photocorrosion processes. In unprotected systems, photogenerated holes oxidize surface sulfide ions, creating Cd^2+^ vacancies that facilitate further dissolution. This autocatalytic degradation pathway explains why release accelerates with continued illumination. Conversely, in systems with efficient hole consumption (heterojunctions) or physical protection (coatings), the rate of sulfide oxidation is substantially reduced, and ion release follows a slower, diffusion-controlled mechanism. This mechanistic understanding provides a rational basis for selecting protection strategies: materials that efficiently consume holes or physically isolate the CdS surface will exhibit the lowest ion release [60,61,62,63,64,65,66,67,68,69,70,71,72,73,74,75,76,77,78,79,80,81,82,83,84,85,86,87,88,89].

The translation of CdS-based antibacterial materials from laboratory demonstrations to practical applications faces three primary challenges: cadmium toxicity, photocorrosion instability, and scalability. Regulatory frameworks in major markets impose stringent limits on cadmium release: the EPA maximum contaminant level is 5 µg/L for drinking water, the EU REACH regulation classifies cadmium as a Substance of Very High Concern requiring authorization, and the FDA requires demonstration of no acute or chronic toxicity for biomedical applications. Currently, only systems with cumulative release <10% (S-scheme heterojunctions, core–shell architectures, carbonaceous composites) meet these requirements for environmental applications. For biomedical applications, additional biocompatibility data are required, including hemolysis (<5%), cytotoxicity (LC_50_ > 10 µg/mL), and genotoxicity assessments.

**Figure 10:**
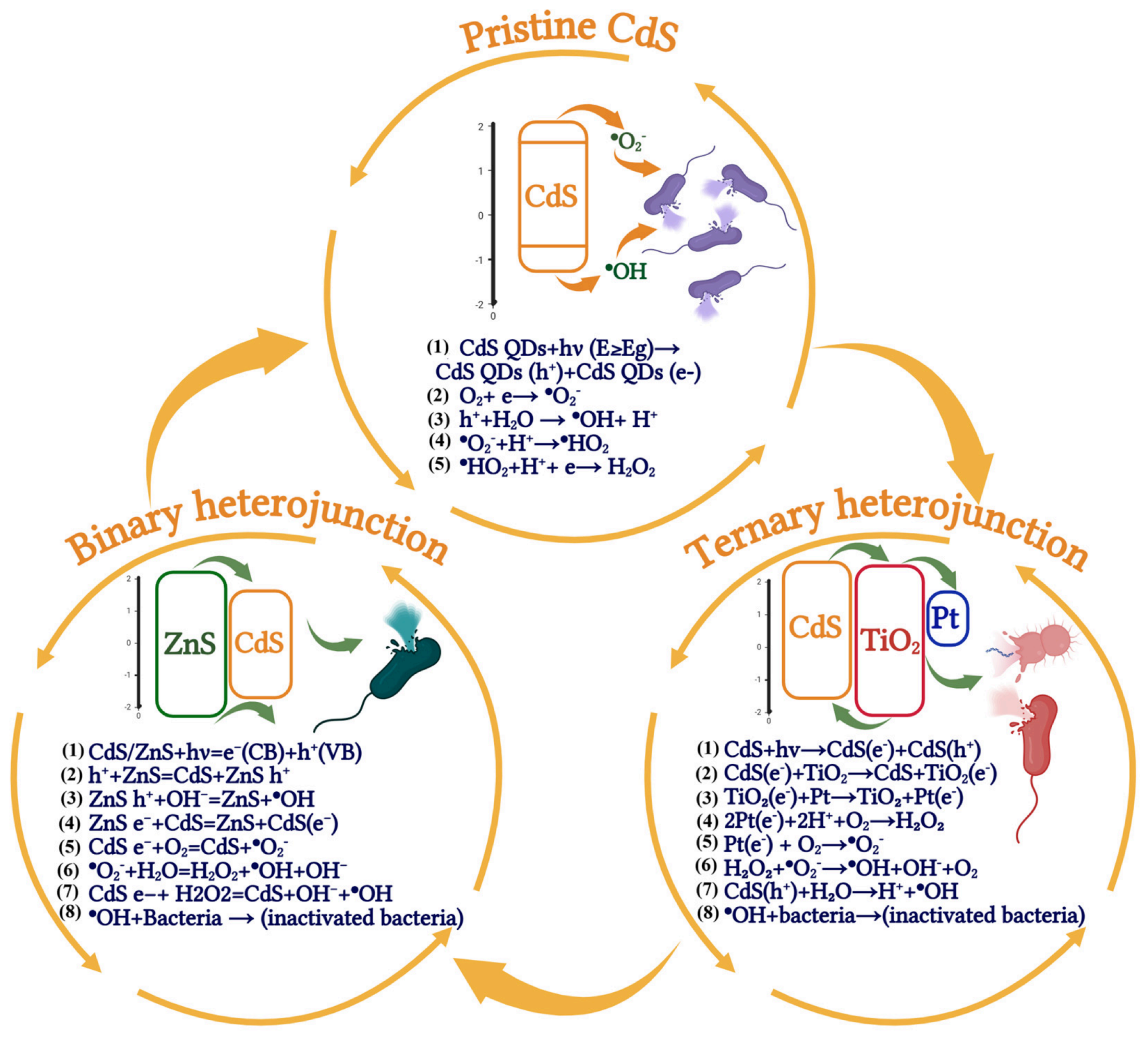
Evolution of photocatalytic antibacterial reaction pathways and charge transfer mechanisms in pristine CdS and CdS-based heterostructures. Created in BioRender. Tugelbay, S. (2026); https://BioRender.com/4nn785w, accessed on 8 July 2026.

As discussed in Section 2.5, cadmium toxicity poses the most significant barrier to clinical translation, with Cd^2+^ release kinetics critically dependent on composite architecture (Figure 10). Biogenic synthesis routes utilizing plant extracts, fungal cultures, and marine bacteria yield CdS with enhanced biocompatibility due to surface capping by phytochemicals or proteins, achieving acceptable hemocompatibility (<5% hemolysis) at therapeutic concentrations. However, systematic comparative studies with chemically synthesized controls and long-term nanotoxicological assessments remain necessary to establish comprehensive safety profiles.

Scalability presents an additional translational challenge that requires careful consideration of synthesis route selection. Mechanochemical synthesis offers inherent scalability advantages, including solvent-free operation, rapid processing (20–60 min), and minimal waste generation, making it economically attractive for industrial production. However, precise morphological control and phase purity can be compromised compared to solvothermal methods, as the high-energy ball milling process tends to produce more isotropic nanoparticles rather than anisotropic structures like nanorods or nanosheets. SILAR and chemical bath deposition are suitable for coating applications (e.g., antimicrobial implant surfaces) but suffer from slow deposition rates and limited batch-to-batch reproducibility, hindering large-scale adoption. Hydrothermal and solvothermal methods, while offering excellent morphological control, require high-pressure reactors and prolonged reaction times, substantially increasing equipment costs and energy consumption. Green synthesis routes using plant extracts or microbial cultures are environmentally benign and inherently scalable, but variability in biological materials poses challenges for consistent product quality. Notably, mechanochemical and green synthesis routes show the greatest promise for industrial translation, with estimated production costs significantly lower than conventional solvothermal approaches, though standardized protocols and quality control measures remain underdeveloped.

To contextualize the advantages and limitations of CdS, comparison with other frequently used sulfide semiconductors (ZnS, Ag_2_S, Bi_2_S_3_) is essential. ZnS (bandgap ~3.6 eV) offers excellent stability and no toxicity concerns but is limited to UV excitation, severely restricting its practical applicability. Ag_2_S (bandgap ~1.2 eV) provides NIR activity and good stability but exhibits limited ROS generation and high cost. Bi_2_S_3_ (bandgap ~1.3–1.7 eV) offers broad visible-light absorption and moderate stability but limited charge separation efficiency [1,44].

CdS occupies a unique position in this landscape: it provides the optimal bandgap for visible-light activity (2.4 eV), superior charge separation through its favorable band edge positions, and unparalleled morphological versatility. The critical limitation of CdS—photocorrosion and toxicity—can be substantially mitigated through heterojunction engineering and protective coatings, whereas the fundamental limitations of other sulfides (ZnS: UV-only activity; Ag_2_S: limited ROS; Bi_2_S_3_: poor charge separation) are more difficult to overcome. Recent studies on ZnS/Ag_2_S heterostructures and ZnS/Bi_2_S_3_ systems demonstrate that while these alternative sulfides offer improved stability, they sacrifice the photocatalytic efficiency achievable with CdS-based systems [1,44]. The strategic advantage of CdS is thus its exceptional photocatalytic potential, which, when protected through appropriate engineering, can outperform alternative sulfide systems.

A critical limitation that must be acknowledged when interpreting the comparative data presented in Table 2, Table 3 and Table 4 is the absence of standardized testing protocols across the published literature. The antibacterial performance of CdS-based nanomaterials is evaluated using a wide range of experimental conditions, including varying bacterial strains (e.g., different *E. coli* or *S. aureus* isolates), inoculum sizes (ranging from 10^4^ to 10^9^ CFU/mL), irradiation sources (Xe lamps, Hg lamps, solar simulators, sunlight), light intensities, exposure times, catalyst concentrations, and assay methods (disk diffusion, well diffusion, broth dilution, colony counting). Consequently, direct quantitative comparison of inhibition zones, MIC values, and bacterial inactivation efficiencies across different studies is inherently problematic. For instance, a ZOI of 25 mm reported under one set of conditions cannot be directly compared to a ZOI of 20 mm from another study using different bacterial strains or light sources. This lack of standardization is reflected in the incomplete reporting of experimental parameters, with many studies failing to specify light intensity, irradiation wavelength, or whether dark controls were performed. Furthermore, several papers in the current literature do not report quantitative data such as ZOI values, MIC/MBC values, or efficiency percentages, instead providing qualitative descriptions of antibacterial activity. To address this limitation, the present review has retrieved and reported all available quantitative data from the original literature; however, readers are urged to exercise caution when making cross-study comparisons and to consider the experimental conditions under which each reported value was obtained. The development of standardized testing protocols for photocatalytic antibacterial materials, including recommendations for bacterial strains, inoculum size, light source specifications, and reporting requirements, is urgently needed to enable meaningful benchmarking and accelerate the translation of promising CdS-based systems toward practical applications.

**Figure 11:**
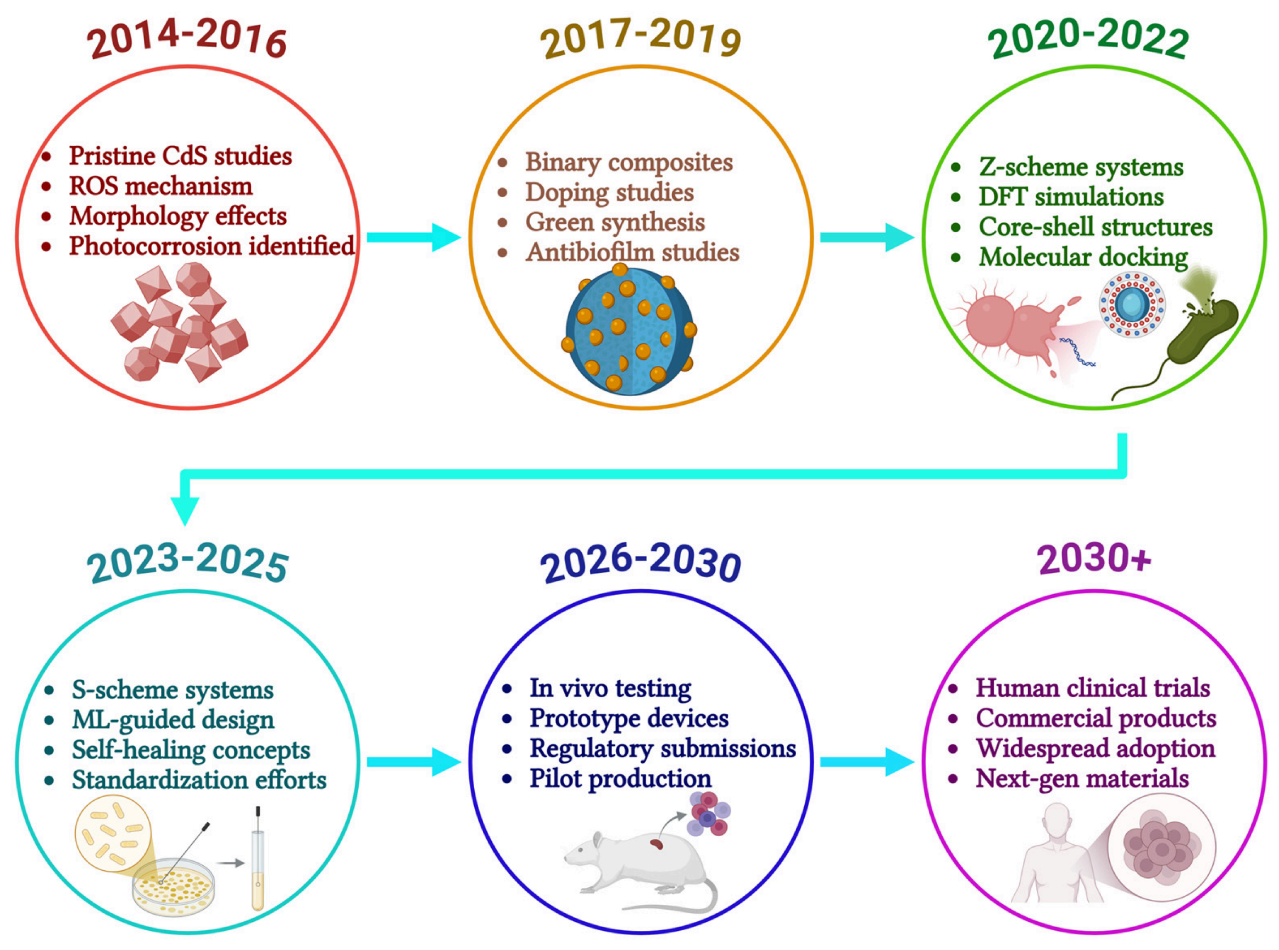
Evolution and future roadmap of CdS-based antibacterial nanomaterials from fundamental studies to clinical translation. Created in BioRender. Tugelbay, S. (2026); https://BioRender.com/z04tt2z, accessed on 8 July 2026.

Future perspectives should prioritize the following (Figure 11): (1) the development of self-healing protective coatings that regenerate under operational conditions; (2) rigorous, standardized nanotoxicological assessment of cadmium release kinetics, speciation, and chronic exposure effects; (3) integration with complementary modalities (photothermal therapy, magnetic targeting, controlled drug release) for synergistic multimodal platforms; and (4) machine learning-guided discovery of optimal compositions and heterojunction configurations. The convergence of morphological control, heterojunction engineering, and computational design offers unprecedented opportunities for rational optimization of CdS-based nanomaterials in the global fight against antimicrobial resistance.

## 11. Conclusions

This review has systematically examined the use of CdS for multimodal antibacterial therapy. The collective evidence establishes that appropriately engineered CdS exhibits potent, broad-spectrum antibacterial activity against pathogens, including multidrug-resistant strains. The fundamental advantages of CdS, its visible-light-responsive bandgap, favorable band edge positions, and ability to generate ROS, position it as a promising photocatalyst among inorganic semiconductor materials.

The critical analysis reveals that antibacterial efficacy is governed by structural and morphological parameters. Zero-dimensional quantum dots exhibit enhanced membrane penetration, while three-dimensional hierarchical architectures provide high surface areas and abundant active sites for bacterial interaction, consistently outperforming lower-dimensional counterparts. Heterojunction formation represents an effective strategy for overcoming intrinsic limitations, with binary and ternary composites demonstrating 2–5× enhancement in antibacterial activity compared to pristine CdS. Z-scheme and S-scheme heterojunctions emerge as optimal design paradigms, maintaining strong redox potential while facilitating spatial charge separation and achieving complete bacterial inactivation.

Elemental doping enables systematic modulation of electronic structure, with transition metal incorporation reducing bandgaps and transforming Gram-selective activity into broad-spectrum antimicrobial efficacy. Green synthesis routes utilizing plant extracts and fungal cultures yield nanoparticles with enhanced biocompatibility due to surface capping by phytochemicals, addressing toxicity concerns. Atomistic modeling, from DFT calculations of interfacial charge transfer to molecular docking of enzyme inhibitor interactions, provides valuable mechanistic insights that guide rational design.

Despite significant advances, the current literature remains predominantly limited to in vitro laboratory demonstrations. Three major limitations including photocorrosion, cadmium toxicity, and scalability, must be addressed through continued research. Future efforts should prioritize protective coating strategies, rigorous nanotoxicological assessment of Cd^2+^ release, and systematic in vivo evaluation. The convergence of morphological control, heterojunction engineering, elemental doping, and computational design offers opportunities for rational optimization. With continued interdisciplinary collaboration, CdS-based photocatalysts may fulfill their promise as effective antimicrobial agents.

## Figures and Tables

**Table 1:** Comparison of type II, Z-scheme, and S-scheme heterojunctions, highlighting their charge transfer mechanisms, redox capability, major advantages and limitations, and representative CdS-based antibacterial photocatalysts.

Heterojunction	Charge Transfer	Redox Ability	Main Advantage	Main Limitation	Representative Antibacterial Example
Type II	Electrons and holes migrate to different semiconductors	Reduced	Excellent charge separation	Lower ROS generation due to weakened redox potential	CdS/TiO_2_
Z-scheme	Low-energy electron–hole pairs recombine; high-energy carriers retained	High	Strong oxidation and reduction capability	More complex interface design	CdS/g-C_3_N_4_
S-scheme	Internal electric field drives recombination of weak carriers while preserving strong carriers	Highest	Combines efficient separation with maximum redox power and improved photocorrosion resistance	Relatively new mechanism requiring careful band alignment	CdS/BiVO_4_, CdS/COFs

**Table 2:** Comparative antimicrobial performance of pure CdS nanomaterials against microorganisms.

Composite	Activity	Target	Initial Conc.	Vol.	ZOI (mm)	Incubation Time (h)	Unique Features
CdS as QD [67]	Antibacterial, antifungal	*E. coli* (Gram-negative), *S. aureus* (Gram-positive), *A. flavus* (Fungus)	1–10 mg/mL (50 µL, CdS)	100 µL (bacteria)	8.5–25.5 mm	24 h	CdS synthesized at 50 °C with a 1:0.5 Cd:S ratio and PEG/PVPP surfactants demonstrated potent antimicrobial activity.
CdS [68]	Antibacterial and Antifungal	*B. subtilis*, *S. aureus*, *E. coli*, *Salmonella*, *C. albicans*	0–100 µg/mL (CdS)	Initial bacteria culture of 400 cells, diluted to 10^6^ CFU/mL	Agar plate antimicrobial activity of CdS was shown, but no ZOI value was found.	24 h	Moderate activity: Limited to Cd^2+^ ion penetration mechanism.
CdS as QDs [69]	Antibacterial	*S.aureus* (Gram-positive), *E. coli* (Gram-negative)	5–20 µg/mL (CdS)	Bacteria cultures were grown in 20 mL of nutrient broth and subjected to serial dilution	Number of bacteria colonies was dropped with the increase in the concentration of CdS	24 h	CdS in glass matrix generate ROS via light-induced electron–hole pairs.
CdS as nanoparticles [70]	Antibacterial	*E. coli K12*	Concentration is not specified, but 0, 50, 100, 150, 200, and 250 μL, volume of CdS used	20 μL (bacteria)	10^4^–10^9^ CFU/mL (bacteria survival count)	18–20 h	CdS (15 nm) induce 50% more ROS, 5× lipid peroxidation, reducing *E. coli* survival.
CdS as nanoparticles [71]	Antibacterial	*S.aureus* (Gram-positive), *E. coli* (Gram-negative), *B. subtilis* (Gram-positive), *P. vulgaris* (Gram-negative), *C. albicans*, *A549* (Lung cancer), *MCF7* (Breast cancer), *PC3* (Prostate cancer)	Fixed volume (exact volume not specified)	100 µL (10^5^ CFU/mL) of the bacterial and yeast cultures spread on agar plates	14–30 mm	24 h	Greater effect on Gram-positive bacteria than Gram-negative, Also evaluated for anticancer activity (IC50: 149–246 µg/mL against MCF7, PC3, A549), No antifungal activity detected against *Candida albicans*
CdS as nanoparticles [72]	Antibacterial	*E. coli* (Gram-negative)	100–1000 µg/mL (CdS)	Bacterial inoculum (1.5 × 10^8^ CFU/mL) was swabbed onto surface of the agar plate.	12–30 mm	24 h	CdS synthesized at 160–180 °C showed strong, concentration-dependent activity against *E. coli* and *S. aureus*.
CdS as nanoparticles [73]	Antibacterial	*B.subtilis* (Gram-positive), *K. pneumoniae* (Gram-negative), *P. aeruginosa* (Gram-negative), *S. aureus* (Gram-positive)	CdS were dropped onto filter paper	100 μL of bacterial suspension (10^7^ cells/mL)	Minimum–highest ZOI	48 h	CdS as thin films resisted bacterial disruption, maintaining optical, structural, and electrical properties.
CdS [74]	Antibacterial	*S. aureus ATCC 6538-P*, *B. subtilis ATCC 6633*, *E. faecium ATCC 700221*, *S. pneumoniae ATCC BAA-660*, *S. epidermidis ATCC 11228*, *K. pneumoniae ATCC 10031*	10 mg/mL (stock suspension) (Serial dilution method), 100 µL of CdS sample (Diffusion method)	1.5 × 10^8^ CFU/mL (for diffusion method and stock inoculum) 1.5 × 10^6^ CFU/mL (for serial dilution method after 100× dilution)	~6–33 mm	24 h	Mechanochemically synthesized CdS (5–6 nm) showed superior antibacterial activity against 13 strains, with MBC as low as 0.04 mg/mL for *S. aureus*.
CdS as nanoparticles (with Schiff base) [75]	Antibacterial	*P. aeruginosa* (Gram-negative), *S. aureus* (Gram-positive), *E. coli* (Gram-negative), *S. aureus*	Not stated	100 μL of bacteria culture	6.09–25.6 mm	24 h	SB-capped CdS (10 μL) showed effective antibacterial activity against *E. coli*, *S. aureus*, and *P. aeruginosa*.
CdS as nanoparticles [76]	Antibacterial, antifungal	*Streptococcus sp.* (Gram-positive), *Staphylococcus aureus* (Gram-positive), *Escherichia coli* (Gram-negative), *Bacillus subtilis* (Gram-positive), *Klebsiella pneumoniae* (Gram-negative), *Collatorichum dematium* (Fungus), *Alternaria alternata* (Fungus), *Fusarium roseum* (Fungus), *Curvularia lunata* (Fungus), *Aspergillus flavus* (Fungus)	125–1000 µg/mL	Bacteria culture was prepared in a media of 250 mL distilled water	17.1–31.06 mm for antibacterial case, 6.8–77.8 mm for antifungal case	24 h for antibacterial case, 72 h for antifungal case	The green-synthesized CdS-LA hybrid nanoparticles capped with *Lathyrus aphaca* L. extract exhibited potent, dose-dependent antimicrobial activity, with the highest inhibition observed against *Streptococcus* species (31.06 mm) among bacteria and *Collatorichum dematium* (77.8% inhibition) among fungi, attributed to the synergistic effects of the nanoparticles and surface-bound phytochemicals.
CdS [77]	Antibacterial	*S. aureus*, *E. coli*	6.5–100 µg/mL	Bacteria were inoculated at 1:500 ratio	Agar plate antimicrobial activity of CdS was noted	24 h toxicity test: 24 h Disk diffusion assay: 18 h Respiration monitoring: 4 h (after 2–3 h pre-incubation)	Two mechanochemical routes (acetate and combined milling) produced CdS with enhanced photocatalysis and antibacterial activity.
CdS as QDs [78]	Antibacterial	*E. coli* (Gram-negative), *B. subtilis* (Gram-positive), *MCF-7* (Breast cancer)	10–100 µg/mL (CdS)	Plates containing nutrient broth medium were used for bacteria culture	12.5–14.5 mm	12 h	Green synthesis of CdS using saffron extract showed 92% dye degradation and 79% cancer cell apoptosis.
CdS as nanoparticles [79]	Antibacterial, Antifungal	*Klebsiella planticola* (Gram-negative), *Vibrio sp.* (Gram-negative), *Planomicrobium* sp. (Gram-positive), *Serratia nematodiphila* (Gram-negative), *Escherichia coli* (Gram-negative), *Aspergillus flavus* (Fungus), *Aspergillus niger* (Fungus)	50, 100, and 200 μL (volume added CdS)	20 mL of molten and cooled Muller Hinton agar	6.20–23.27 mm	24 h (antibacterial) 48 h (antifungal)	CdS (50–200 μL) showed concentration-dependent antibacterial and antifungal activity against multiple pathogens.
CdS as nanoparticles [80]	Antibacterial, Biocompatibility, Hemolysis	*S. aureus* (Gram-positive), *A. baumannii* (Gram-negative), *E. coli* (Gram-negative), *Human RBCs*	25–100 μL of CdS (antibacterial test), 2–10 mg/mL (hemolysis)	Wells of 8 mm were made on the nutrient agar plates.	~15–25 mm	24 h (antibacterial) 1 h (hemolysis)	CdS (2.38 eV) achieved 95% dye degradation and showed good antibacterial activity against *S. aureus*.
CdS as nanoparticles [81]	Antibacterial	*B. cereus* (Gram-positive), *E. coli* (Gram-negative)	50–1000 µg/mL	10 µL of diluted bacterial suspension (~5 × 10^4^ CFU per well)	4–33 mm	24 h	Solvothermal CdS (11.9–41.3 nm) show strong antibacterial (MIC 100 μg/mL) and antibiofilm activity.
CdS as nanoparticles [82]	Antibacterial	*E. coli* (Gram-negative), *P. aeruginosa* (Gram-negative), *S. aureus* (Gram-positive), *B. subtilis* (Gram-positive)	60–100 µg/mL	100 µL of each bacteria culture	~8–18 mm	24 h	CdS (5 nm) from cinnamon extract show 94% sunlight dye degradation and anticancer activity.
CdS (Nostoc carneum extract) [83]	Antibacterial, and dye degradation	*Klebsiella pneumoniae* (Gram-negative), *Pseudomonas aeruginosa* (Gram-negative), *Staphylococcus aureus* (Gram-positive), *Escherichia coli* (Gram-negative)	10–40 μg/mL	Not stated	~6–12 mm	24 h	CdS as QD (2–6 nm) from cyanobacteria show >90% dye degradation and significant antibacterial activity.
CdS [84]	Antibacterial, Anticancer (MCF-7)	*Human breast cancer cells* (MCF-7), *B. subtilis*, *S. aureus*, *E. coli*, *K. pneumoniae*	60–100 µg/mL (CdS) for antibacterial study	100 μL of bacteria culture	~8–35 mm for ZOI of antibacterial study	48 h for anticancer case, 24 h for antibacterial case	Bioactive compounds (terpenes, gingerols) enhance bacterial cell interaction.
Various shaped CdS [85]	Antibacterial	*S. aureus* (Gram positive), *B. subtilis* (Gram positive), *E. aerogenes* (Gram negative), *E. coli* (Gram negative)	0.01–0.0331 ppm	50 μL of solutions and antibiotic	~9–35 mm	24 h	CdS morphology shifts from spherical to rose-like with higher TEA, boosting photocatalytic dye degradation and antibacterial activity.
CdS as thin films (100 nm) [86]	Antibacterial	*Staphylococcus aureus* (Gram positive), *Pseudomonas aeruginosa* (Gram negative), *Escherichia coli* (Gram negative)	10 μL	9 mL of sterile nutrient broth in test tubes and inoculated with 0.1 mL of bacterial suspension	No stated	24 h	CdS as thin films (50–100 nm) showed strong antibacterial activity against Gram-positive and Gram-negative bacteria under sunlight.
C. gigantea–CdS NPs [87]	Antibacterial, antifungal	*E. coli* (Gram negative), *P. aeruginosa* (Gram negative), *S. aureus* (Gram positive), *B. thuringiensis* (Gram positive), *A. niger*, *C. albicans*	Not stated	100 µL (antibacterial), not stated for antifungal case	~1–40 mm	24 h (antibacterial), 48 h for antifungal case	Green CdS as nanoparticles (20 nm) showed effective antibacterial, antifungal, and photocatalytic dye degradation with metal sensing.
CdS (Tea leaf extract) [88]	Antibacterial and others	*Serratia marcescens* (Gram negative), *Staphylococcus aureus* (Gram positive), *Klebsiella pneumoniae* (Gram negative), *Streptococcus pyogens* (Gram positive), *Escherichia coli* (Gram negative)	40–200 µg/mL	Not stated	9–18 mm	24 h	Green CdS (3–5 nm) showed antibacterial, bioimaging, and lung cancer cell apoptosis effects.

**Table 3:** Antibacterial activity and distinct features of CdS/X binary composites under various light conditions.

Composite	Activity	Target	Initial Conc.	Vol. (Bacteria)	ZOI (mm)	Incubation Time (h)	Unique Features
CdS-Ag_2_S (PVP-capped) [89]	Antibacterial	*E. coli*, *P. aeruginosa*, *S. aureus*	Not specified	Not specified	18.34–39.24 mm	24 h	Antibacterial activity was significantly improved due to in creasing ratio of CdS into CdS-Ag_2_S nanocomposites
CNC-g-PVP/CdS [90]	Antibacterial	*S. aureus*, *E. coli*	Not specified	Bacterial solutions comprising 1.5 × 10^8^ CFU mL^−1^	1.05–5.25 mm	24 h	CdS with natural and synthetic polymers could be environmentally friendly, economical, and effective against bacteria and industrial dye degrader.
CdS@CTN QDs [91]	Antibacterial	*E. coli*, *B. subtilis*, *S. aureus*, *P. aeruginosa*	2.0 mg/mL	200 μL of bacterial culture	Not stated	24 h	CdS@CTN QDs showed no antibacterial activity, indicating chitin encapsulation and suggesting good biocompatibility pending testing.
CZ3 (1–3% ZrO_2_-CdS) [92]	Antibacterial	*S. aureus* (Gram-positive), *E. coli*	25–100 μg/mL	In sterile test tubes, 2 mL of bacterial suspension was mixed with 1 mL of nanoparticle solutions	12–20 mm	24 h	Green-synthesized zirconia-doped CdS show reduced particle size and enhanced photocatalytic, antibacterial, and antioxidant activities.
CdS/Ag_2_S NCs [93]	Antibacterial	*E. coli*, *S. aureus*	0.1–10 mg/L	Not specified	ZOI not specified, 79–99% inhibition	24 h	CdS/Ag_2_S nanocomposites showed strong visible-light antibacterial and antibiofilm activity via enhanced ROS generation and photocatalysis.
rGO/CdS QD [94]	Antibacterial	*S. aureus*, *E. coli*,	25–100 μg/mL	200 μL	~5–25 mm	24 h	rGO/CdS quantum dot heterostructure shows enhanced visible-light photocatalysis and antibacterial activity via efficient ROS generation.
TiO_2_/CdS NC [95]	Antibacterial	*E. coli*	45, 55 μg/mL	100 μL	~8–16 mm	24 h	Highest activity among tested samples: Better than pure CdS (13.30 mm) and pure TiO_2_ (13.99 mm) at same concentration.
CdS/CuO NC (5%) [96]	Antibacterial (Colony count)	*E. coli* (Gram-negative)	30 mg/mL	Bacteria growth condition: 1.5 × 10^8^ CFU mL^−1^	16.5–19.2 mm	24 h	Highest antibacterial activity among tested samples: Much better than pure CdS (30.88%) and pure CuO (39.16%). Confirms synergy in heterojunction.
CdS-Nb3 (1–3% Nb-doped CdS) [97]	Antibacterial	*S. aureus*, *E. coli*	25–100 mg/mL	Not stated	~5–21 mm	24 h	Activity increases with Nb doping concentration. Good activity against Gram-negative, though lower than against *S. aureus* due to outer membrane barrier.
CdS/TZ (CdS/TiO_2_) [98]	Antibacterial	*E. coli*, *Enterococcus faecium* (Gram-positive), *P. aeruginosa*	Not specified	20 mL bac terial solution, 100 µL solution was withdrawn at specific time intervals	4.52–20.36 mm	24 h	Highest antibacterial activity: Comparable to positive control Amikasin (24 ± 0.24 mm). Activity increases with CdS content (CdS/TZ 3 > CdS/TZ 2 > CdS/TZ 1 > TZ).
CdS:ZnO [99]	Antibacterial	*Candida* (Fungus), *E. coli*, *S. aureus*, *Klebsiella* sp., *S. epidermidis*, *Candida*	Not specified	Not specified	15–40 mm	24 h	Highest antifungal activity: best among all tested samples and ratios. Synergistic effect of ZnO and CdS.
PANI-CdS [100]	Antibacterial	*S. aureus* (Gram-positive), *P. mirabilis* (Gram-negative), *E. coli*, *K. pneumoniae*	Not specified	Bacteria growth condition: 1.5 × 10^8^ CFU mL^−1^	0–37 mm	24 h	PANI–CdS showed strong dose-dependent broad-spectrum antibacterial activity via synergistic ROS generation and Cd^2+^ release.
Fe_3_O_4_@CdS [101]	Antibacterial	*S. saprophyticus*, *E. coli*,	3–7.0 mg/mL	1.4 × 10^8^ and 1.2 × 10^8^ CFU mL^−1^	5–15.7 mm	16 h	Fe_3_O_4_@CdS core–shell nanohybrids exhibit enhanced antibacterial activity via efficient ROS generation and magnetic recoverability.
CdS@ZIF-8 (Ship-in-the-bottle) [102]	Antibacterial	*E. coli*, *B. subtilis*, *S. aureus*	Not stated	Not stated	12.2–35.10 mm	24 h	CdS@ZIF-8 nanocomposites show strong dose-dependent antibacterial activity, influenced by encapsulation strategy and metal ion interactions.
Fe-doped ZnO/CdS (2.0 wt% Fe) [103]	Antibacterial	*E. coli*, *S. aureus*, *K. pneumoniae*, *P. aeruginosa*	Not stated	Not stated	3–20 mm	Overnight	Fe-doped ZnO/CdS exhibit enhanced dose-dependent antibacterial activity through ROS generation and improved structural properties.
Cu:CdS [104]	Antibacterial	*Serratia marcescens*, *Streptococcus*	1–8 mg/mL	Bacteria were inoculated in 25 mL of Luria Broth	7–19 mm	Overnight	Star-shaped Cu:CdS exhibit superior antibacterial activity against resistant bacteria due to sharp-edge membrane disruption.
ZnO-CdS [105]	Antibacterial	*E. coli*	0–25 mM (CdS)	Not specified	~5–20 mm	24 h	ZnO–CdS composites show enhanced photocatalytic and dose-dependent antibacterial activity via charge separation and metal ion interactions.
CdS/MCM-41/HNTs (Hierarchical mesoporous carrier) [106]	Photocatalytic Antibacterial	*S. aureus*, *E. coli*	63–1000 μg/mL	Bacteria growth condition: 1.5 × 10^8^ CFU mL^−1^	ZOI not mentioned, but 50–100% inhibition	18 h	CdS quantum dots on mesoporous carriers show strong visible-light antibacterial activity via efficient ROS generation.
Cd_0.96_Mn_0.02_Co_0.02_S [107]	Antibacterial	*E. coli* (Gram-negative), *S. aureus*	Not stated	Not stated	~31–32 mm	24 h	Mn,Co dual-doped CdS thin films exhibit enhanced antibacterial activity due to reduced crystallite size and morphology changes.
Zn + F doubly doped CdS, 2 wt% F [108]	Antibacterial	*E. coli*	Not stated	Not stated	~25–40 mm	24 h	(Zn + F) doubly doped CdS thin films show strong antibacterial activity via defect-induced ROS generation.
Mn-CdS [109]	Antibacterial	*E. coli & S. paratyphi A*	1–5 mg/mL (CdS)	Not stated	~7–11 mm	24 h	Mn-incorporated CdS exhibit enhanced broad-spectrum antibacterial activity due to Mn^2+^ ion-induced enzymatic disruption.
Au/CdS [110]	Antibacterial	*P. mirabilis*	400–4000 μg/mL	Not stated	2–15.33 mm	24 h	Au-decorated CdS exhibits enhanced antibacterial activity and suppresses ureC gene in *P. mirabilis*.
Cd_60_Ni_20_Cu_20_S [111]	Antibacterial, Antifungal	*E. coli*, *Lactobacillus* (Gram-positive), *Aspergillus flavus* (Fungus), *Aspergillus niger*	Not stated	Bacteria growth condition: 1–2 × 10^8^ CFU mL^−1^	22–29 mm	24 h	Ni-Cu co-doped CdS shows enhanced photocatalytic, antibacterial, antifungal activity, and tunable bandgap.
CdS: CTAB [112]	Antibacterial	*MRSA*	0.125–1024 μg/mL	Bacteria growth condition: 2 × 10^8^ CFU mL^−1^	8.46–8.74 mm	24 h	CTAB-capped CdS exhibits moderate antibacterial activity against MRSA, enhanced by small particle size and surface capping.
ZnNiNdO/CdS [113]	Antibacterial	*S. aureus*, *E. coli*, *P. vulgaris*, *P. aeruginosa*	CdS concentration is not stated	Bacteria column volume is not stated	5–40 mm	24 h	Z-scheme ZnNiNdO/CdS heterojunction shows enhanced visible-light photocatalysis and strong antibacterial activity via ROS generation.
D-CdS (DNA capped) [114]	Antibacterial	*Pseudomonas aeruginosa* (Gram negative), *Staphylococcus aureus* (Gram positive)	1000 μg/mL	Bacteria were cultured on 20 mL Muller Hinton agar plates	11–29 mm	24 h	Key point: DNA-capped CdS shows improved dispersion, strong photocatalytic dye degradation, and significant antibacterial activity.
CdS/Fe_3_O_4_ [115]	Antibacterial	*E. coli* (Gram negative), *Enterobacter aeruginosa* (Gram positive), *Klebsiella pneumoniae* (Gram negative), *Pseudomonas aeruginosa* (Gram negative)	0.001–0.025 g/mL	300 μL of microbe’s cultures	17–26 mm	24 h	Green-synthesized CdS/Fe_3_O_4_ nanocomposites show enhanced photocatalytic dye degradation and strong antibacterial activity via ROS generation.
CdS/TiO_2_ [116]	Larvicidal	*Culex pipiens larvae* (III instar)	0.156–2.5 mg of CdS in 200 μL	Not stated	0–35 mm	24 h	CdS/TiO_2_ nanocomposite shows enhanced photocatalytic degradation, strong antibacterial activity, and effective larvicidal performance.
Ag/CdS [117]	Antibacterial	*Staphylococcus aureus* (Gram positive), *Escherichia coli* (Gram negative)	10–200 μg/mL	Not stated	6–16 mm	24 h	Ag-doped CdS nanocrystals exhibit enhanced antibacterial activity against *S. aureus*, but ineffective against *E. coli*.
Pc/CSNC (Pectin-CdS) [118]	Antibacterial	*Escherichia coli* (Gram negative)	50–100 μg/mL	10 mL of the dilute culture	14–27 mm	12 h	Pectin-capped CdS nanocomposite shows efficient photocatalytic dye degradation and strong antibacterial activity against *E. coli*.
GO-CdS [119]	Photocatalytic, Antibacterial (Disinfection)	*Escherichia coli* (Gram negative), *Bacillus subtilis* (Gram positive)	5 mg of CdS composites added to 50 mL bacteria solution	50 mL, bacteria growth condition: ~10^7^ CFU/mL	~100% kill	24 h	GO–CdS composites enable efficient visible-light photocatalysis and rapid bacterial inactivation via enhanced charge transfer.
TiO_2_/CdS-2 (Ti/Cd mass ratio 2) [120]	Antibacterial	*Escherichia coli* (Gram negative)	5 mg of CdS composites added to 50 mL bacteria solution	50 mL, bacteria growth condition: ~10^8^ CFU/mL	~99.9% kill	24 h	Hierarchical TiO_2_/CdS composites show enhanced visible-light photocatalysis and rapid bacterial inactivation via efficient heterojunction charge transfer.
CZ1:3 (CdS/ZnO 1:3) [121]	Antibacterial	*Staphylococcus aureus* (Gram positive), *Escherichia coli* (Gram negative), *Klebsiella pneumoniae* (Gram negative)	100 mg/mL (CdS)	250 μL	8–30 mm	24 h	CdS/ZnO nanocomposites show morphology-dependent photocatalysis and antibacterial activity, with interfaces and ZnO content enhancing performance.
ZC10 (10 SILAR cycles) [122]	Antibacterial	*Escherichia coli* (Gram negative)	Not stated	100 μL bacteria suspension	~100% kill efficiency	24 h	Optimized ZnO@CdS nanorods (10 SILAR cycles) achieved complete visible-light *E. coli* inactivation.
CdS:Zr (4 wt.%) [123]	Antibacterial	*Klebsiella pneumoniae* (Gram negative)	Not specified	Not specified	18–31 mm	Not specified	Zr-doped CdS thin films exhibited improved transparency, conductivity, ferromagnetism, and enhanced antibacterial activity against *Klebsiella pneumoniae*.
f-MWCNTs-CdS [124]	Antibacterial	*Escherichia coli* (Gram negative), *Pseudomonas aeruginosa* (Gram negative), *Staphylococcus aureus* (Gram positive)	20 μg sample/mL	20 μL, bacteria suspension	14.7–97.8% reduction in bacteria	1 h	PAMAM-grafted MWCNTs decorated with CdS or Ag_2_S QDs showed potent, synergistic antibacterial activity, especially against Gram-negative bacteria.
ZnO/CdS [125]	Antibacterial	*Staphylococcus aureus* (Gram positive)	5–11 μg/mL	Not specified	ZOI showed antibacterial effect of more pronounced against Gram (+) bacteria	24 h	ZnO/CdS exhibited enhanced, dose-dependent antibacterial activity against *Staphylococcus aureus* via synergistic ROS generation.
SiO_2_@CdS (75 μg/mL) [126]	Antibacterial	*E. coli*	25–500 μg/mL	Bacteria were collected and diluted to a cell density of 1 × 10^5^/mL	~95% killed	24 h	Mesoporous SiO_2_@CdS nanospheres exhibited high photocatalytic and concentration-dependent antibacterial activity against *E. coli* via ROS generation.
CdS/ZnS-180 (180 °C) [127]	Antibacterial	*E. coli*, *S. aureus*	50–250 μg/mL	Not specified	6.5–25 mm	24 h	CdS/ZnS-180 nanocomposites showed enhanced antibacterial activity via type I band alignment, ROS generation, and high surface area.

**Table 4:** Antibacterial activity and distinct features of CdS/X/X composites under various light conditions.

Composite	Activity	Target	Initial Conc.	Vol.	ZOI (mm)	Incubation Time (h)	Unique Features
Cr-CdS@SCN (S-g-C_3_N_4_/Cr-doped CdS) [128]	Antibacterial, Antioxidant, Enzyme Inhibition	*E. coli* (Gram-negative), *S. aureus* (Gram-positive), DPPH Radical, Urease Enzyme	0.5–2.0 mg/mL	Not stated	13–23 mm	24 h	Cr-doped CdS/g-C_3_N_4_ nanocomposite shows strong antibacterial, antioxidant, urease inhibition, and photocatalytic dye degradation performance sunlight.
TiO_2_/CdS/PbS (p-SILAR, hierarchical) [129]	Antibacterial	*E. coli* (Gram-negative)	Not stated	100 μL/100 mL of bacteria inoculum	5–15 mm	24 h	TiO_2_/PbS/CdS hierarchical nanocomposite exhibits enhanced antibacterial activity via ROS generation from efficient band-aligned electron transfer.
BC-DMF [130,131]	Photocatalytic, Antibacterial	*E. coli* (Gram-negative), *S. aureus* (Gram-positive)	50 mg (CdS) in 100 mL bacterial solution	100 mL bacterial solution	12.01–93.09% (antibacterial efficiency)	10 min (>420 nm) (Irradiation time (min))	Bi_2_S_3_/CdS Z-scheme heterojunction nanoflowers show efficient visible-light antibacterial activity via enhanced charge separation and ROS generation.
cl-Ch-pMAc@ZnO/CdSQs2 [132]	Antibacterial	*B. subtilis* (Gram-positive), *E. coli* (Gram-negative)	4–18 mg/dish	10^7^ cells/mL (bacteria solution)	5–40 mm	24 h	Chitosan-supported ZnO/CdS quantum dot nanocomposite shows strong antibacterial activity via Zn^2+^ release and ROS generation.
BAC-60 (BiVO_4_ nanoarrays/CdS) [133]	Photocatalytic, Antibacterial	*E. coli* (Gram-negative)	Not specified	1.0 × 10^6^ CFU/mL (bacteria solution)	~98% inactivation	0–120 min	BiVO_4_/CdS Z-scheme heterojunction nanoarrays exhibit efficient visible-light antibacterial activity via enhanced charge separation.
NNO-C2 (NaNbO_3_/CdS) [134]	Pyrocatalytic, Antibacterial	*Salmonella typhimurium*, *Salmonella*	10 mg of NNO-C2 and 10 mg of CdS were dispersed in 49.5 mL of NaCl solution (85%)	100 μL (bacteria solution)	60–90% sterilization (degradation)	12 h	NaNbO_3_/CdS S-scheme nanorod composite shows efficient pyrocatalytic antibacterial activity via ROS generation during temperature cycles.
20% CdS@ZM [135]	Photocatalytic, Antibacterial	*S. aureus* (Gram-positive)	10 mg (CdS) in 40 mL saline	2.0 × 10^9^ CFU/mL (bacteria solution)	~100% survival rate	18 h	CdS@ZIF-67 S-scheme heterojunction shows rapid visible-light antibacterial activity via efficient charge separation and ROS generation.
Fe@CdS/SCN [136]	Antibacterial	*S. aureus* (Gram-positive), *E. coli* (Gram-negative)	Not specified	100 mL of the grown bacterial inoculum	5–20 mm	24–48 h (incubation time)	Fe-doped CdS/SCN nanocomposite exhibits enhanced antibacterial activity through improved charge separation and ROS generation.
PANI-CdS-ZnO-AP (15:85) [137]	Photocatalytic, Antibacterial	*Shigella* (Gram-negative), *S. aureus* (Gram-positive), *E. coli* (Gram-negative), *Streptococcus* (Gram-positive)	0.30 mg/L	Not specified	Antibacterial efficiency measured. ZOI not reported in the main text	24 h	PANI-supported CdS-ZnO-Ag_3_PO_4_ ternary heterojunction shows enhanced visible-light antibacterial activity via efficient ROS generation.
C_3_N_4_/CS-CdS [138]	Antibacterial	*S. aureus* (Gram-positive), *E. coli* (Gram-negative)	500–1000 μg/50 μL	1.5 × 10^8^ CFU/mL (bacteria solution)	0–4.35 mm	48 h	C_3_N_4_/CS co-doped CdS exhibit enhanced antibacterial activity via synergistic ROS generation and bacterial membrane disruption.
CdSe/CdS/NAC/Quercetin [139]	Antibacterial (MIC)	*Staphylococcus aureus* (Gram-positive), *Enterococcus faecalis* (Gram-positive), *Escherichia coli* (Gram-negative), *Pseudomonas aeruginosa* (Gram-negative)	1.57–25 mg/mL	200 μL	Antibacterial efficiency measured. ZOI not reported in the main text	12 h	CdSe/CdS/NAC/quercetin quantum dots exhibit strong antibacterial activity via ROS generation and quercetin-mediated membrane disruption.
Ag-PdS/ZnS/CdS (Reverse type I core/shell QDs with dual cocatalysts) [140]	Antibacterial (MIC/MBC)	*S. saprophyticus* (Gram-positive), *E. coli* (Gram-negative)	2.0–11.0 mg mL^−1^	1 mL	20–100% bacterial efficiency	18 h	Ag-PdS/ZnS/CdS reverse type I QDs exhibit enhanced photocatalytic antibacterial activity via dual-cocatalyst-driven ROS generation.
Pt-Ag_3_PO_4_/CdS/Chitosan [141]	Antibacterial	*S. aureus* (Gram-positive)	0–20 μg/mL	Not stated	~0–44 mm	24 h	Pt-Ag_3_PO_4_/CdS/chitosan nanocomposite exhibits strong visible-light antibacterial activity via Pt-assisted ROS generation and chitosan-mediated stabilization.
Ag-CdS/MWCNTs [142]	Antibacterial (Disk diffusion)	*S. aureus* (Gram-positive), *B. subtilis* (Gram-positive), *E. coli* (Gram-negative)	Not stated	50 μL	3.34–10.7 mm	Overnight	Ag-CdS/MWCNT nanocomposite shows enhanced antibacterial activity via ROS generation and efficient electron–hole separation on MWCNT support.
Cu@CdS [143]	Antibacterial, Photocatalytic degradation	*S. aureus* (Gram-positive), *E. coli* (Gram-negative), Ciprofloxacin (CIP) drug	50–200 mg/mL	Not stated	Antibacterial efficiency measured. ZOI not reported in the main text	24 h	g-C_3_N_4_/Cu@CdS nanocomposite exhibits antibacterial activity via Cu-induced charge separation and ROS generation under sunlight.
CdSe/CdS/ZnS [144]	Antibacterial	*P. aeruginosa* (Gram-negative), *K. pneumoniae* (Gram-negative)	Not stated	Not stated	2–20 mm	24 h	CdSe/CdS/ZnS core–multi-shell QDs exhibit antibacterial activity via ROS generation, enhanced by polymer encapsulation for stability.
ZnO (NR)-CdS-Ag [145]	Photocatalytic inactivation	*Escherichia coli*	0.1–0.5 g/L	100 μL	Complete inactivation. ZOI not reported in the main text	1 h of photocatalysis after 1 h of adsorption	ZnO-CdS-Ag ternary nanocomposites show enhanced photocatalytic antibacterial activity via nanorod-facilitated charge transfer and ROS generation.
CdS/Pt-TiO_2_ NTs [146]	Photoelectrocatalytic inactivation	*Escherichia coli*	Not stated	~5 × 10^8^ CFU/mL	~100% inactivation	24 h	CdS/Pt-TiO_2_ nanotube photoelectrode exhibits enhanced antibacterial activity via efficient electron transfer and ROS-mediated bacterial inactivation.
Ni/Ce-CdS (25 μL) [147]	Antibacterial	*Escherichia coli* (Gram negative), *Staphylococcus aureus* (Gram positive)	25–75 μL/mL	0.1 mL of cultivated bacteria	4–21.0 mm	24 h	Ni/Ce co-doped CdS exhibits enhanced antibacterial activity via bandgap narrowing, reduced recombination, and ROS-mediated membrane damage.

**Table 5:** Final summary of CdS-based antibacterial systems.

System Category	Representative Materials	Antibacterial Performance	Dominant Mechanism	Key Advantages	Primary Limitations	Comparison with Other Sulfides
Pristine CdS Nanostructures [1,44,67,68,69,70,71,72,73,74,75,76,77,78,79,80,81,82,83,84,85,86,87,88,119,130]	QDs (2–10 nm), NPs (10–50 nm), Nanorods, Nanosheets, Thin films	ZOI: 6–35 mm; MIC: 0.02–1000 µg/mL; *E. coli*: 10–99% kill; *S. aureus*: 10–99% kill	ROS generation (•O_2_^−^, ^1^O_2_); Cd^2+^ release; membrane disruption	Simple synthesis; direct bandgap (2.42 eV); visible-light active; morphological versatility	Rapid photocorrosion; high Cd^2+^ release (up to 38% after 5 cycles); moderate charge separation; limited stability (1–2 cycles)	vs. ZnS: CdS has narrower bandgap (2.4 vs. 3.6 eV) for visible-light activity; vs. Ag_2_S: CdS has stronger ROS generation; vs. Bi_2_S_3_: CdS has superior charge separation but lower stability
CdS Binary Composites [1,34,35,36,37,38,39,40,41,42,43,44,89,90,91,92,93,94,95,96,97,98,99,100,101,102,103,104,105,106,107,108,109,110,111,112,113,114,115,116,117,118,119,120,121,122,123,124,125,126,127]	CdS/TiO_2_, CdS/ZnO, CdS/Ag_2_S, rGO/CdS, CdS/MWCNTs, PANI-CdS	ZOI: 4–40 mm; MIC: 16–1000 µg/mL; *E. coli*: 70–99.9% kill; *S. aureus*: 53–99% kill	Type II charge transfer; enhanced ROS generation; synergistic ion release (Cd^2+^ + Ag^+^, Zn^2+^)	2–5× enhancement over pristine CdS; improved charge separation; reduced photocorrosion; added functionality (magnetic, conductive)	More complex synthesis; interface quality critical; limited stability (5–8 cycles); moderate Cd^2+^ release (10–20%)	vs. ZnS/Ag_2_S: CdS-based composites show higher ROS generation but lower stability; vs. ZnS/Bi_2_S_3_: CdS composites have better visible-light harvesting but faster degradation; all sulfide-based systems benefit from type II/interface engineering
CdS Ternary Heterojunctions [1,40,128,129,130,131,132,133,134,135,136,137,138,139,140,141,142,143,144,145,146,147]	Z-scheme (Bi_2_S_3_/CdS, BiVO_4_/CdS), S-scheme (CdS@ZIF-67, NaNbO_3_/CdS), Dual cocatalyst (Ag-PdS/ZnS/CdS	ZOI: 15–44 mm; MIC: 2–11 mg/mL; *E. coli*: 90–99.9% kill; *S. aureus*: 90–99% kill	Z-scheme/S-scheme charge transfer; cascade electron transfer; synergistic ROS generation (•OH, •O_2_^−^, ^1^O_2_)	Superior performance; 93–99% inactivation within 10–60 min; preserved redox potentials; enhanced stability (10–15 cycles); very low Cd^2+^ release (<3%)	Complex interface engineering; high synthesis costs; limited scalability; band alignment constraints	vs. ZnS/Ag_2_S Z-scheme: CdS-based S-scheme heterojunctions show superior charge separation and stability; both systems require precise band alignment; ZnS-based systems offer better photostability but limited visible-light response
Doped CdS Systems [1,44,92,93,94,95,96,97,98,99,100,101,102,103,104,105,106,107,108,109,110,111,112,113,114,115,116,117,118,119,120,121,122,123,124,125,126,127,128,129,130,131,132,133,134,135,136,137,138,139,140,141,142,143,144,145,146,147]	Mn-CdS, Co-doped CdS, Ni-Ce co-doped CdS, Cu:CdS, Zr-doped CdS, Nb-doped CdS	ZOI: 7–31 mm; MIC: 0.01–5 mg/mL; *E. coli*: moderate–high; *S. aureus*: moderate–high	Bandgap narrowing; defect-mediated charge separation; enhanced ROS generation; modified ion release	Tuneable bandgap (2.06–2.72 eV); controllable band edge positions; transformed Gram-selective to broad-spectrum; improved charge separation	Dopant distribution control; potential secondary toxicity; reduced crystallinity; complex synthesis optimization	vs. ZnS doping: CdS responds more strongly to doping due to its softer lattice; dopants more easily incorporated; vs. Bi_2_S_3_ doping: CdS offers wider doping tunability due to established synthetic protocol
CdS Hybrid/Encapsulated Systems [1,44,67,68,69,70,71,72,73,74,75,76,77,78,79,80,81,82,83,84,85,86,87,88,89,90,91,92,93,94,95,96,97,98,99,100,101,102,103,104,105,106,107,108,109,110,111,112,113,114,115,116,117,118,119,120,121,122,123,124,125,126,127,128,129,130,131,132,133,134,135,136,137,138,139,140,141,142,143,144]	Polymer (PEG, PVP, CTAB, PANI), Biopolymer (Chitosan, Pectin, DNA), Mesoporous carriers (SiO_2_, MCM-41, HNTs), Core–shell (Fe_3_O_4_@CdS)	ZOI: 2–35 mm; MIC: 5–1000 µg/mL; *E. coli*: 70–95% kill; *S. aureus*: 70–100% kill	Controlled Cd^2+^ release; surface passivation; enhanced biocompatibility; matrix-mediated ROS generation	Excellent stability (8–12 cycles); enhanced biocompatibility (<5% hemolysis); improved colloidal stability; reduced Cd^2+^ release (<10%)	Lower intrinsic activity due to shell thickness; potential matrix degradation; reduced surface accessibility; complex synthesis	vs. ZnS polymer composites: CdS systems generally more active but less stable; polymer encapsulation more critical for CdS due to toxicity concerns; both systems benefit from biopolymer capping

## Data Availability

No primary research results, software, or code have been included, and no new data were generated or analyzed as part of this review. Data sharing is not applicable to this article.

## Abbreviations

The following abbreviations are used in this manuscript:

CdS	Cadmium sulfide
CBM	Conduction band minimum
CBD	Chemical bath deposition
QD	Quantum dots
DNA	Deoxyribonucleic acid
DFT	Density functional theory
EPA	Environmental Protection Agency
H_2_O_2_	Hydrogen peroxide
•OH	Hydroxyl radical
LPS	Lipopolysaccharide
MDR	Multidrug-resistant
MIC	minimum inhibitory concentration
ROS	Reactive oxygen species
REACH	Registration, Evaluation, Authorization and Restriction of Chemicals regulation
SILAR	Successive ionic layer adsorption and reaction
O_2_•^−^	Superoxide anions
^1^O_2_	Singlet oxygen
VBM	Valence band maximum
WHO	World Health Organization
ZOI	Zone of inhibition
0D	Zero-dimensional
1D	One-dimensional
2D	Two-dimensional
3D	Three-dimensional
